# 12,23-Dione dammarane triterpenes from *Gynostemma longipes* and their muscle cell proliferation activities via activation of the AMPK pathway

**DOI:** 10.1038/s41598-018-37808-9

**Published:** 2019-02-04

**Authors:** Thi Kim Quy Ha, Ha Thanh Tung Pham, Hyo Moon Cho, Van On Tran, Jun-Li Yang, Da-Woon Jung, Darren R. Williams, Won Keun Oh

**Affiliations:** 1 Korea Bioactive Natural Material Bank, Research Institute of Pharmaceutical Sciences, College of Pharmacy, Seoul National University, Seoul, 08826 Republic of Korea; 2 Hanoi University of Pharmacy, Hanoi, Vietnam; 3 Key Laboratory of Chemistry of Northwestern Plant Resources of CAS and Key Laboratory for Natural Medicine of Gansu Province, Lanzhou Institute of Chemical Physics, Chinese Academy of Sciences, Lanzhou, 730000 P. R. China; 4 New Drug Targets Laboratory, School of Life Sciences, Gwangju Institute of Science and Technology, 1 Oryong-Dong, Buk-Gu, Gwangju, 61005 Republic of Korea

## Abstract

The aging population is growing rapidly around the world and there is also an increase in sarcopenia, which is characterized by decreased muscle mass, strength and function in the elderly population. AMP-activated protein kinase (AMPK) is an essential sensor and regulator of glucose, lipid and energy metabolism throughout the body. Previous studies have shown that AMPK pathway activation by regular exercise and appropriate dietary control have beneficial effects on skeletal muscle. In the process of searching for new AMPK activators from medicinal plants, we isolated and characterized eight new 12,23-dione dammarane triterpenoids (**1**–**3** and **5**–**9**), as well as one known gypentonoside A from *Gynostemma longipes*. When all isolates were tested for their AMPK activation activities, seven compounds (**1** and **3**–**8**) were significantly activated AMPK phosphorylation in mouse C2C12 skeletal muscle cell lines. Since *G. longipes* contained a significant amount of active compound **1** (over 2.08% per dried raw plant), it suggested the potential of this plant to be developed as a functional food or botanical drug that enhances muscle proliferation by activating AMPK signaling pathways.

## Introduction

One of the most distinctive effects of aging is the involuntary loss of muscle mass, strength and function, which is termed sarcopenia^1^. Skeletal muscle decreases approximately 3–8% per decade after 30 years of age, and muscle loss rapidly increases by about 25–30% after 60 years of age^2^. This loss of muscle mass, strength and function is the key cause of many diseases in elderly people, and the increased risk of injury and falls due to sarcopenia can also lead to continuous functional dependence and disability. The decreased muscle mass is also associated with a continuous increase in fat mass and consequently a change in body composition, and this in turn is associated with a higher incidence of insulin resistance in the elderly people^3,4^. Therefore, an increase of muscle mass in the elderly population is a promising approach to alleviate many of the symptoms of geriatric diseases^5^.

Recent studies suggested that muscle loss can be prevented by exercise in both young and older peoples^6^. Conversely, increased physical inactivity and obesity due to a sedentary lifestyle are associated with attenuated muscle mass and reduced AMP-activated protein kinase (AMPK) activity^7,8^. AMPK plays an important role in the regulation of cellular energy homeostasis, and the activation of AMPK promotes glucose uptake, fatty acid oxidation, mitochondrial biogenesis and insulin sensitivity. In eukaryotes, there are three subunits of the AMPK, including the catalytic *α*-subunit (*α*1 and *α*2), regulatory *β*-subunit (*β*1 and *β*2) and *γ*-subunit (*γ*1, *γ*2 and *γ*3)^9,10^. The regenerative process in skeletal muscle tissue generally involves a coordinated sequence of the activation of satellite cells, which proliferate to produce a population of myogenic progenitors (termed myoblasts)^7,11,12^. Especially, the AMPKα1 isoform has an important role in enhancing Warburg-like glycolysis through non-canonical sonic hedgehog (Shh) signaling in myogenic cells during muscle regeneration. AMPK*α*1 knock-out in satellite cells reduces their activation and also inhibit the proliferation and differentiation of myogenic progenitor cells^7^. Taken together, the activation of AMPK (especially AMPK*α*1) is a key mediator linking obesity and impaired muscle regeneration, providing an effective drug target to facilitate muscle regeneration in obese and elderly populations^7–9^.

Our ethnopharmacological investigation of medicinal plants with AMPK activation activity resulted in the selection of *Gynostemma longipes*, commonly called “That diep dom”, a Vietnamese traditional herb used widely by the local community for tonic, treatment of diabetes and health strengthening. A fraction from this plant was then found to show potential activity in accelerating the muscle proliferation in the myoblast C2C12 cell model. The main ingredients of *G. longipes* are known as dammarane triterpenes^13,14^ similar to other species of *Gynostemma* genus^15,16^. Dammarane triterpenes have been also reported to play a significant role in the development of natural drugs for the treatment of different metabolic diseases such as diabetes mellitus, metabolic syndrome, aging and neurodegenerative diseases^17^. In this study, bioassay-guided fractionation and isolation of *G. longipes* were performed, and resulted in the purification of nine 12,23-dione dammarane triterpenes (**1**–**9**). All isolates were evaluated for their enhancement effects on muscle proliferation through activation of the AMPK pathway using the C2C12 myoblast cell model.

## Results and Discussion

### Plant authentication and structural determination of new compounds

**Figure 1:**
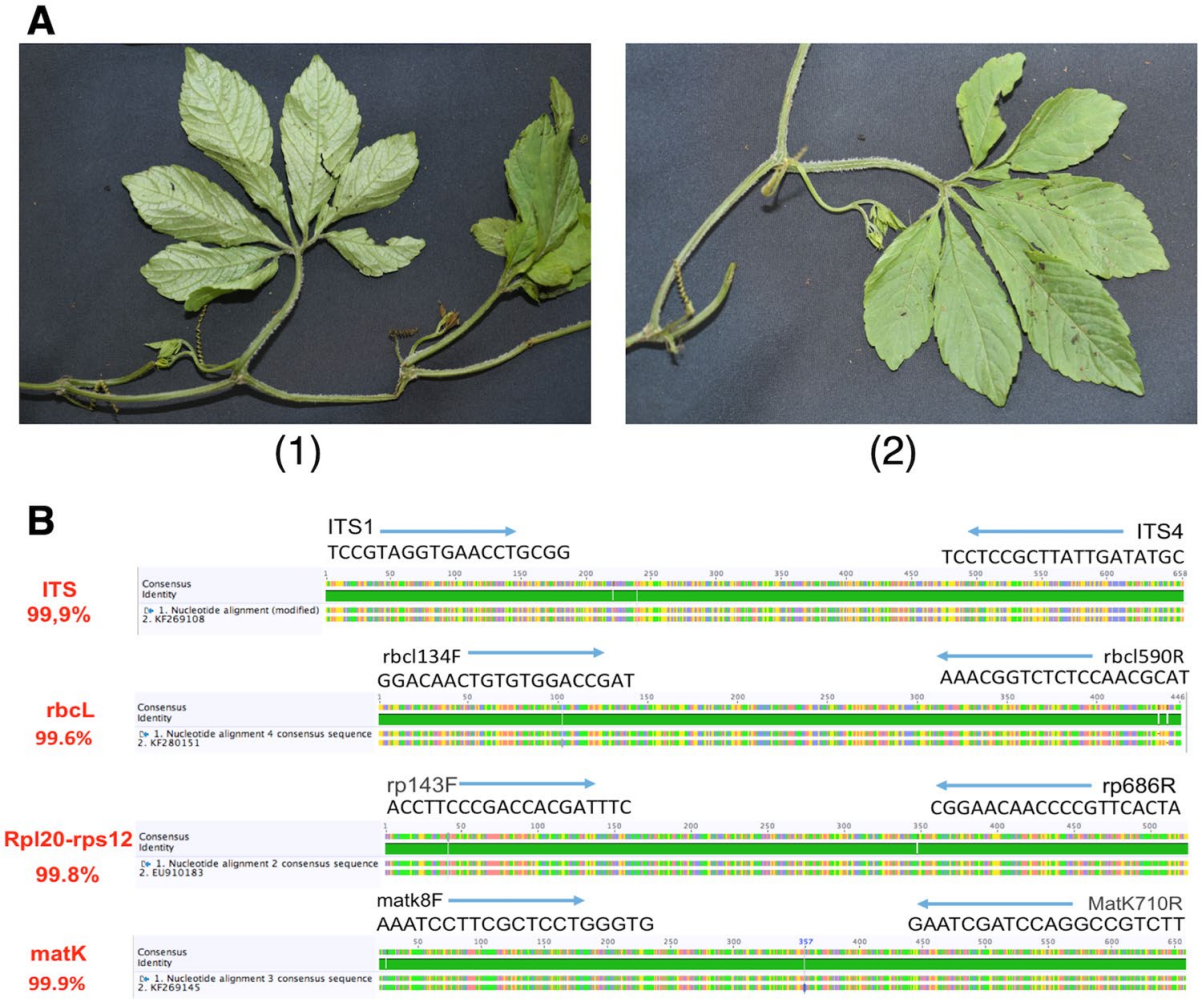
Morphological and DNA authentication of *G. longipes*. (**A**) Authentication of *G. longipes* based on morphology (1) whole plant with abaxial leaves and (2) adaxial leaf. (**B**) DNA analysis of *G. longpipes* with 4 DNA sequences ITS, rbcL, rpl20-rps12 and matK. All sequences of *Gynostemma longipes* were found to be at least 99.5% identical to sequences registered on Genebank.

The authentication of medicinal plants based on genomic tools is an efficient and accurate method in the past decades. In this study, *G. longipes* was successfully identified using the DNA barcoding marker techniques with 4 DNA sequences. Result in Fig. 1 indicated that these DNA barcodes including internal transcribed spacer (ITS), ribulose-bisphosphate carboxylase (rbcL), ribosomal protein L20 - ribosomal protein S12 (rpl20-rps12), and maturase K (matK) were found to be similar 99.9%, 99.6%, 99.8%, and 99.9% compared to sequences registered on Genebank, respectively). Therefore, the large quantities of this plant were harvested and further investigated the chemical constituents and bioactivities.

**Figure 2:**
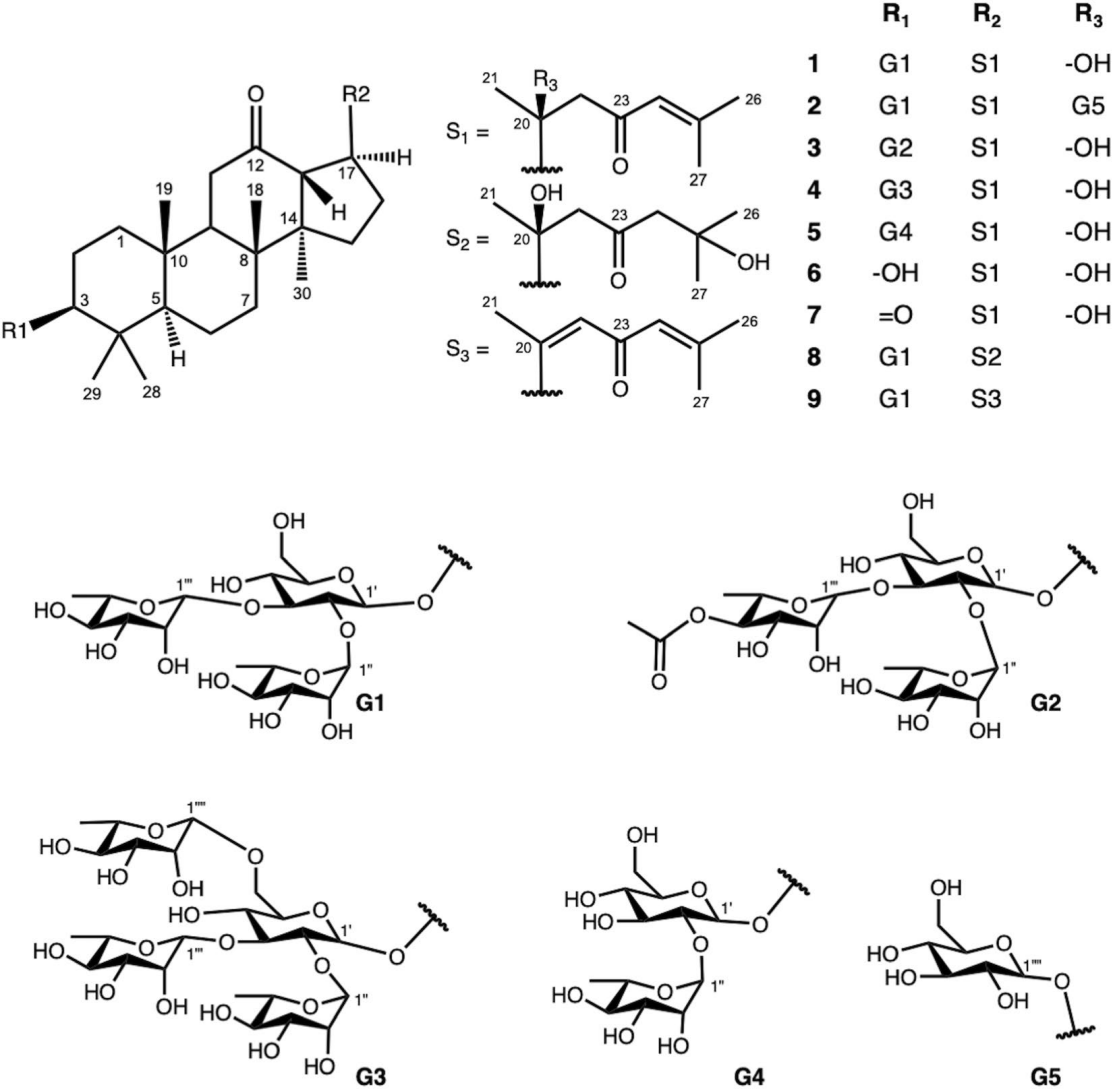
Chemical structures of nine compounds (**1–9**) isolated from *G. longipes*.

Bioassay-guided fractionation and isolation of the bioactive fraction from the leaves of *G. longipes* afforded eight new dammarane triterpenes (**1–3** and **5–9**), along with one known compound gypentonoside A (**4**) (Fig. 2).

**Figure 3:**
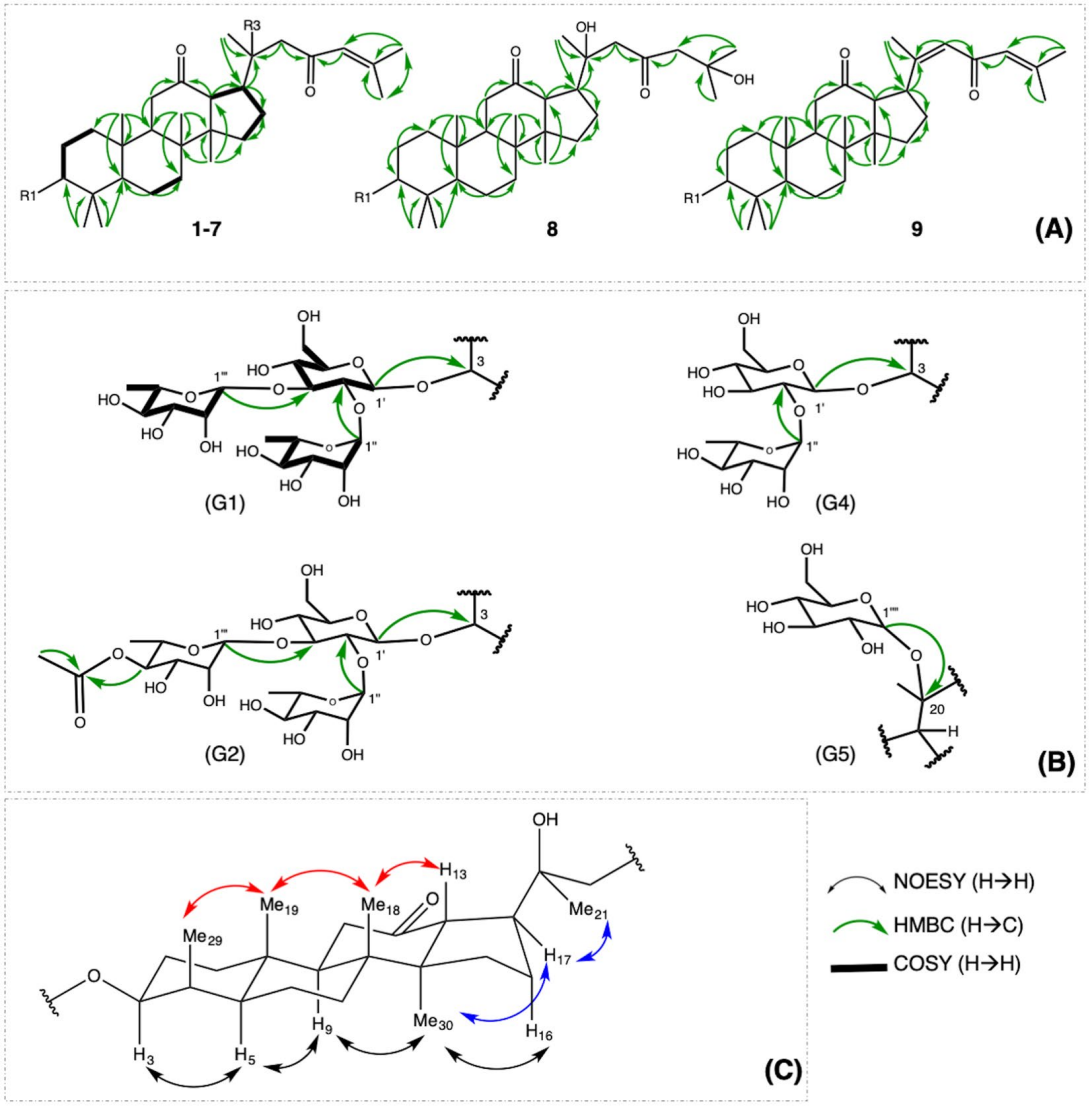
Key HMBC (from H → C) and COSY (H → H) correlations of aglycones (**A**), glycoside chains (**B**) of new compounds and key NOESY correlation of aglycone part of compound **1** (**C**).

Compound **1** was obtained as a white crystal with ${[\alpha ]}_{{\rm{D}}}^{25}$ −32.0 (*c* 0.1, MeOH). The molecular formula of compound **1** was deduced as C_48_H_78_O_17_ from the [M − H]^−^ high resolution electrospray ionization mass spectrometry (HRESIMS) ion peak observed at *m*/*z* 925.5157 (calcd for C_48_H_77_O_17_, 925.5166) (Supplementary Fig. S3). Infrared (IR) absorption at 1616 and 1697 cm^–1^ and ultraviolet–visible (UV) peak at 254 nm suggested the existence of an *α*,*β*-unsaturated carbonyl group. The IR broad peak at 3404 cm^–1^ appeared in companion with the peak at 1035 cm^–1^ indicated the presence of a hydroxyl group. The proton nuclear magnetic resonance (^1^H NMR) spectrum demonstrated oxygenated methine proton at *δ*_H_ 3.25 (1 H, H-3) and eight methyl singlets at *δ*_H_ 2.15, 1.70, 1.50, 1.17, 1.12, 1.11, 0.84, and 0.76 (each 3 H) (Supplementary Fig. S4). Its carbon-13 nuclear magnetic resonance (^13^C NMR) spectrum revealed two ketone carbons (*δ*_C_ 211.7 and 201.7) and one oxygenated quaternary carbons (*δ*_C_ 74.0) (Supplementary Fig. S5). The above observations strongly suggested that compound **1** is a dammarane triterpenoid with one hydroxyl and two ketone groups. The position of hydroxyl group was confirmed at C-20 by heteronuclear multiple bond correlation (HMBC) correlations from H-17 (*δ*_H_ 2.77), H-21 (*δ*_H_ 1.50) and H-22 (*δ*_H_ 2.90 and 2.72) to C-20 (*δ*_C_ 74.0). One ketone group was located at C-12 from the HMBC experiment showing cross-peaks from H-11 (*δ*_H_ 2.24 and 2.22), H-13 (*δ*_H_ 3.26, d, *J* = 9.6 Hz), and H-17 (*δ*_H_ 2.32) to C-12 (*δ*_C_ 211.7) (Supplementary Fig. S7). As suggested by UV and IR spectra, an *α*,*β*-unsaturated ketone moiety was further confirmed at C-23 (C = O) and C-24/25 (CH = C) by HMBC cross peaks from H-22 (*δ*_H_ 2.90, 2.72), H-24 (*δ*_H_ 6.28) to C-23 (*δ*_C_ 201.7), from H-22 (*δ*_H_ 2.90, 2.72), H-26 (*δ*_H_ 2.15), and H-27 (*δ*_H_ 1.71) to C-24 (*δ*_C_ 126.4) (Fig. 3A). The nuclear overhauser effect spectroscopy (NOESY) correlation of H-3 (*δ*_H_ 3.25) and H-5 (*δ*_H_ 0.66) demonstrated an *α* orientation for H-3. Both H-17 and Me-21 were also determined as α orientation based on the coupling constant of H-13 (*J = *9.6 Hz) and NOESY correlations from H-17 (*δ*_H_ 2.77) to H-21 (*δ*_H_ 1.50) and H-30 (*δ*_H_ 0.84) (Fig. 3C and Supplementary Fig. S9). Therefore, the aglycone of 1 was deduced as 3*β*,20(*S*)-dihydroxydammar-24-en-12,23-dione. The diagnostic NMR data suggested the existence of three sugar units in compound 1. Acid hydrolysis of **1** with HCl afforded d-glucopyranose and l-rhamnopyranose, based on liquid chromatography analysis following treatment with l-cysteine methyl ester hydrochloride and benzyl isothiocyanate derivatization. The coupling patterns of anomeric protons indicated *β* configuration for the glucopyranosyl (*δ*_H_ 4.80, *J* = 7.7 Hz) unit and *α* configuration for the two rhamnopyranosyl (*δ*_H_ 5.92, 5.70, br s) units. The HPLC-MS experiment at the positive mode showed fragment ions at 909 [M + H − H_2_O (18)]^+^, 763 [M + H − H_2_O – rha (146)]^+^, 617 [M + H − H_2_O – rha – rha (146)]^+^, 455 [M + H − H_2_O – 2 × rha – glu (162)]^+^ corresponding to the presence of a sugar chain consisting of 2 rhamnoses and 1 glucose. The linkage patterns of three sugar units were analyzed based on the HMBC experiment (Fig. 3B and Supplementary Fig. S7) which also constructed the connection between H-1′ (*δ*_H_ 4.80) and C-3 (*δ*_C_ 88.5), H-1″ (*δ*_H_ 5.92) and C-2′ (*δ*_C_ 78.3), H-1′′′ (*δ*_H_ 5.70) and C-3′ (*δ*_C_ 87.5). Therefore, compound **1** was elucidated as 3*β*,20(*S*)-dihydroxydammar-24-en-12,23-dione-3-*O*-*α*-l-rhamnopyranosyl-(1→2)-[*α*-l-rhamnopyranosyl-(1→3)]-*β*-d-glucopyranoside.

Compound **2** was obtained as a white and amorphous powder. Its molecular formula of C_54_H_88_O_22_ was evident by the negative HRESIMS [M − H]^−^ ion peak at *m/z* 1087.5682 (calcd for C_54_H_87_O_22_, 1087.5694) (Supplementary Fig. S10). The high-performance liquid chromatography-mass spectrometry (HPLC-MS) experiment in the positive mode showed a similar fragmentation with compound **1** with extra neutral loss of one hexose (162). The ^1^H, ^13^C NMR, and heteronuclear single quantum correlation (HSQC) spectroscopic data of 2 (Tables 1, 2 and Supplementary Figs S11–S13) were similar in resonance to those of **1** except for an additional *β*-glucopyranosyl unit which can be proved by the resonance of anomeric proton H-1ʹʹʹʹ (*δ*_H_ 5.16, d, *J* = 7.7 Hz) and the addition of 6 carbon resonances at C-1ʹʹʹʹ (*δ*_C_ 98.8), C-2ʹʹʹʹ (*δ*_C_ 73.0), C-3ʹʹʹʹ (*δ*_C_ 72.9), C-4ʹʹʹʹ (*δ*_C_ 74.0), C-5ʹʹʹʹ (*δ*_C_ 78.2), and C-6ʹʹʹʹ (*δ*_C_ 63.3). When compared with 1, a downfield shift of C-20 (*δ*_C_ 80.7, + 6.7 ppm) and an upfield shift of C-21 (*δ*_C_ 23.7, −3.5 ppm) were observed, which supported the attachment of the glucopyranosyl unit to C-20. Further HMBC analysis also constructed a link between H-1ʹʹʹʹ (*δ*_H_ 5.16) and C-20 (Fig. 3B and Supplementary Fig. S14). Accordingly, compound **2** was determined to be 3*β*,20(*S*)-dihydroxydammar-24-en-12,23-dione-3-*O*-*α*-l-rhamnopyranosyl-(1→2)-[*α*-l-rhamnopyranosyl-(1→3)]-*β*-d-glucopyranosyl-20-*O*-*β*-d-glucopyranoside.

**Table 1:** ^1^H chemical shifts [*δ*_H_ in ppm, Mult. (*J* in Hz)] of compounds **1–3** and **5–9**.

H	Compounds							
	1^c*^	2^*ac#*^	3^*bc#*^	5^c*^	6^*c#*^	7^c*^	8^c*^	9^*c#*^
1	1.20, overlap	1.18, overlap	1.20, overlap	1.22, overlap	1.20, overlap	1.58, m	1.24, m	1.22, overlap
	0.69, overlap	0.69, overlap	0.69, overlap	0.73, overlap	0.69, overlap	1.23, m	0.75, m	0.73, overlap
2	2.11, overlap	2.12, overlap	2.11, overlap	2.23, overlap	2.10, overlap	2.48, m	2.15, m	2.04, m
	1.70, overlap	1.71, overlap	1.70, overlap	1.80, overlap	1.70, overlap	2.42, m	1.75, m	1.56, overlap
3	3.25, overlap	3.26, dd (11.2, 4.4)	3.32, dd (11.2, 4.4)	3.32, dd (11.6, 4.0)	3.40, dd (10.8, 5.4)		3.28, dd (11.7, 4.1)	3.30, dd (11.7, 4.2)
5	0.66, overlap	0.65, overlap	0.66, m	0.67, d (9.6)	0.80, overlap	1.35, m	0.69, m	0.69, overlap
6	1.50, overlap	1.49, overlap	1.50, m	1.49, m, overlap	1.50, overlap	1.50, m	1.54, m	1.52, m
	1.39, overlap	1.40, overlap	1.39, m	1.41, m, overlap	1.39, overlap	1.41, m	1.41, m	1.42, m
7	1.39, overlap	1.38, overlap	1.39, m	1.42, m, overlap	1.39, overlap	1.43, m	1.41, m	1.45, m
	1.25, overlap	1.25, overlap	1.25, m	1.26, m, overlap	1.25, overlap	1.28, m	1.27, m	1.27, m
9	1.62, overlap	1.79, overlap	1.62, m	1.65 (dd, 13.0, 4.0)	1.62, overlap	1.78, m	1.66, overlap	1.62, m
11	2.24, overlap	2.24, overlap	2.24, overlap	2.23, overlap (2H)	2.34, overlap	2.37, overlap	2.24, overlap	2.27, overlap
	2.22, dd (10.5, 3.1)	2.22, overlap	2.22, dd (10.5, 3.1)		2.32, overlap	2.28, dd (10.5, 3.8)	2.22, overlap	2.21, overlap
13	3.26, d (9.6)	3.66, d (9.4)	3.28, d (9.6)	3.27, d (10.0)	3.31, d (9.6)	3.31, d (9.6)	3.20, d (9.7)	3.04, d (10.5)
15	1.15, overlap	1.12, overlap	1.15, overlap	1.16, m, overlap	1.17, overlap	1.17, m	1.80, m	1.45, m
	2.07, overlap	1.97, overlap	2.07, overlap	1.84, m, overlap	1.88, overlap	1.87, m	1.14, m	1.27, m
16	2.07, overlap	2.30, overlap	2.07, overlap	2.10, m, overlap	2.10, overlap	2.12, m	2.02, m	2.07, m
	1.91, overlap	2.00, overlap	1.91, overlap	1.93, m, overlap	1.92, overlap	1.93, m	1,88, m	1.91, m
17	2.77, td (10.0, 5.1)	3.12, td (10.0, 5.1)	2.80, td (10.0, 5.0)	2.80, td (10.0, 5.1)	2.80, td (10.0, 5.1)	2.8, td (5.2, 9.6)	2.78, td (10.1, 5.3)	3.20, ddd (17.0, 10.6, 6.7)
18	1.12, s	1.28, s	1.11, s	1.13, s	1.19, s	1.19, s	1.12, s	1.15, s
19	0.76, s	0.76, s	0.82, s	0.79, s	0.85, s	0.83, s	0.78, s	0.81, s
21	1.50, s	1.80, s	1.53, s	1.53, s	1.54, s	1.54, s	1.53, s	2.34, s
22	2.90, d (14.2)	3.38, d (14.2)	2.90, d (14.2)	2.93, d (14.2)	2.94, d (14.2)	2.94, d (14.2)	3.11, d (15.6)	6.47, s
	2.72, d (14.2)	3.21, d (14.2)	2.72, d (14.2)	2.74, d (14.2)	2.74, d (14.2)	2.75, d (14.2)	2.83, d (15.6)	
24	6.28, s	6.22, s	6.30, s	6.31, brs	6.32, s	6.31, s	2.96, d (14.8)	6.18, s
							2.95, d (14.8)	
26	2.15, s	2.14, s	2.17, s	2.17, s	2.17, s	2.17, s	1.46, s	2.23, s
27	1.70, s	1.67, s	1.71, s	1.71, s	1.72, s	1.72, s	1.46, s	1.70, s
28	1.17, s	1.17, s	1.19, s	1.24, s	1.23, s	1.14, s	1.19, s	1.22, s
29	1.11, s	1.11, s	1.11, s	1.19, s	1.04, s	1.05, s	1.12, s	1.16, s
30	0.84, s	0.83, s	0.86, s	0.86, s	0.88, s	0.89, s	0.83, s	0.89, s
1ʹ	4.80, d (7.7)	4.82, d (7.5)	4.84, d (7.7)	4.96, d (7.7)			4.82, d (7.4)	4.86, d (7.7)
2ʹ	4.01, overlap	4.01, overlap	4.06, overlap	4.30, overlap			4.03, overlap	4.07, overlap
3ʹ	4.11, overlap	4.11, overlap	4.11, overlap	4.29, overlap			4.12, t (8.0)	4.11, t (8.0)
4ʹ	4.01, overlap	4.01, overlap	4.01, overlap	4.16, t (9.1)			4.02, overlap	4.07, overlap
5ʹ	3.85, overlap	3.87, overlap	3.89, overlap	3.98, overlap			3.85, m	3.89, m
6ʹ	4.48, overlap	4.53, overlap	4.61, overlap	4.61, dd (12.0, 2.4)			4.46, dd (11.8, 3.4)	4.60, overlap
	4.33, overlap	4.43, overlap	4.47, overlap	4.41, dd (12.0, 6.6)			4.32, dd (11.8, 5.4)	4.52, overlap
1ʹʹ	5.92, br s	5.95, br s	5.99, br s	6.59, brs			5.94, br s	6.00, br s
2ʹʹ	4.66, overlap	4.66, overlap	4.72, overlap	4.88, m			4.66, overlap	4.72, overlap
3ʹʹ	4.45, overlap	4.45, overlap	4.48, overlap	4.70, dd (9.4, 3.4)			4.45, overlap	4.50, overlap
4ʹʹ	4.26, overlap	4.28, overlap	5.79, t (10.0)	4.35, overlap			4.25, t (9.4)	4.31, overlap
5ʹʹ	4.61, overlap	4.62, overlap	4.74, overlap	4.81, dq (9.5, 6.0)			4.66, overlap	4.68, overlap
6ʹʹ	1.64, d (6.0)	1.65, d (6.0)	1.41, d (6.0)	1.73, d (6.0)			1.66, d (6.0)	1.70, d (6.0)
1ʹʹʹ	5.70, br s	5.72, br s	5.72, br s				5.70, br s	5.77, br s
2ʹʹʹ	4.87, br s	4.87, br s	4.84, br s				4.87, br s	4.93, br s
3ʹʹʹ	4.53, overlap	4.52, overlap	4.53, overlap				4.53, dd (9.5, 3.1)	4.59, overlap
4ʹʹʹ	4.28, overlap	4.30, overlap	4.30, overlap				4.26, t (9.4)	4.32, overlap
5ʹʹʹ	4.70, overlap	4.69, overlap	4.72, overlap				4.66, overlap	4.75, overlap
6ʹʹʹ	1.60, d (6.0)	1.62, d (6.0)	1.63, d (6.0)				1.61, d (6.0)	1.66, d (6.0)

^a^Chemical shift of ***20-0-glu***: H-1ʹʹʹʹ [5.16, d (7.76)], H-2ʹʹʹʹ [4.53, overlap], C-3ʹʹʹʹ [4.62, overlap], C-4ʹʹʹʹ [4.35, overlap], C-5ʹʹʹʹ [3.93, overlap], C-6ʹʹʹʹ [4.52, overlap]; ^*b*^Chemical shift of ***3″-Acetyl***: CH_3_ (2.10, s); ^*c*^Measured in C_5_D_5_N-*d*_5_; ^#^NMR-600 MHz; ^*^NMR-500 MHz.

**Table 2:** ^13^C chemical shifts (*δ*_C_ in ppm) of compounds **1–3** and **5–9**.

C	1^c*^	2^ac#^	3^bc#^	5^c*^	6^c#^	7^c*^	8^c*^	9^c*^
1	39.3, CH_2_	39.4, CH_2_	39.4, CH_2_	39.4, CH_2_	39.3, CH_2_	39.5, CH_2_	39.4, CH_2_	39.4, CH_2_
2	26.9, CH_2_	26.9, CH_2_	27.0, CH_2_	27.1, CH_2_	29.0, CH_2_	34.4, CH_2_	26.9, CH_2_	28.7, CH_2_
3	88.5, CH	88.6, CH	88.6, CH	88.8, CH	78.1, CH	216.2, C	88.7, CH	88.7, CH
4	39.9, C	39.9, C	39.9, C	40.0, C	39.9, C	47.7, C	40.0, C	39.8, C
5	56.6, CH	56.6, CH	56.7, CH	56.7, CH	56.7, CH	55.3, CH	56.7, CH	56.6, CH
6	18.8, CH_2_	18.8, CH_2_	18.8, CH_2_	18.9, CH_2_	19.2, CH_2_	20.3, CH_2_	18.9, CH_2_	19.0, CH_2_
7	34.8, CH_2_	35.0, CH_2_	34.8, CH_2_	34.9, CH_2_	34.9, CH_2_	34.1, CH_2_	34.9, CH_2_	34.8, CH_2_
8	40.9, C	41.1, C	41.0, C	41.0, C	41.0, C	40.9, C	41.1, C	41.0, C
9	54.6, CH	54.9, CH	54.8, CH	54.7, CH	54.9, CH	54.0, CH	54.7, CH	54.1, CH
10	37.7, C	37.7, C	37.7, C	37.7, C	38.2, C	37.7, C	37.8, C	37.7, C
11	40.1, CH_2_	40.3, CH	40.1, CH_2_	40.2, CH	40.3, CH_2_	40.3, CH_2_	40.2, CH	39.8, CH_2_
12	211.7, C	211.4, C	211.8, C	211.8, C	211.9, C	211.2, C	211.9, C	209.9, C
13	56.2, CH	56.5, CH	56.3, CH	56.3, CH	56.3, CH	56.8, CH	56.9, CH	59.0, CH
14	56.5, C	56.5, C	56.5, C	56.7, C	56.7, C	56.3, C	56.3, C	55.3, C
15	32.2, CH_2_	32.6, CH_2_	32.2, CH_2_	32.3, CH_2_	32.3, CH_2_	32.3, CH_2_	32.2, CH_2_	32.2, CH_2_
16	25.2, CH_2_	25.3, CH_2_	25.2, CH_2_	25.2, CH_2_	25.2, CH_2_	25.2, CH_2_	25.2, CH_2_	28.7, CH_2_
17	45.2, CH	43.5, CH	45.2, CH	45.3, CH	45.3, CH	45.2, CH	45.4, CH	45.7, CH
18	16.0, CH_3_	16.2, CH_3_	16.0, CH_3_	16.1, CH_3_	16.1, CH_3_	15.7, CH_3_	16.0, CH_3_	16.1, CH_3_
19	16.5, CH_3_	16.6, CH_3_	16.5, CH_3_	16.5, CH_3_	16.5, CH_3_	16.0, CH_3_	16.5, CH_3_	16.4, CH_3_
20	74.0, C	80.7, C	74.1, C	74.1, C	74.1, C	74.1, C	73.9, C	159.7, C
21	27.2, CH_3_	23.7, CH_3_	27.2, CH_3_	27.3, CH_3_	27.2, CH_3_	27.3, CH_3_	27.0, CH_3_	17.7, CH_3_
22	54.8, CH_2_	55.1, CH_2_	55.0, CH_2_	55.0, CH_2_	55.0, CH_2_	55.0, CH_2_	55.1, CH_2_	126.3, CH
23	201.7, C	199.8, C	201.7, C	201.7, C	201.8, C	201.7, C	212.9, CH_2_	191.7, C
24	126.4, CH	126.6, CH	126.5, CH	126.6, CH	126.6, CH	126.5, CH	54.8, CH	127.5, CH
25	154.9, C	154.5, C	154.9, C	154.9, C	154.9, C	155.0, C	70.0, C	154.1, C
26	20.9, CH_3_	20.8, CH_3_	21.0, CH_3_	21.0, CH_3_	21.0, CH_3_	21.0, CH_3_	30.8, CH_3_	20.8, CH_3_
27	27.7, CH_3_	27.6, CH_3_	27.7, CH_3_	27.7, CH_3_	27.7, CH_3_	27.7, CH_3_	30.4, CH_3_	27.7, CH_3_
28	28.1, CH_3_	28.1, CH_3_	28.2, CH_3_	28.3, CH_3_	28.4, CH_3_	27.0, CH_3_	28.2, CH_3_	28.2, CH_3_
29	16.9, CH_3_	16.9, CH_3_	16.8, CH_3_	17.2, CH_3_	16.6, CH_3_	21.5, CH_3_	17.0, CH_3_	17.1, CH_3_
30	17.4, CH_3_	17.3, CH_3_	17.5, CH_3_	17.5, CH_3_	17.5, CH_3_	17.3, CH_3_	17.5.CH_3_	17.7, CH_3_
1ʹ	105.3, CH	105.3, CH	105.1, CH	105.8, CH			105.3, CH	105.4, CH
2ʹ	78.3, CH	78.5, CH	78.6, CH	78.1, CH			78.4, CH	78.5, CH
3ʹ	87.5, CH	87.6, CH	87.8, CH	80.3, CH			87.8, CH	87.7, CH
4ʹ	70.6, CH	70.4, CH	70.6, CH	72.5, CH			70.8, CH	70.8, CH
5ʹ	78.3, CH	78.3, CH	78.6, CH	78.7, CH			78.4, CH	78.4, CH
6ʹ	62.8, CH_2_	62.8, CH	62.8, CH_2_	63.3, CH_2_			63.0, CH_2_	63.0, CH_2_
1ʹʹ	102.5, CH	102.4, CH	101.9, CH	102.1, CH			102.5, CH	102.6, CH
2ʹʹ	72.2, CH	71.0, CH	70.3, CH	72.8, CH			72.4, CH	72.5, CH
3ʹʹ	72.7, CH	72.7, CH	72.3, CH	72.9, CH			72.8, CH	72.9, CH
4ʹʹ	73.9, CH	73.9, CH	76.0, CH	74.5, CH			74.1, CH	74.1, CH
5ʹʹ	70.5, CH	70.6, CH	67.6, CH	69.9, CH			70.5, CH	70.6, CH
6ʹʹ	18.9, CH_3_	18.9, CH_3_	18.2, CH_3_	19.1, CH_3_			18.9, CH_3_	18.9, CH_3_
1ʹʹʹ	103.9, CH	104.0, CH	104.1, CH				104.0, CH	104.1, CH
2ʹʹʹ	72.7, CH	72.6, CH	72.7, CH				72.9, CH	72.7, CH
3ʹʹʹ	72.9, CH	73.0, CH	72.9, CH				73.0, CH	73.0, CH
4ʹʹʹ	73.8, CH	73.8, CH	73.8, CH				73.9, CH	74.0, CH
5ʹʹʹ	71.0, CH	71.0, CH	71.1, CH				71.1, CH	71.2, CH
6ʹʹʹ	18.7, CH_3_	18.7, CH_3_	18.7, CH_3_				18.7, CH_3_	18.8, CH_3_

^a^Chemical shift of ***20-0-glu***: C-1ʹʹʹʹ (98.8, CH), C-2ʹʹʹʹ (73.0, CH), C-3ʹʹʹʹ (72.9, CH), C-4ʹʹʹʹ (74.0, CH), C-5ʹʹʹʹ (78.2, CH), C-6ʹʹʹʹ (63.3, CH_2_); ^*b*^Chemical shift of ***3*″*-Acetyl***: 171.9 (C = 0); 21.4 (CH_3_); ^*c*^Measured in C_5_D_5_N-*d*_5_;

^#^NMR-600 MHz; ^*^NMR-500 MHz.

Compound **3** was obtained as a white and amorphous powder. The negative HRESIMS showed the deprotonated molecular ion peak at *m/z* 967.5276 [M − H]^−^ (calcd for C_50_H_79_O_18_, 967.5272) indicating that the molecular formula is C_50_H_80_O_18_ (Supplementary Fig. S16). HPLC-MS experiments at the positive mode showed the presence of an oligosaccharide chain consisted of 2 rhamnoses and **1** glucose similar with compound **1** together with an acetyl substitution. The ^1^H, ^13^C, and HSQC NMR spectroscopic data of **3** (Tables 1, 2 and Supplementary Figs S17–S19) also showed similar resonances to those of **1**, apart from an additional acetyl unit which can be deduced by the addition of **2** carbon resonances of one ketone (*δ*_C_ 171.9) and a methyl singlet (*δ*_C_ 21.4; *δ*_H_ 2.10). When compared to **1**, a downfield shift of C-4ʹʹ (*δ*_C_ 76.0, +2.1 ppm) and an upfield shift of C-5ʹʹ (*δ*_C_ 67.6, −2.9 ppm) demonstrated the substitution of an acetyl moiety to C-4ʹʹ. The HMBC spectrum showed a cross peak from H-4ʹʹ (*δ*_H_ 5.79) to acetyl carbonyl (*δ*_C_ 171.9) confirming the linkage (Fig. 3B and Supplementary Fig. S20). Accordingly, compound **3** was determined as 3*β*,20(*S*)-dihydroxydammar-24-en-12,23-dione-**3**-*O*-*α*-l-rhamnopyranosyl-(1→2)-[(4-*O*-acetyl)-*α*-l-rhamnopyranosyl-(1→3)]-*β*-d-glucopyranoside.

Compound **5** was obtained as a white and amorphous powder. The negative HRESIMS showed a deprotonated molecular ion peak at *m/z* 779.4597 [M − H]^−^ (calcd for C_42_H_67_O_13_, 779.4587) indicating the molecular formula to be C_42_H_68_O_13_ (Supplementary Fig. S21). The HPLC-MS experiment at positive mode showed fragment ions at 763 [M + H − H_2_O (18)]^+^, 617 [M + H − H_2_O − rha (146)]^+^, 455 [M + H − H_2_O – rha − glc (162)]^+^ corresponding to the existence of a oligosaccharide chain [-glc-rha]. The ^1^H, ^13^C, HSQC and HMBC NMR spectroscopic data of **5** (Tables 1, 2 and Supplementary Figs S22–S26) could be discriminated with **1** by the lack of resonances of the rhamnopyranosyl unit attached to C-3ʹ and the shielded chemical shift of this carbon (*δ*_C_ 80.3). Accordingly, compound **5** was determined as 3*β*,20(*S*)-dihydroxydammar-24-en-12,23-dione-3-*O*-*α*-l-rhamnopyranosyl-(1→2)-*β*-d-glucopyranoside.

Compound **6** was obtained as a white and amorphous powder. The negative HRESIMS showed a deprotonated molecular ion peak at *m*/*z* 471.3393 [M − H]^−^ (calcd C_30_H_47_O_4_, 471.3480), representing the molecular formula C_30_H_48_O_4_ (Supplementary Fig. S27)_._ The ^1^H, ^13^C NMR, HSQC and HMBC spectroscopic data (Tables 1, 2 and Supplementary Figs S28–S31) shown that **6** is the aglycone of compounds **1−4**. The NOESY correlations from H-3 (*δ*_H_ 3.40) to H-5 (*δ*_H_ 0.80) and H-28 (*δ*_H_ 0.77) suggested OH-3 to be *β*-oriented, which was also supported by the coupling pattern of H-3 (dd, *J* = 9.2 and 2.6 Hz) (Fig. 3C). As a consequence, compound **6** was identified as 3*β*,20(*S)*-dihydroxydammar-24-en-12,23-dione.

Compound **7** was obtained as a white and amorphous powder. The negative HRESIMS showed a deprotonated molecular ion peak at *m*/*z* 471.3452 [M + H]^+^ (calcd for C_30_H_47_O_4_, 471.3469), which represents the molecular formula C_30_H_46_O_4_ (Supplementary Fig. S32). The ^1^H, ^13^C, HSQC, and HMBC NMR spectroscopic data of **7** (Tables 1, 2 and Supplementary Figs S33−S36) showed similar resonances to those of 6 except for the presence of the third ketone (*δ*_C_ 216.2) and the disappearance of H-3 resonance, together with the deshielded chemical shift of C-2 (*δ*_C_ 34.4, +5.4 ppm) and C-4 (*δ*_C_ 47.7, +7.8 ppm) suggested ketone oxidation of OH-3. Thus, **7** was identified as 20(*S*)-hydroxydammar-24-en-3,12,23-trione.

Compound **8** was obtained as an white, amorphous powder, and its molecular formula of C_48_H_80_O_18_ was evident on the basis of a negative [M − H]^−^ HRESIMS ion peak at *m/z* 943.5374 (calcd for C_48_H_79_O_18_, 943.5380) (Supplementary Fig. S37). HPLC-MS experiments at positive mode suggested the existence of a sugar chain similar with compound **1**, together with the presence of two hydroxyl groups substituted on the its aglycone. The ^1^H, ^13^C NMR, HSQC spectroscopic data of **8** (Tables 1, 2 and Supplementary Figs S38−S40) showed similar resonances to those of **1** except for the hydration of double bonds at C-24 and C-25. When compared with **1**, an upfield shift of C-24 (*δ*_C_ 54.8, −71.6 ppm), C-25 (*δ*_C_ 70.0, −84.8 ppm) and a downfield shift of C-23 (*δ*_C_ 212.9, +11.2 ppm) were observed, which supported the attachment a hydroxyl group at C-25. Further HMBC analysis also established the connection between Me-26, Me-27 (each *δ*_H_ 1.46, s), and C-25 (*δ*_C_ 70.0) (Fig. 3A and Supplementary Fig. S41). Thus, compound **8** was determined to be 3*β*,20(*S*),25-trihydroxydammaran-12,23-dione 3-*O*-*α*-l-rhamnopyranosyl-(1→2)-[*α*-l-rhamnopyranosyl-(1→3)]-*β*-d-glucopyranoside.

Compound **9** was obtained as a white and amorphous powder. The molecular formula of compound **9** was deduced as C_48_H_76_O_16_ from the [M − H]^−^ HRESIMS ion peak observed at *m*/*z* 907.5066 (calcd for C_48_H_75_O_16_, 907.5061). The HPLC-MS experiment at positive mode also demonstrated the presence of a glycoside chain identical with compound **1**. The ^1^H, ^13^C, and HSQC NMR spectroscopic data of **9** (Tables 1, 2 and Supplementary Figs S43−S45) showed a similar resonance to **1**. The difference was that 9 lacked a hydroxyl group signal at C-20 and contained a olefinic linkage resonance. Moreover, when compared with 1, an upfield shift of C-21 (*δ*_C_ 17.7, −9.5 ppm), and downfield shifts of C-20 (*δ*_C_ 159.7, +85.7 ppm) and C-22 (*δ*_C_ 126.3, +71.5 ppm) were observed, suggesting the dehydration of this compound at C-20. The HMBC spectrum exhibited cross peaks from H-21 and H-17 to C-20 and C-22, respectively (Fig. 3A and Supplementary Fig. S44), further confirming the proposed structure. Thus, compound 9 was determined as 3*β*-hydroxydammar-20,24-dien-12,23-dione-3-*O*-*α*-l-rhamnopyranosyl-(1→3)-[*α*-l-rhamnopyranosyl-(1→2)]-*β*-d-glucopyranoside.

### Quantification of compound 1

Extraction yields by using different solvents 100% MeOH, water, 30% EtOH, 50% EtOH, 70% EtOH and EtOH were 11.5%, 16.5%, 18.5%, 17.5%, 14%, and 7.5%, respectively. As a result of the quantitative analysis, the content of compound **1** as main ingredient was the highest as 130.01 mg/g in 70% EtOH extract. This was equivalent to 20.75 mg/g of the raw material of *G. longipes*.

### Dammarane triterpenes enhanced muscle proliferation through activating AMPK

**Figure 4:**
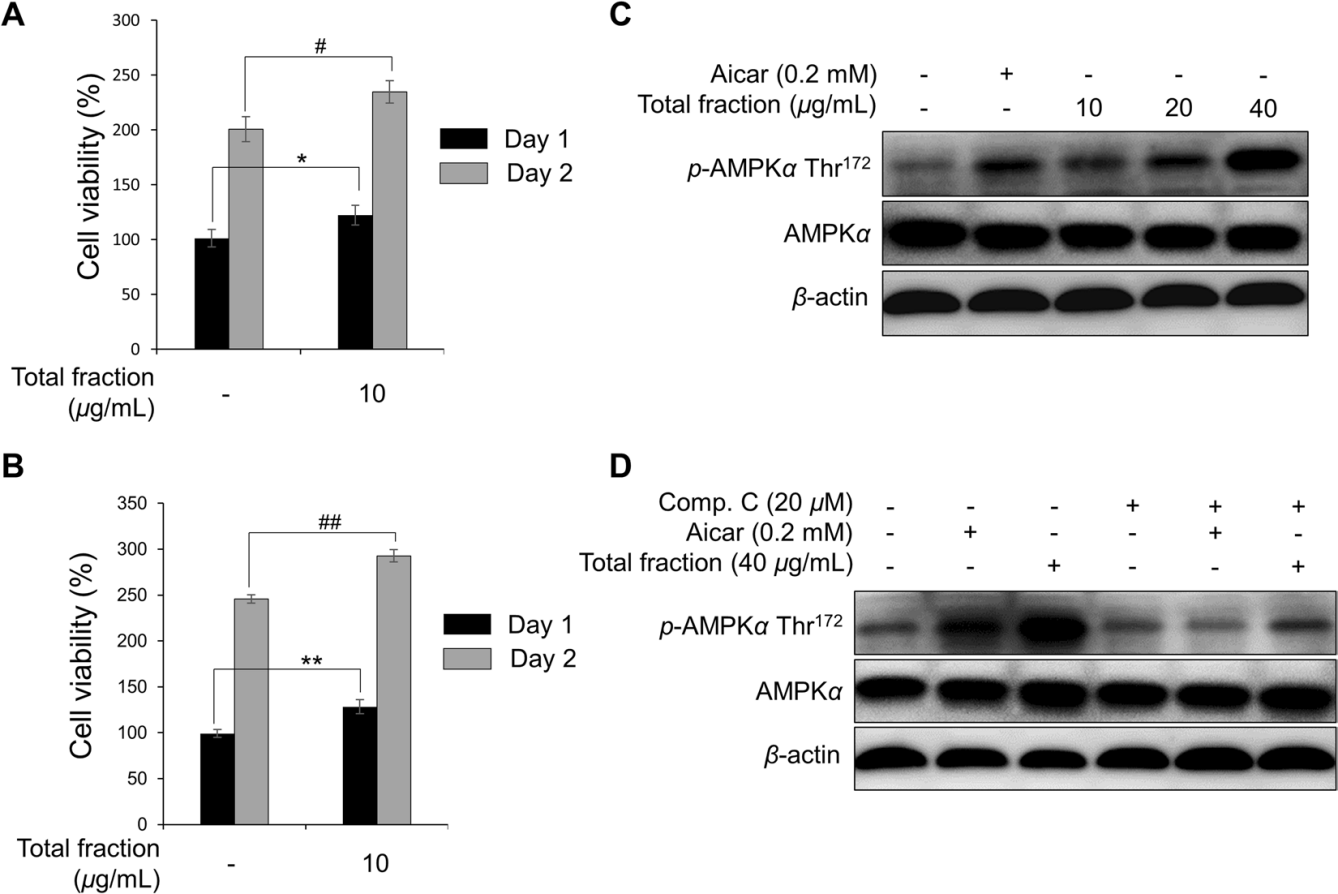
Effect of the SP70-EtOH 95% fraction from *G. longipes* on C2C12 myoblast cell proliferation using the MTT method (**A**) or WST method (**B**) after 1 and 2 days incubation. Data are presented as the mean ± SD (*n* = 3), **p < 0.05 and ^##^*p* < 0.01, compared to the negative control group. (**C**) *G*. *longipes* induced phosphorylation (Thr172) of AMPK*α* in a concentration-dependent manner when C2C12 myotubes were incubated with the SP70-EtOH 95% fraction for 30 minutes. (**D**) C2C12 myotubes were preincubated for 15 minutes with or without compound C before treatment with fractions or Aicar as a positive control. After 30 minutes of incubation, cells were harvested and Western blot performed to evaluate the expression of *p*-AMPK.

Regeneration of damaged skeletal muscles depends on satellite cells (known as quiescent muscle precursor cells), which play an important role in the proliferation and differentiation of myoblasts to form or repair muscle fibers^18,19^. Recently, several studies have demonstrated that decreased proliferation of myoblasts and cytotoxicity can reduce the number of muscle fibers^20–22^. Therefore, mouse C2C12 cells, also known as myoblastic cells, were chosen in this research because they are a valid model to study muscle cell proliferation^7,20^. In a search for new bioactive natural products with promising activity for muscle proliferation, we found that the 95% EtOH eluted fraction on SP70 resin of the total extract could potentially be a ‘hit’ using an *in vitro* screening method. This fraction stimulated an increase in proliferation of C2C12 myoblast cell about 20–30% compared to the negative control during 24 and 48 hours of treatment using 3-(4,5-dimethyl-2-thiazolyl)-2,5-diphenyl-2H-tetrazolium bromide (MTT) and 4-[3-(4-iodophenyl)-2-(4-nitrophenyl)-2H-5-tetrazolio]-1,3-benzene disulfonate (WST-1) methods (Fig. 4A,B). The proliferation also expressed dose-dependently using a cell counting method (Supplementary Fig. S47). As AMPK*α* is an important regulator during the process of muscle proliferation^7,23^, we tested the effect of the *G. longipes* fraction on *p*-AMPK*α* stimulation in C2C12 myotubes and myoblasts. As a result, this fraction stimulated the phosphorylation of AMPK significantly in a concentration-dependent manner (Fig. 4C,D and Supplementary Figs S48,S49, and S53). On the other hand, when the cells were pre-incubated with compound C (dorsomorphin – a reversible and potent AMPK inhibitor), the *G. longipes* fraction did not affect the expression of *p*-AMPK*α*, similarly to the vehicle-treated cells.

**Figure 5:**
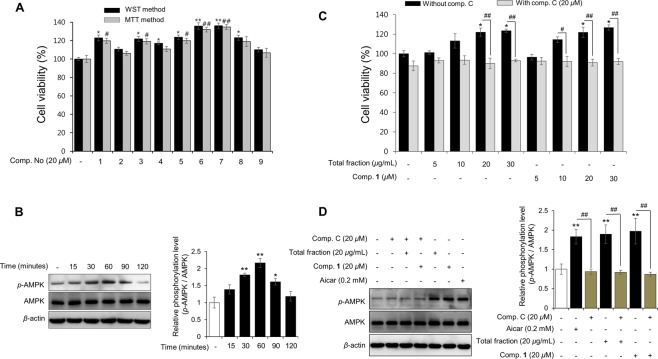
(**A**) Effect of isolated compounds (**1–9**) on the proliferation of myoblast cells. C2C12 cells were incubated with tested compounds for 48 hours and cell viability was assessed using the MTT or WST method. Each value is expressed as the mean ± SD (*n* = 3); *p < 0.05 and **p < 0.01, compared to negative control group using the WST method; while ^#^*p* < 0.05 and ^##^*p* < 0.01, compared to the vehicle group using the MTT method. (**B**) C2C12 myoblast cells were exposed to compound **1** (20 *μ*M) and incubated for 15 to 120 minutes. Phosphorylation of AMPK protein in the cells was assessed by Western blot analysis. Values are expressed as the mean ± SD (*n* = 3), *p < 0.05 and **p < 0.01, compared to the negative control group. (**C**) The effect of co-treatment compound C with the active fraction and compound **1** on the cell viability. C2C12 myoblasts were re-treated with compound C (20 *μ*M) for 15 minutes and then the cells were continuously incubated with test samples for 2 days. After that, the percentage of cell viability was evaluated using MTT method. Results are presented as the mean ± SD (*n* = 3); *p < 0.05 and **p < 0.01, compared to negative control group (without compound C treatment); while ^#^*p* < 0.05 and ^##^*p* < 0.01 compared to co-treatment with compound C and test samples group, respectively. (**D**) Down-regulatory effect of compound C on *p-*AMPK (Thr^172^) when co-treatment with the active fraction or compound **1**. After 15 minutes re-incubated with compound C (20 *μ*M), mouse C2C12 myoblasts were then exposed to test samples for 1 hour. Phosphorylation of AMPK protein was measured by Western blotting. Data were calculated as the mean ± SD (*n* = 3); **p < 0.01 compared to the negative control, while ^##^*p* < 0.01 compared to co-treatment with compound C and test samples group, respectively.

Bioassay-guided isolation from *G. longipes* resulted in the isolation of nine compounds (**1–9**) and all of the isolates were evaluated for their enhancing effect on muscle proliferation in C2C12 myoblast cells. After 48 hours of treatment, cell viability was assessed using the MTT and WST-1 methods. Seven compounds (**1** and **3**–**8**) increased cell viability by about 20–30% (Fig. 5A). Notably, compounds **6** and **7** exhibited the highest induction of cell proliferation at a value from 131.95 ± 2.54% to 136.19 ± 2.73%. Since compound **1** was identified as a major constituent of this plant (over 2.08% of the material), it was selected to examine the effects on the proliferation of two cancer cell lines, MCF-7 and MDA-MB-231 breast cancer cells. As shown in Supplementary Fig. S50, cell viability was not changed when the cells were incubated with a fraction of *G. longipes* (10 *μ*g/mL) or compound **1** (10 *μ*M) for 2 days. These results suggested that the active fraction and major compound of *G. longipes* stimulate muscle cell proliferation selectively and do not induce the outgrowth of harmful cancer cell lines.

A number of studies demonstrated that dammarane triterpenoids are potent classes of AMPK-activating agents^16,17,24^. In order to evaluate the potential activities of compounds from *G. longipes*, nine isolates (**1–9**) were measured the stimulatory effects on the *p*-AMPK and *p*-ACC expressions using mouse C2C12 myotubes. Results in Supplementary Figs S51 and S52 indicated that compounds **5**, **6**, and **7** increased the phosphorylation of AMPK and ACC proteins strongly, while compounds **1**, **3**, **4**, and **8** exhibited less efficient. Therefore, potential candidates (**1**, **5**, **6**, and **7**) were further confirmed the activation effect on *p*-AMPK in C2C12 myoblasts. These compounds clearly increased the phosphorylation of the target protein compared to Aicar as a positive control (Supplementary Fig. S53).

Because compound **1** is the major constituent of the active fraction, it was selected for further evaluating the activation of *p*-AMPK in a time-dependent manner using the mouse C2C12 myoblast model. Remarkably, compound **1** up-regulated the phosphorylation of AMPK from 30 to 90 minutes (Fig. 5B and Supplementary Fig. S54). Besides, compound **1** also induced the expression of AMPK slightly at 24 hours after treatment (Supplementary Figs S55 and S56). These results suggested that triterpenoids from *G. longipes* could significantly stimulate the *p*-AMPK expression in both mouse C2C12 myotubes and myoblasts.

Considering the structures and activities of all nine isolates, the core structure of the dammarane skeleton might contribute mainly to the AMPK activation effect. Further examination of structure-activity relationships (SAR) revealed that compounds with a longer saccharide side chain at C-3 showed lower AMPK inhibition effects: **4** (4 sugar units) < **1** (3 sugar units) < **5** (2 sugar units) < **6** and **7** (no sugar). It was also noteworthy that with the same number of sugar units, the attachment of a sugar unit to C-20 remarkably reduced the activity (**2** < **4**). Furthermore, the hydration of olefinic bond at C-20 and C-24 slightly increased the effect in the following order: **9** (two double bonds) < **1** (one double bond) < **8** (no double bond). The change of hydroxy (**6**) and ketone substitutions (**7**) at C-3 did not cause any difference in activity.

Finally, to assess the effects of compound C during muscle proliferation, the cell growth and expression of *p*-AMPK were analyzed using the mouse C2C12 myoblast model. Interestingly, co-treatment of compound C with the active fraction or compound **1** decreased the percentage of cell viability after 48 hours of incubation, compared to the number of C2C12 cells exposed to the fraction or compound **1** alone (Fig. 5C). The decline of the *p*-AMPK expression was also observed after 1 hour of incubation. Similarly, the stimulation effect of compound **1** on *p*-AMPK was down-regulated by co-treatment with compound C (Fig. 5D and Supplementary Fig. S49). Consequently, the activation of the AMPK signaling pathway by *G. longipes*’s fraction and compound **1** contributes relatively to muscle proliferation by inducing the growth of C2C12 myoblasts.

### Effects of dammarane triterpenes on DNA synthesis during cell proliferation

**Figure 6:**
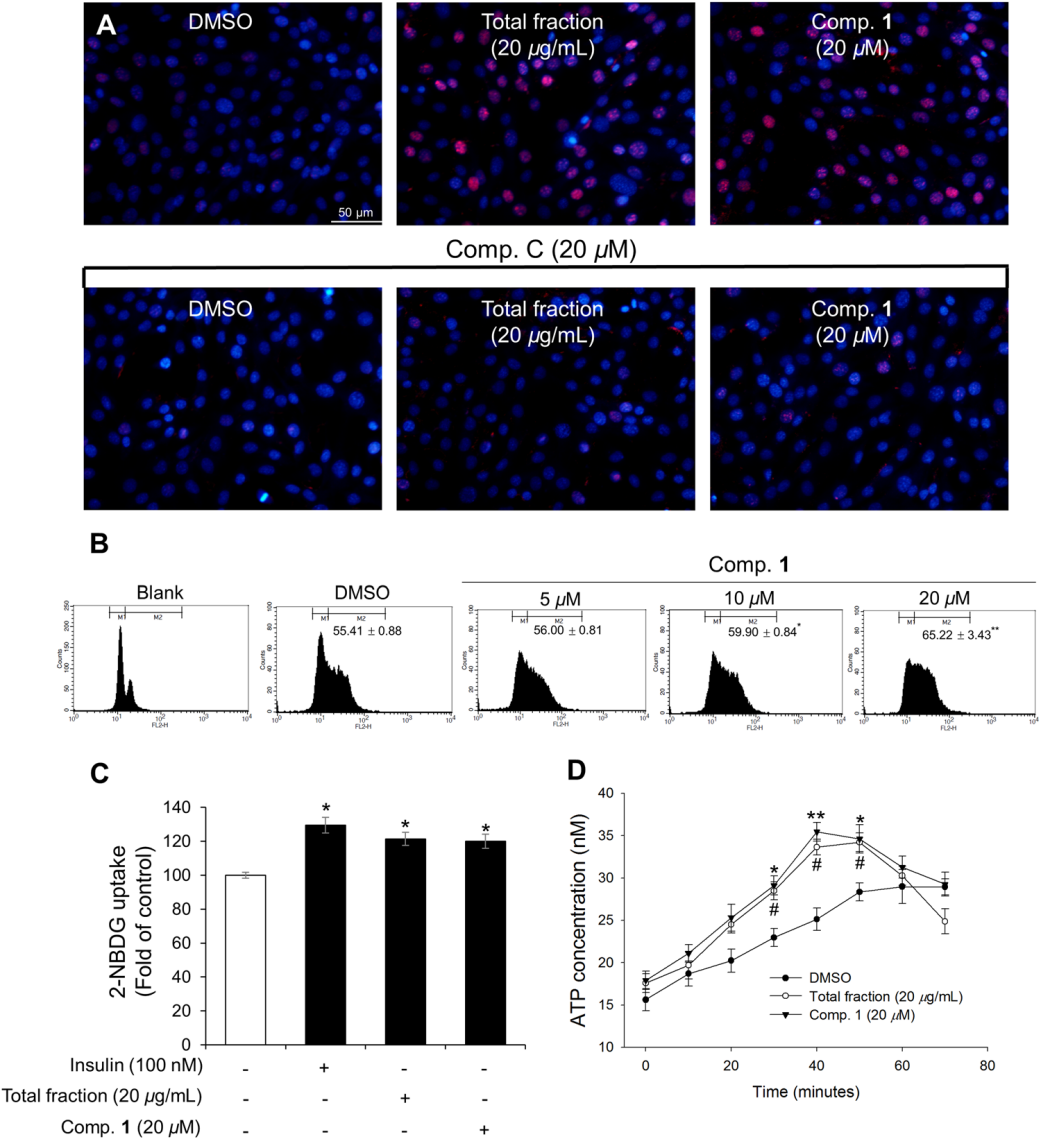
Increased DNA synthesis in C2C12 myoblasts cells by dammarane triterpenes from *G. longipes*. (**A**) C2C12 myoblast cells were re-treated with compound C (20 *μ*M) for 15 minutes and the cells were then incubated with the active fraction (20 *μ*g/mL) or compound **1** (20 *μ*M) for 8 hours. Cells were then incubated for 2 hours in the presence or absence of BrdU. After fixation and permeabilization, the cells were incubated with antibodies and stained with the DAPI solution. Cells images were observed by fluorescence microscopy. (**B**) C2C12 myoblast cells were incubated with compound **1** at different concentrations for 8 hours. After incubation for 2 hours with or without BrdU, the cells were harvested and flow cytometric analysis for BrdU and PI staining was performed. The cell distribution was displayed using a histogram. Data presented as the mean ± SD (*n* = 2), *p < 0.05 and **p < 0.01, compared to the DMSO group. (**C**) Stimulation effects of the active fraction or compound **1** on glucose uptake in C2C12 myoblasts using the 2-NBDG probe. The cells were incubated with test samples or insulin (as a positive control) for 1 hour. The fluorescent intensity was observed at ex/em = 450/535 nm using a fluorescence microplate reader. Each value is represented as the mean ± SD (*n* = 3), *p < 0.05 compared to negative control. (**D**) Dammarane triterpenes from *G. longipes* increased intracellular ATP level of C2C12 myoblasts. The cells were treated with the active fraction (20 *μ*g/mL) or compound **1** (20 *μ*M) from 0 to 70 minutes. Mitochondrial ATP synthesis was measured using ATP bioluminescence determination assay. Data were calculated as the mean ± SD (*n* = 3); the *p* value for compound **1** (*p < 0.05, **p < 0.01) and total fraction (^#^*p* < 0.05) compared to negative control.

It has been previously reported that 5-bromo-2′-deoxyuridine (BrdU) can be used to label the DNA of cells in the S-phase. This method is useful for quantifying the degree of DNA synthesis during cell proliferation^25^. Therefore, in order to confirm whether compound **1** from *G. longipes* induces cell proliferation, immunochemical staining analysis with a BrdU antibody was performed. In the first experiment, C2C12 myoblast cells were treated with compound **1** (20 *μ*M) or the SP70-EtOH 95% fraction (20 *μ*g/mL) and incubated for 8 hours. Next, the cells were incubated for 2 hours with or without BrdU. The BrdU incorporation into the nucleus was assessed using a fluorescein isothiocyanate (FITC)-conjugated anti-BrdU antibody and DAPI staining. As shown in Fig. 6A and Supplementary Fig. S57, the number of BrdU-positive cells was significantly increased in the group treated with the active fraction of *G. longipes* or compound **1** compared to DMSO treatment alone. We also evaluated the BrdU-labeled cells using a flow cytometer for BrdU and PI staining. When C2C12 cells were incubated with or without BrdU treatment, the fluorescence intensity was significantly increased from 33.46 ± 0.58% to 55.41 ± 0.88%. In order to confirm the concentration dependence in the cells, compound **1** was treated for 8 hours at different concentrations (20, 10, and 5 *μ*M) and then incubated with BrdU for 2 hours. The BrdU positive cell population increased in a concentration-dependent manner to 65.22 ± 3.43%, 59.90 ± 0.84% and 56.00 ± 0.81%, respectively (Fig. 6B).

Moreover, to better understand the effects of co-treatment compound C with test samples on DNA synthesis during cell proliferation, immunofluorescence detection and flow cytometry analysis for BrdU staining were carried out. For qualitative analysis, the number of red stained cells notably reduced in the groups co-treated with compound C compared to the active fraction or compound **1** alone (Fig. 6A and Supplementary Fig. S57). To further examine the fluorescence intensity of each group, the BrdU-labeled cells were determined using flow cytometry for BrdU staining only (Supplementary Fig. S58). This results indicated that co-treatment of compound C with the active fraction or compound **1** failed to increase the number of BrdU-positive cells.

### Effects of dammarane triterpenes on glucose uptake and ATP levels

Recent studies suggested the importance of AMPK*α* enhances glucose uptake and ATP production during the process of muscle proliferation^7^. Considering that the glucose uptake level was evaluated using a fluorescent-tagged glucose analogue, 2-[*N*-(7-nitrobenz-2-oxa-1,3-diazol-4-yl)amino]-2-deoxy-d-glucose (2-NBDG), to monitor intracellular glucose uptake. As illustrated in Fig. 6C, the active fraction and compound **1** as well as insulin (as a positive control) increased glucose uptake in mouse C2C12 myoblasts.

The effects of dammarane triterpenes from *G. longipes* on the mitochondrial ATP synthesis was examined by treatment mouse C2C12 myoblasts with the active fraction and compound **1** in approximately 70 minutes. The ATP levels of the active fraction and compound **1** increased drastically from 30 to 50 minutes comparison with the vehicle group (Fig. 6D). However, the rate of ATP production reduced rapidly after 1 hour of incubation with these samples. During the proliferation of myoblasts, the production of ATP is an essential downstream target of glycolysis, and cells used these ATP (approximately 30%) which is supplied by oxidative phosphorylation (OXPHOS) as the major source of metabolic energy^26^. Taken together, the role of triterpenoids from *G. longipes*, which regulate muscle proliferation by increasing glucose uptake and ATP production has been suggested.

### Enhancement of cell proliferation by dammarane triterpenes through cell cycle regulation

Several previous studies have proposed that ginseng root extract promotes glucose uptake in muscle cells and also induces the proliferation of *β*-cells^27^, and ginsenoside Rg_1_ from *Panax ginseng* enhances myoblast differentiation and myotube growth^28^. Thus, the effect of dammarane triterpenoid from *G. longipes* on cell proliferation through regulation of cell cycle was also evaluated using propidium iodide (PI) staining method^29^. C2C12 myoblasts were exposed to compound **1** (20 μM) for 12 and 24 hours. The cell cycle distributions at different phases were significantly changed, compared to the vehicle group. After 12 hours of incubation with compound **1**, the cells with 2 N DNA content (G_0_/G_1_ phases) decreased remarkably from 72.07 ± 0.63% to 65.09 ± 2.49%, while the cells with >2 N DNA content (S or G_2_/M phases) increased about 8% compared with negative control (Supplementary Fig. S59). Moreover, the proportion of G_2_/M phases increased significantly from 13.64 ± 1.47% to 22.41 ± 0.81% when the cells were incubated with compound **1** for 24 hours in comparison with the group treated with DMSO. In our study, we have described novel natural product compounds that enhance myoblast proliferation. It can be noted that previous research indicates that compounds promoting skeletal muscle stem cell proliferation also have the potential to enhance muscle regeneration *in vivo*^30^. As a further study, we believe that assessment of the novel compounds described in this manuscript as promoters of muscle regeneration is warranted.

## Conclusion

**Figure 7:**
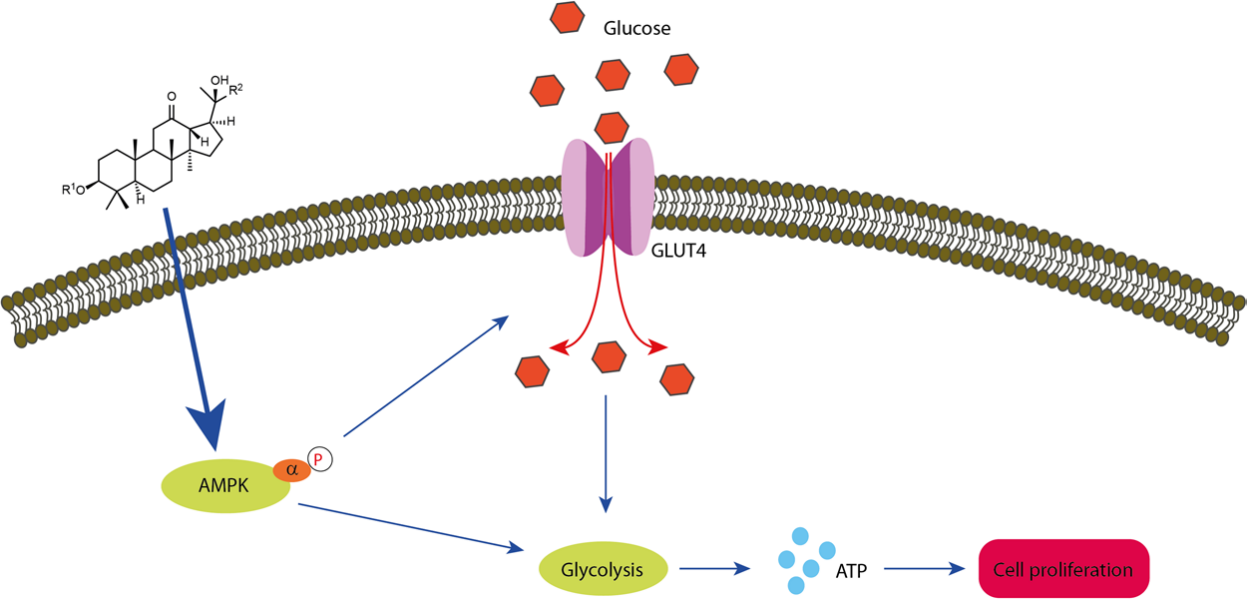
The proposed mechanism for enhancing cell proliferation by dammarane triterpenoids from *G. longipes* through AMPK pathway.

In this study, we reported the findings of eight new 12,23-dione dammarane triterpenes from *G. longipes*, a traditional Vietnamese medicinal plant. Seven of the compounds (**1** and **3–8**) activated significantly AMPK phosphorylation in mouse C2C12 cell lines. The result of AMPK activation by compound **1** could stimulate glucose uptake into the cells and increase mitochondrial ATP synthesis. The effect of dammarane triterpenes on DNA synthesis during cell proliferation also showed that compound **1** increased the BrdU positive cell population in a dose-dependent manner. Analysis of cell cycle using flow cytometry displayed clearly that compound **1** reduced the 2 N DNA content (G_0_/G_1_ phases) while the ratio of G_2_/M phases was significantly increased. These results suggested strongly that compound **1** enhances significantly the proliferation of C2C12 myoblast cells (Fig. 7). Furthermore, a considerable amount of active compound **l** (over 2.08% per dried raw plant) in *G. longipes* suggested that it may be a promising candidate for development of functional food or botanical drug. These results also indicated that new dammarane-type compounds are promising candidates for muscle proliferation via activation of AMPK signaling pathways and could be further studied and developed as therapeutics for geriatric diseases.

## Methods

### General experimental procedures

Optical rotations were measured in a JASCO P-2000 polarimeter (JASCO International Co. Ltd., Tokyo, Japan). IR data were recorded on a Nicolet 6700 FT-IR spectrometer (Thermo Electron Corp., Waltham, MA, USA). NMR data were analyzed using an AVANCE 500 MHz spectrometer (Bruker, Germany) or a JNM-ECA 600 MHz spectrometer (Jeol, Japan). HRESIMS values were analyzed using an Agilent Technologies 6130 Quadrupole LC/MS spectrometer equipped with an Agilent Technologies 1260 Infinity LC system (Agilent Technologies, Inc., Santa Clara, CA, USA) and an INNO C18 column (4.6 × 150 mm, 5 μm particle size, 12 nm, J.K.Shah & Company, Korea). Silica gel (particle size: 63−200 μm) and RP-C_18_ (particle size: 40–63 μm) were purchased from Merck (Darmstadt, Germany) and Sephadex LH-20 from Sigma-Aldrich (St. Louis, MO, USA) were used for column chromatography (CC). Silica gel 60 F_254_ and RP-18 F_254_ TLC plates were obtained from Merck (Darmstadt, Germany). A Gilson HPLC purification system, equipped with an Optima Pak C_18_ column (10 × 250 mm, 10 μm particle size; RS Tech, Seoul, Korea), was used at a flow rate of 2 mL/min and UV detection at 205 and 254 nm.

### Plant material

Plant material was collected in the Ha Giang province of Vietnam in March 2016. The sample was identified as *G. longipes* C. Y. Wu based on the morphological characteristics by Prof. Tran Van On, Head of Department of Botany, Hanoi University of Pharmacy, Hanoi, Vietnam. A voucher specimen was deposited in the Medicinal Herbarium of Hanoi Univeristy of Pharmacy (HNIP) with the accession number HNIP.18500/16 (Fig. 1).

### Plant authentication of *Gynostemma longipes* by DNA bio-marker

Total DNA was extracted from 200 mg of fresh plant leaves using the Dneasy Plant Mini Kit (QIAGEN, Germany) with some modifications. PCR amplification and sequencing were performed by Macrogen Inc. (Seoul, Korea) using 4 pairs of primers: ITS1-F (5′-TCCGTAGGTGAACCTGCGG-3′), ITS4-R (5′- TCCTCCGCTTATTGATATGC-3′) for ITS1–5.8S-ITS2 region; matK8-F (5′-AAATCCTTCGCTCCTGGGTG-3′), matK710-R (5′-GAATCGATCCAGGCCGTCTT-3′) for partial maturase K gene, rbcL134-F (5′-GGACAACTGTGTGGACCGAT-3′), rbcL590-R (5′-AAACGGTCTCTCCAACGCAT-3′) for partial ribulose-1,5-bisphosphate carboxylase/oxygenase large subunit (rbcL) gene and rp143-F (5′-ACCTTCCCGACCACGATTTC-3′), rp686-R (5′-CGGAACAACCCCGTTCACTA-3′) for rpl20-rps12 intergenic spacer region. All nucleotide sequence data were recorded in the GenBank sequence database, National Center for Biotechnology Information (NCBI), with the accession numbers KX619478, KX619479, KX619480 and KX619481 respectively. DNA sequencing data were analyzed using the Geneious DNA sequencing analysis software (version 8.1.8, Biomatters Ltd, New Zealand). DNA sequences were compared to published sequences available from Genbank using the Basic Local Alignment Search Tool (BLAST). All analysis sequences were matched with *Gynostemma longipes* with pairwise identity 99.9% (ITS1–5.8S-ITS2 region), 99.6% (rbcL gene), 99.8% (rpl20-rps12 intergenic spacer region) and 99.9% (matK gene). This additional genetic information supported morphological authentication of *Gynostemma longipes* (Fig. 1).

### Extraction and isolation

The aerial part of *G. longipes* (1.5 kg) was powdered, sonicated with 70% EtOH (3 × 3 L), filtered and the solvent was evaporated in vacuo. The crude extract obtained (262 g) was suspended in water, absorbed onto Sephabeads SP70 resin and washed with water, 50% EtOH, 95% EtOH, 100% EtOH and acetone in a sequential elution process. The 95% EtOH fraction (75 g) was subjected to silica gel column chromatography (10 × 35 cm; 63–200 *μ*m particle size) using a gradient of EtOAc/MeOH/H_2_O (from 20/1/0.1 to 4/1/0.1) to give 9 sub-fractions (C1-C9) based on the thin-layer chromatography profile. Compound **1** (50.5 g) was obtained by direct crystallization from sub-fraction C7. Sub-fraction 8 was chromatographed on C18- reversed phase silica gel (RP-C18) column chromatography eluting with MeOH/H_2_O (v/v, from 1/1 to 1/0) and HPLC (Optima Pak C_18_, MeCN/H_2_O (v/v, 2/3), flow rate 2 mL/min) to yield compounds **2** (12.5 mg) and **3** (21.3 mg). Sub-fraction C9 was chromatographed on RP-C18 column chromatography [MeOH/H_2_O (v/v, from 1/1 to 1/0)] to yield 8 subfractions C9–1 to C9-8. Fractions C9-3 by preparative HPLC (Optima Pak C_18_, MeCN/H_2_O (v/v, 3/6), flow rate 2 mL/min) gave compounds **4** (20.4 mg) and **5** (10.5 mg). Fraction C9-4 followed by HPLC (Optima Pak C_18_, MeCN/H_2_O (v/v, 2/3), flow rate 2 ml/min) led to the isolation of compounds **6** (22.2 mg) and **7** (17.1 mg). The 100% EtOH fraction was applied sequentially to Sephadex LH-20 using 100% MeOH and HPLC (Optima Pak C_18_, MeCN/H_2_O (v/v, 7/3), flow rate 2 mL/min) to yield compounds **8** (18.5 mg) and **9** (16.0 mg).

#### Compound **1**

white crystal; ${[\alpha ]}_{{\rm{D}}}^{25}$ -32.0 (*c* 0.1, MeOH); UV (MeOH) λ_max_ (log ε) 244 nm (3.8); IR (KBr) *v*_max_ 3404, 2973, 2938, 1697, 1616, 1073, 1055, 1010 cm^–1^; ^1^H and ^13^C NMR data, Tables 1, 2; HRESIMS *m*/*z* 925.5157 [M−H]^−^ (calcd for C_48_H_77_O_17_ 925.5166).

#### Compound **2**

white, amorphous powder; ${[\alpha ]}_{{\rm{D}}}^{25}$ -37.5 (*c* 0.1, MeOH); UV (MeOH); λ_max_ (log ε) 244 nm (3.8); IR (KBr) *v*_max_ 3400, 2970, 2938, 1698, 1617, 1069, 1053, 1008 cm^–1^; ^1^H and ^13^C NMR data, Tables 1, 2; HRESIMS *m*/*z* 1087.5682 [M−H]^−^ (calcd for C_54_H_87_O_22_ 1087.5694).

#### Compound **3**

white, amorphous powder; ${[\alpha ]}_{{\rm{D}}}^{25}$ -35.2 (*c* 0.1, MeOH); UV (MeOH) λ_max_ (log ε) 244 nm (3.8); IR (KBr) *v*_max_ 3402, 2971, 2938, 1702, 1616, 1060, 1054, 1010 cm^–1^; ^1^H and ^13^C NMR data, Tables 1, 2 HRESIMS *m*/*z* 967.5276 [M−H]^−^ (calcd for C_50_H_79_O_18_ 967.5272).

#### Compound **5**

white, amorphous powder; ${[\alpha ]}_{{\rm{D}}}^{25}$ -33.0 (*c* 0.1, MeOH); UV (MeOH) λ_max_ (log ε) 244 nm (3.7); IR (KBr) *v*_max_ 3403, 2972, 2940, 1702, 1616, 1060, 1054, 1010 cm^–1^; ^1^H and ^13^C NMR data, Tables 1, 2; HRESIMS *m*/*z* 779.4597 [M−H]^−^ (calcd for C_42_H_67_O_13_ 779.4587).

#### Compound **6**

white, amorphous powder; ${[\alpha ]}_{{\rm{D}}}^{25}$ -33.0 (*c* 0.1, MeOH); UV (MeOH) λ_max_ (log ε) 244 nm (3.9); IR (KBr) *v*_max_ 3403, 2972, 2940, 1702, 1616, 1060, 1010 cm^–1^; ^1^H and ^13^C NMR data, Tables 1, 2; HRESIMS *m*/*z* 471.3393 [M−H]^−^ (calcd for C_30_H_47_O_4_ 471.3480).

#### Compound **7**

white, amorphous powder, ${[\alpha ]}_{{\rm{D}}}^{25}$ -38.0 (*c* 0.1, MeOH); UV (MeOH) λ_max_ (log ε) 244 nm (3.8); IR (KBr) *v*_max_ 3403, 2972, 2940, 1702, 1616, 1060, 1054, 1010 cm^–1^; ^1^H and ^13^C NMR data, Tables 1, 2; HRESIMS *m*/*z* 471.3452 [M + H]^+^ (calcd for C_30_H_47_O_4_ 471.3469).

#### Compound **8**

white, amorphous powder, ${[\alpha ]}_{{\rm{D}}}^{25}$ -32.5 (*c* 0.1, MeOH); IR (KBr) *v*_max_ 3403, 2972, 2940, 1702, 1616, 1060, 1054, 1010 cm^–1^; ^1^H and ^13^C NMR data, Tables 1, 2; HRESIMS *m/z* 943.5374 [M-H]^−^ (calcd for C_48_H_79_O_18_ 943.5380).

#### Compound **9**

White amorphous powder, ${[\alpha ]}_{{\rm{D}}}^{25}$ -33.0 (*c* 0.1, MeOH); UV (MeOH) λ_max_ (log ε) 275 nm (3.6); IR (KBr) *v*_max_ 3403, 2972, 2940, 1702, 1616, 1060, 1054, 1010 cm^–1^; ^1^H and ^13^C NMR data: Tables 1, 2; HRESIMS data at *m*/*z* 907.5066 [M−H]^−^ (calcd for C_48_H_75_O_16_ 907.5061).

### Acid hydrolysis

Compounds **1** (20 mg) was hydrolyzed with 1 N HCl (MeOH 70%, 1 mL) at 90 °C for 1 hours. The solution was neutralized by NaOH 10%, dried and suspended in H_2_O and partitioned with EtOAc. The residual H_2_O layer was concentrated and dissolved in pyridine (1.0 mL) and 5.0 mg of l-cysteine methyl ester hydrochloride was added. The mixture was kept for 1 hours at 60 °C, and then 4.4 *μ*L of benzylisothiocyanate was added. This solution was filtered through a 0.2-*µ*m Whatman hydrophilic membrane filter into an HPLC sample vials immediately before LC-MS analysis. Analysis was performed using an Agilent 1200 HPLC system (Agilent Technologies, Palo Alto, CA, USA) with INNO C18 (4.6 × 250 mm inner diameter, 5 *μ*m particle size; Young Jin Bio Chrom Co., Ltd) at a column temperature of 30 °C. Chromatographic condition was carried out using a mobile phase of 27% MeCN/H_2_O isocratic elution at a flow rate 0.6 mL/min in 60 minutes. The sugar derivatives showed the retention time at 21.3 and 16.8 minutes, and were identical to the trimethylsilyl-l-cysteine derivatives of authentic d-glucose and l-rhamnopyranose, respectively.

### Quantitative analysis of compound (1)

The plant extracts were prepared using different extraction solvents with water, 30% EtOH, 50% EtOH, 70% EtOH, 100% EtOH and MeOH, respectively. After the extracts were dried *in vacuo* and lyophilized respectively, the extraction yield of each extract was calculated by the percentage of each extract in 1 g of raw material. For preparation of stock solutions, plant extracts were dissolved in extracted solvents at concentrations of 1 mg/mL. This solution was filtered through a 0.2-*µ*m Whatman hydrophilic membrane filter into an HPLC sample vials immediately before LC-MS analysis. Analysis and validation were performed using an Agilent 1200 HPLC system (Agilent Technologies, Palo Alto, CA, USA) with INNO C18 (4.6 × 250 mm inner diameter, 5 *μ*m particle size; Young Jin Bio Chrom Co., Ltd) at a column temperature of 30 °C. All chromatographic separations were carried out using a mobile phase of 10% to 100% MeCN/H_2_O gradient elution at a flow rate 0.6 mL/min (Supplementary Fig. S2). A calibration curve was drawn using the crystal form of compound **1** at concentrations ranging from 0.025 mg/mL to 0.2 mg/mL. The regression equation is expressed as y = 11333x + 55.578 (correlation coefficient R^2^ = 0.9990), where y and x correspond to the peak area and concentration, respectively. The relative amount of compound in each of the extracts (mg/g of the extract) were calculated using this equation.

### Cell proliferation assay

C2C12 cell lines (mouse skeletal myoblasts) were maintained in growth medium consisting of Dulbecco’s Modified Eagle’s Medium (DMEM) supplemented with 10% fetal bovine serum (FBS) (Gibco, NY, USA), and cells were incubated at 37 °C and 5% CO_2_. The cell proliferation assay was carried out using the (3-(4,5-dimethyl-2-thiazolyl)-2,5-diphenyl-2H-tetrazolium bromide (MTT) (Sigma-Aldrich, St. Louis, MO, USA) or the WST-1 cell proliferation reagent (Roche, Mannheim, Germany). Briefly, C2C12 cells were seeded onto a 96-well plate at 4,000 cells/well and incubated for 8 hours. The cells were then treated with different concentrations of compounds or fraction dissolved in low-serum medium containing 5% FBS. After 24 hours or 48 hours of incubation, 2 mg/mL MTT solution (20 *μ*L) was added to each well and incubated for 4 hours. Formazan was dissolved in 100 *μ*L of DMSO and then absorbance was measured with an Absorbance Microplate Reader (VersaMax^TM^, Randor, PA, USA) at 550 nm. In addition, 10 *μ*L of the WST-1 cell proliferation reagent was added to each well to evaluate cell viability, and incubated for 2 hours, and absorbance was measured at 440 nm. For the cell counting assay, experiments were performed using 36 mm cell culture dishes. After 48 hours of incubation with the tested compound, cells were harvested and evaluated with a Countess^TM^ automated cell counter (Invitrogen, Eugene, OR, USA). For measuring the cell viability of cancer cell lines, MCF-7 and MDA-MB 231 cells were seeded onto 96-well plate at 4,000 cells/well and incubated for 8 hours. Next, cells were cultured in DMEM containing various compounds. After 2 days of incubation, cell viability was evaluated by the above procedure.

### Western blot analysis

C2C12 myoblasts were seeded into 6-well plates and incubated until a confluence of 70–80%. To prepare C2C12 myotubes, cells were incubated with DMEM supplemented with 2% horse serum (Gibco, NY, USA). The differentiation medium was changed every 2 days until myotubes were formed. For Western blot analysis, myotubes or myoblasts were incubated with various concentrations of compounds or fractions for 30–60 minutes. The cells were washed with cold PBS and lysed using the lysis buffer [50 mM Tris-HCl (pH 7.6), 120 mM NaCl, 1 mM EDTA, 0.5% NP-40, 50 mM NaF] and centrifuged at 12,000 rpm for 20 minutes. The protein concentrations were determined using a protein assay kit (Bio-Rad Laboratories, Inc., CA, USA). Aliquots of lysates were electrophoresed on 8% or 12% SDS-polyacrylamide gels and then transferred electronically to polyvinylidene fluoride (PVDF) membranes (PVDF 0.45 *µ*m, Immobilon-P, USA). Membranes were then incubated with primary antibodies for *p*-AMPK*α* Thr^172^, AMPK*α*, *p*-ACC Ser^79^, ACC (Cell signaling Technology, Inc., Beverly, MA, USA) or mouse monoclonal actin (Abcam, Cambridge, UK). After incubation with secondary antibodies, membranes were detected using an enhanced chemiluminescence Western blot detection kit (Thermo sci., Rockford, IL, USA).

### Immunochemical staining with BrdU antibody

C2C12 myoblasts were grown on sterile glass coverslips and the cells were treated with test samples. After incubation for 8 hours, 50 *µ*M BrdU labeling solution (Sigma-Aldrich, St. Louis, MO, USA) was added to the culture cells followed by incubation at 37 °C for 2 hours. The cells were then washed three times with PBS and fixed with 4% formaldehyde in PBS for 15 minutes at room temperature. After washing three times with PBS, the cells were permeabilized with Triton X-100 buffer (Sigma-Aldrich, St. Louis, MO, USA) for 20 minutes at room temperature. Next, the cells were incubated on ice for 20 minutes with 2 N HCl solution. After removing the acid solution, the cells were incubated with phosphate/citric acid buffer (pH 7.4) for 10 minutes at room temperature. Cells were washed three times with Triton X-100 permeabilization buffer and incubated overnight with anti-BrdU monoclonal antibody (Santa Cruz, CA, USA) diluted 1:50 in PBS (pH 7.4). Cells were continuously incubated with FITC-conjugated goat anti-mouse lgG (Life technologies, Eugene, OR, USA) for 1 hour and then stained with 500 nM DAPI solution (Life technologies, Eugene, OR, USA) for 10 minutes at room temperature. The slides were fixed with a mounting medium for fluorescence detection (Vectashield^®^, Vector Lab, Inc., USA) and images were observed using fluorescence microscopy (Olympus ix70 Fluorescence Microscope, Olympus Corporation, Tokyo, Japan).

### Flow cytometry analysis for BrdU and PI staining

For flow cytometry with anti-BrdU antibody staining, C2C12 myoblasts were maintained in 36-mm culture dishes. After treatment with different concentrations of the test compounds, the cells were incubated for 8 hours. Next, 50 *µ*M BrdU labeling solution was added to the culture medium and incubated for 2 hours. After removing BrdU medium, the cells were washed with PBS and harvested by trypsin solution. Cells were permeabilized using precooled 70% ethanol solution and further incubated at 4 °C for 1 hour. After centrifugation at 1000 rpm for 2 minutes, the pellet cells were continually added to a 2 N HCl solution containing 0.5% Triton X-100 reagent and incubated at room temperature for 30 minutes. Cells were then washed with PBS containing 1% BSA (Sigma-Aldrich, St. Louis, MO, USA) and resuspended in PBS containing 0.5% Tween 20 and 1% BSA. Cells were incubated with anti-BrdU monoclonal antibody (Santa Cruz, CA, USA) for 4 hours at room temperature. Cells were resuspended in PBS containing 20 *µ*g/mL propidium iodide (PI) (Merck Millipore, MA, USA) and kept in the dark for 30 minutes. Then, double-stained cells were analyzed using a flow cytometer (BD, FACSCalibur, San Jose, CA, USA) and at least 10,000 cells were counted for each analysis.

### Flow cytometry of cell cycle status

C2C12 myoblasts were maintained in 36-mm culture dishes and cells were treated with the test compounds. After 12 or 24 hours incubation, cells were harvested using trypsinization and washed twice with pre-cooled PBS. Cells were then fixed with pre-cooled 70% ethanol solution and incubated overnight at 4 °C. After washing with PBS, cells were stained sequentially with 20 *µ*g/mL of PI in PBS solution and incubated at room temperature for 30 minutes. Cellular DNA content was analyzed using a flow cytometer (BD, FACSCalibur, San Jose, CA, USA). At least 10,000 cells were counted for each assay. At each phase of the cell cycle, cell distribution was displayed using a histogram.

### Glucose uptake assay

C2C12 myoblasts were seeded into black 96-well plates at 10^4^ cells/well using glucose-free medium containing 10% FBS. After 1 day of incubation, cells were treated with test samples or insulin (as a positive control) in the presence or absence of 2-NBDG (Invitrogen, Eugene, OR, USA). The cells were continually incubated for 1 hour at 37 °C and they were then washed with cold PBS. The fluorescence intensity was measured with excitation/emission at 450/535 nm using a fluorescence microplate reader (Spectra Max Gemini XPS, Molecular Devices, San Jose, CA, USA).

### Measurement of ATP level

Intracellular ATP production was evaluated using ATP bioluminescence determination assay kit (Invitrogen, Eugene, OR, USA). C2C12 myoblasts were grown on 24-well plates overnight and the cells were then exposed to test samples from 0 to 70 minutes. After that, the cells were lysed using lysis buffer (100 *µ*L). The cell lysates and ATP standard dilutions (20 *µ*L) were transferred to white 96-well plates and then luciferase mixture (80 *µ*L) was added to each well. ATP levels were measured using a luminescence microplate reader (Spectra Max L, Molecular Devices, San Jose, CA, USA).

### Statistical analysis

All data were expressed as the mean ± SD of 2–3 independent experiments. The difference between group mean values was calculated by analysis of variance (ANOVA) followed by Duncan’s or Tukey’s post hoc test as appropriate, conducting in SPSS Statistics 23 (SPSS, Inc., Chicago, IL, USA). Statistical significance was accepted at *p < 0.05, and **p < 0.01, ***p < 0.001.

## Supplementary information

Supporting information

## Data Availability

All data generated or analysed for this study are included in this published paper (and its Supplementary Information files).
