# Supplementary material for: 12,23-Dione dammarane triterpenes from Gynostemma longipes and their muscle cell proliferation activities via activation of the AMPK pathway
Source: Sci Rep. 2019 Feb 4;9:1186. doi: 10.1038/s41598-018-37808-9 (PMC6361897; doi:10.1038/s41598-018-37808-9)
Supplement: Supplementary file 1 — Supporting information [file 41598_2018_37808_MOESM1_ESM.docx]

**SUPPLEMENTARY DATA**

**12,23-Dione dammarane triterpenes from *Gynostemma longipes* and their muscle cell proliferation activities via activation of the AMPK pathway**

Thi Kim Quy Ha^1,+^, Ha Thanh Tung Pham^1,+^, Hyo Moon Cho^1^, Van On Tran^2^, Jun-Li Yang^3^, Da-Woon Jung^4^, Darren R. Williams^4^ & Won Keun Oh^1,*^

^1^Korea Bioactive Natural Material Bank, Research Institute of Pharmaceutical Sciences, College of Pharmacy, Seoul National University, Seoul 08826, Republic of Korea

^2^Hanoi University of Pharmacy, Hanoi, Vietnam

^3^Key Laboratory of Chemistry of Northwestern Plant Resources of CAS and Key Laboratory for Natural Medicine of Gansu Province, Lanzhou Institute of Chemical Physics, Chinese Academy of Sciences, Lanzhou 730000, P. R. China

^4^New Drug Targets Laboratory, School of Life Sciences, Gwangju Institute of Science and Technology, 1 Oryong-Dong, Buk-Gu, Gwangju 61005, Republic of Korea

**^+^**These authors contributed equally to this work.

^*^ To whom correspondence should be addressed. Tel & Fax: +82-02-880-7872. E-mail: [wkoh1@snu.ac.kr](mailto:wkoh1@snu.ac.kr).

**TABLE OF CONTENTS**

**Figure S1.** UV and HPLC spectra of 9 isolated compounds 4

**Figure S2.** HPLC chromatograms of standard compound **1** 5

**Figure S3.** Structure and HRESIMS of compound **1** 6

**Figure S4.** ^1^H NMR spectrum (Pyridine-d*_5_*, 500 MHz) of compound **1** 7

**Figure S5.** ^13^C NMR spectrum (Pyridine-d*_5_*, 125 MHz) of compound **1** 7

**Figure S6.** HSQC (H→C) spectrum (Pyridine-d*_5_*, 500 MHz) of compound **1** 8

**Figure S7.** HMBC (H→C) spectrum (Pyridine-d*_5_*, 500 MHz) of compound **1** 8

**Figure S8.** COSY (H→H) spectrum (Pyridine-d*_5_*, 600 MHz) of compound **1** 9

**Figure S9.** NOESY (H→H) spectrum (Pyridine-d*_5_*, 600 MHz) of compound **1** 9

**Figure S10.** Structure and HRESIMS of compound **2** 10

**Figure S11.** ^1^H NMR spectrum (Pyridine-d*_5_*, 600 MHz) of compound **2** 11

**Figure S12.** ^13^C NMR spectrum (Pyridine-d*_5_*, 150 MHz) of compound **2** 11

**Figure S13.** HSQC (H→C) spectrum (Pyridine-d*_5_*, 600 MHz) of compound **2** 12

**Figure S14.** HMBC (H→C) spectrum (Pyridine-d*_5_*, 600 MHz) of compound **2** 12

**Figure S15.** COSY (H→H) spectrum (Pyridine-d*_5_*, 600 MHz) of compound **2** 13

**Figure S16.** Structure and HRESIMS of compound **3** 14

**Figure S17.** ^1^H NMR spectrum (Pyridine-d*_5_*, 600 MHz) of compound **3** 15

**Figure S18.** ^13^C NMR spectrum (Pyridine-d*_5_*, 150 MHz) of compound **3** 15

**Figure S19.** HSQC (H→C) spectrum (Pyridine-d*_5_*, 600 MHz) of compound **3** 16

**Figure S20.** HMBC (H→C) spectrum (Pyridine-d*_5_*, 600 MHz) of compound **3** 16

**Figure S21.** Structure and HRESIMS of compound **5** 17

**Figure S22.** ^1^H NMR spectrum (Pyridine-d*_5_*, 500 MHz) of compound **5** 18

**Figure S23.** ^13^C NMR spectrum (Pyridine-d*_5_*, 125 MHz) of compound **5** 18

**Figure S24.** HSQC (H→C) spectrum (Pyridine-d*_5_*, 500 MHz) of compound **5** 19

**Figure S25.** HMBC (H→C) spectrum (Pyridine-d*_5_*, 500 MHz) of compound **5** 19

**Figure S26.** COSY (H→H) spectrum (Pyridine-d*_5_*, 500 MHz) of compound **5** 20

**Figure S27.** Structure and HRESIMS of compound **6** 21

**Figure S28.** ^1^H NMR spectrum (Pyridine-d*_5_*, 600 MHz) of compound **6** 22

**Figure S29.** ^13^C NMR spectrum (Pyridine-d*_5_*, 150 MHz) of compound **6** 22

**Figure S30.** HSQC (H→C) spectrum (Pyridine-d*_5_*, 600 MHz) of compound **6** 23

**Figure S31.** HMBC (H→C) spectrum (Pyridine-d*_5_*, 600 MHz) of compound **6** 23

**Figure S32.** Structure and HRESIMS of compound **7** 24

**Figure S33.** ^1^H NMR spectrum (Pyridine-d*_5_*, 500 MHz) of compound **7** 25

**Figure S34.** ^13^C NMR spectrum (Pyridine-d*_5_*, 125 MHz) of compound **7** 25

**Figure S35.** HSQC (H→C) spectrum (Pyridine-d*_5_*, 500 MHz) of compound **7** 26

**Figure S36.** HMBC (H→C) spectrum (Pyridine-d*_5_,* 500 MHz) of compound **7** 26

**Figure S37.** Structure and HRESIMS of compound **8** 27

**Figure S38.** ^1^H NMR spectrum (Pyridine-d*_5_*, 500 MHz) of compound **8** 28

**Figure S39.** ^13^C NMR spectrum (Pyridine-d*_5_*, 125 MHz) of compound **8** 28

**Figure S40.** HSQC (H→C) spectrum (Pyridine-d*_5_*, 500 MHz) of compound **8** 29

**Figure S41.** HMBC (H→C) spectrum (Pyridine-d*_5_,* 500 MHz) of compound **8** 29

**Figure S42.** Structure and HRESIMS of compound **9** 30

**Figure S43.** ^1^H NMR spectrum (Pyridine-d*_5_*, 600 MHz) of compound **9** 31

**Figure S44.** ^13^C NMR spectrum (Pyridine-d*_5_*, 150 MHz) of compound **9** 31

**Figure S45.** HSQC (H→C) spectrum (Pyridine-d*_5_*, 600 MHz) of compound **9** 32

**Figure S46.** HMBC (H→C) spectrum (Pyridine-d*_5_,* 600 MHz) of compound **9** 32

**Figure S47.** Effect of EtOH 95% fraction from *G. longipes* on C2C12 myoblast cells proliferation using the counting cell method. 33

**Figure S48.** Activation effect of the SP70-EtOH 95% fraction from *G. longipes* on the phosphorylation (Thr172) of AMPK*α*; original uncropped blots. 34

**Figure S49.** The effects of co-treatment compound **C** with the active fraction or compound **1** on *p*-AMPK (Thr172) in C2C12 myoblasts; original uncropped blots. 35

**Figure S49.** The effects of co-treatment compound **C** with the active fraction or compound **1** on *p*-AMPK (Thr172) in C2C12 myoblasts; original uncropped blots. (Continued) 36

**Figure S50.** Effect of compound **1** on the proliferation of cancer cell lines (MCF-7 and MDA-MB 231 cells). 37

**Figure S51.** Stimulation effect of isolated compounds (**1**−**9**) on *p-*AMPK (Thr^172^) and *p-*ACC (Ser^79^) in differentiated mouse C2C12 skeletal myoblasts**.** 38

**Figure S52.** Stimulation effect of isolated compounds (**1**−**9**) on *p-*AMPK (Thr^172^) and *p-*ACC (Ser^79^) in C2C12 myotubes; original uncropped blots. 40

**Figure S53.** Stimulation effects of compounds **1**, **5**, **6**, **7**, and the active fraction on the expression of *p*-AMPK in mouse C2C12 myoblasts, original uncropped blots. 41

**Figure S54.** Time-dependent effect of compound **1** on the *­p*-AMPK protein; original uncropped blots. 42

**Figure S55.** Compound **1** increased the expressions of AMPK*α* protein when C2C12 myoblast cells were incubated with compound 1 at different concentrations for 24 hours. 44

**Figure S56.** Effect of compound **1** on the expressions of AMPK*α* protein; original uncropped blots. 45

**Figure S57.** Effects of the active fraction and compound **1** on DNA synthesis during cell proliferation. 46

**Figure S58.** Effects of co-treatment compound C with the active fraction and compound **1** on DNA synthesis during cell proliferation. 47

**Figure S59.** C2C12 myoblast cells were treated with compound **1** (20 *μ*M) and incubated for 12 hours (A) or 24 hours (B). 48


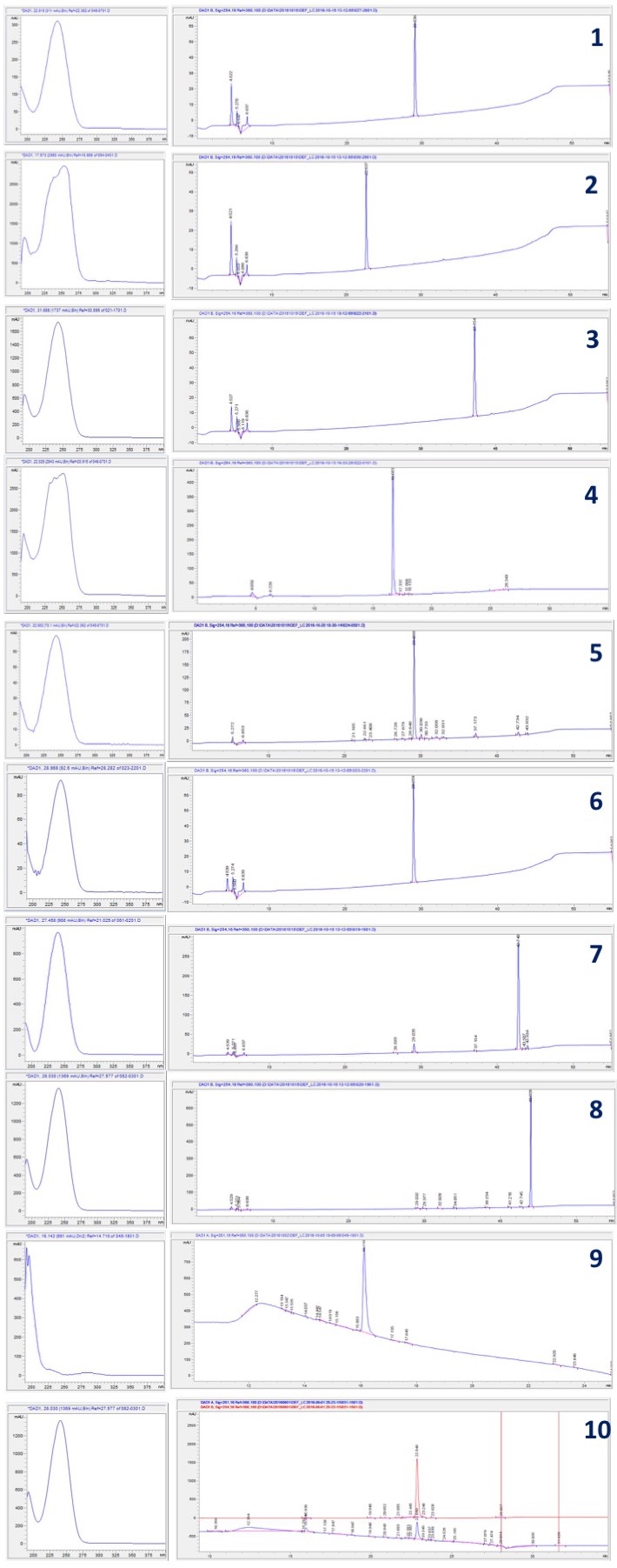


**Figure S1.** UV and HPLC spectra of 9 isolated compounds

**
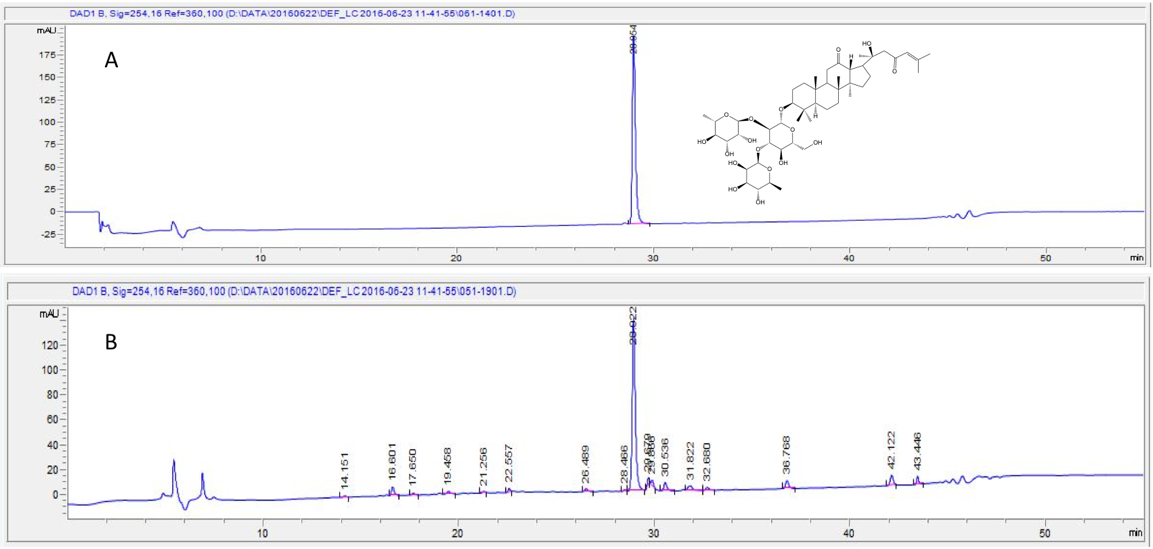
**

**Figure S2.** HPLC chromatograms of compound **1**

**(A)** and EtOH extract 70% **(B)** of *Gynostemma longipes*

COMPOUND **1**

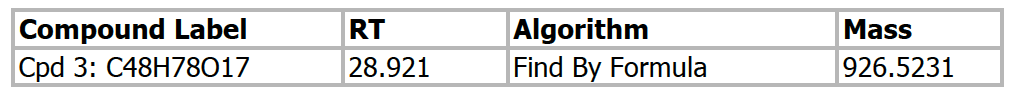


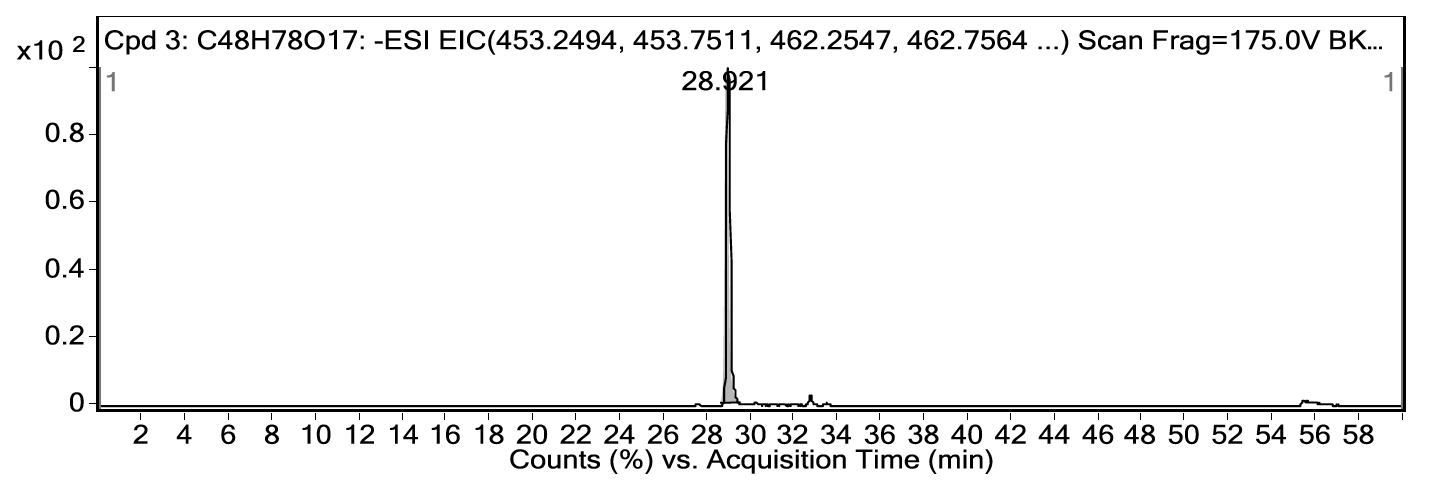


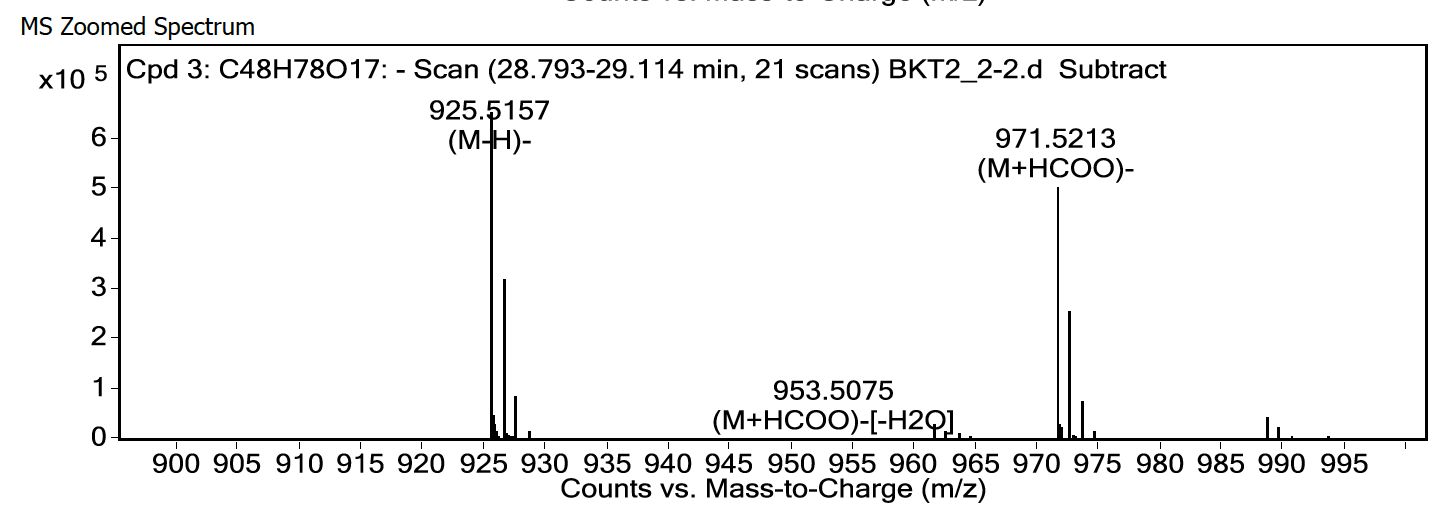


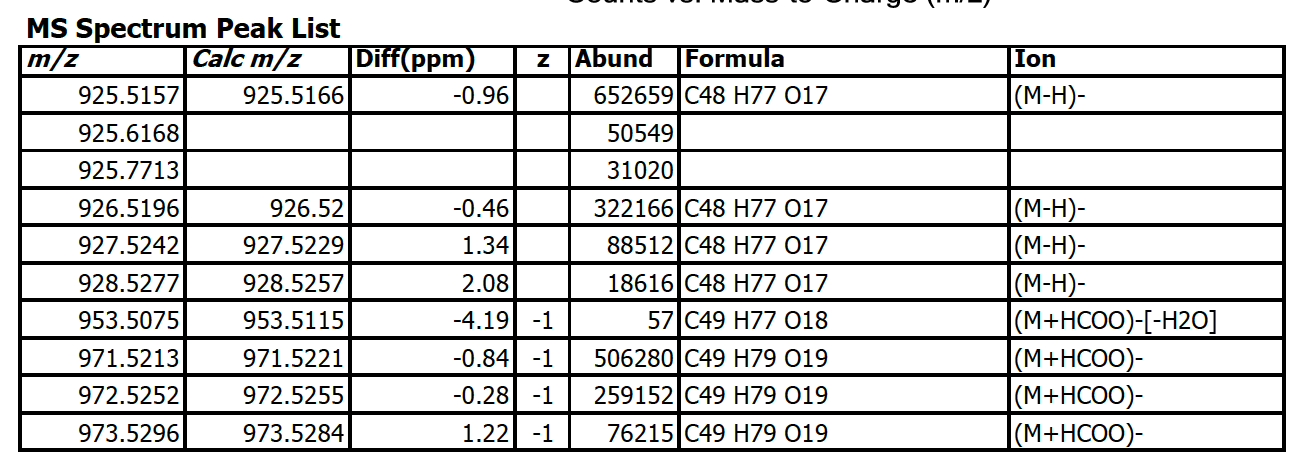


Figure S3. Structure and HRESIMS of compound 1

Figure S4. ^1^H NMR spectrum (Pyridine-d*_5_*, 500 MHz) of compound 1

Figure S5. ^13^C NMR spectrum (Pyridine-d*_5_*, 125 MHz) of compound 1

Figure S6. HSQC (H→C) spectrum (Pyridine-d*_5_*, 500 MHz) of compound 1

Figure S7. HMBC (H→C) spectrum (Pyridine-d*_5_*, 500 MHz) of compound 1

Figure S8. COSY (H→H) spectrum (Pyridine-d*_5_*, 600 MHz) of compound 1

Figure S9. NOESY (H→H) spectrum (Pyridine-d*_5_*, 600 MHz) of compound 1

COMPOUND **2**

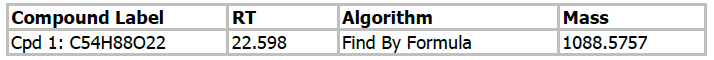


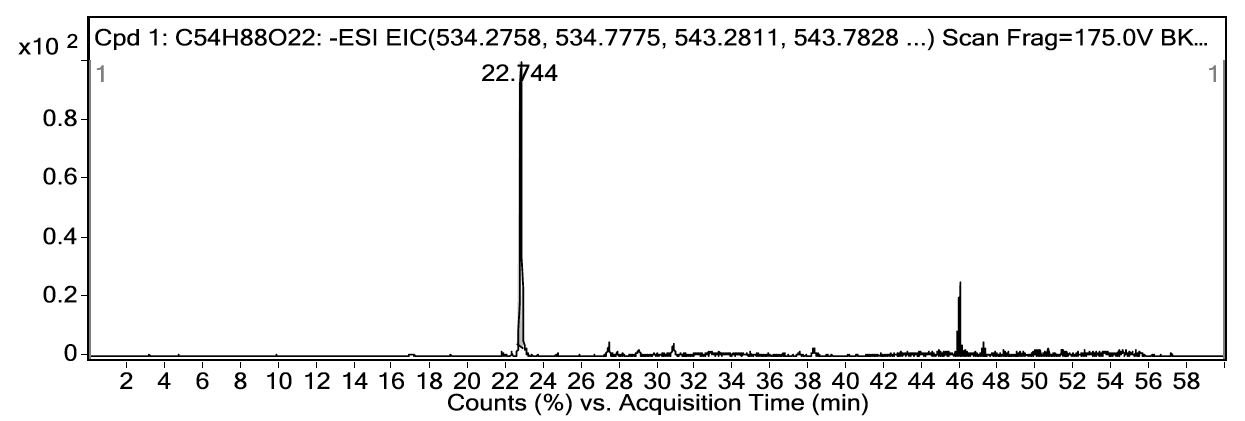


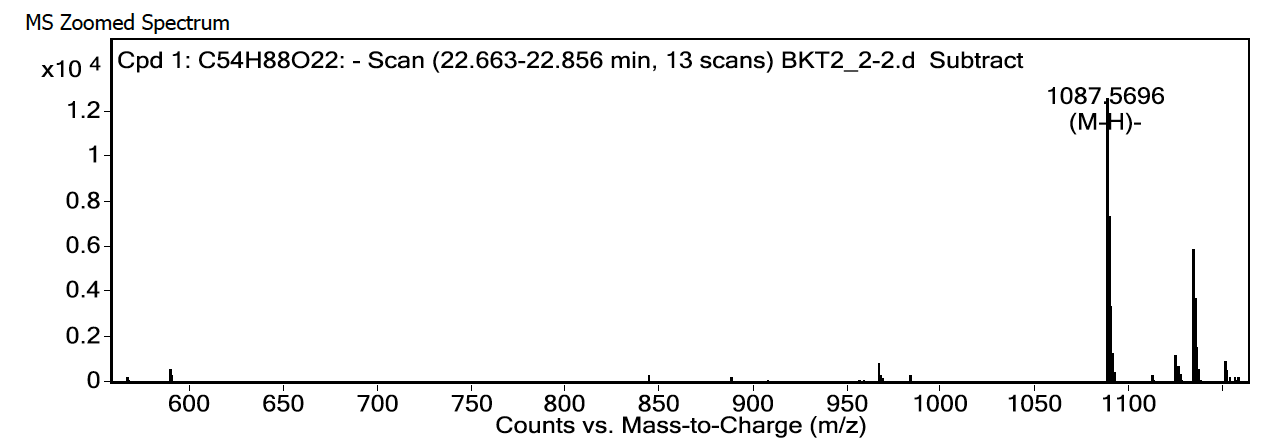


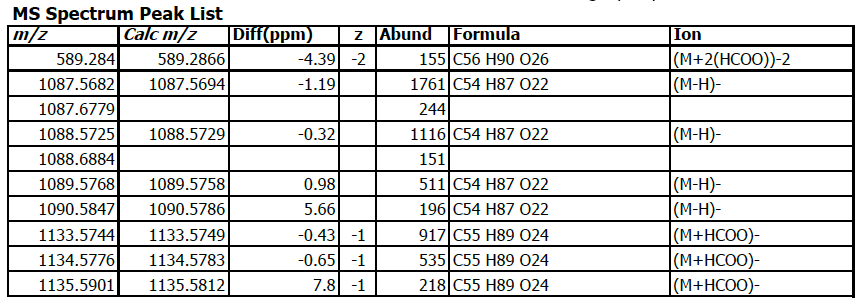


Figure S10. Structure and HRESIMS of compound 2

Figure S11. ^1^H NMR spectrum (Pyridine-d*_5_*, 600 MHz) of compound 2

Figure S12. ^13^C NMR spectrum (Pyridine-d*_5_*, 150 MHz) of compound 2

**
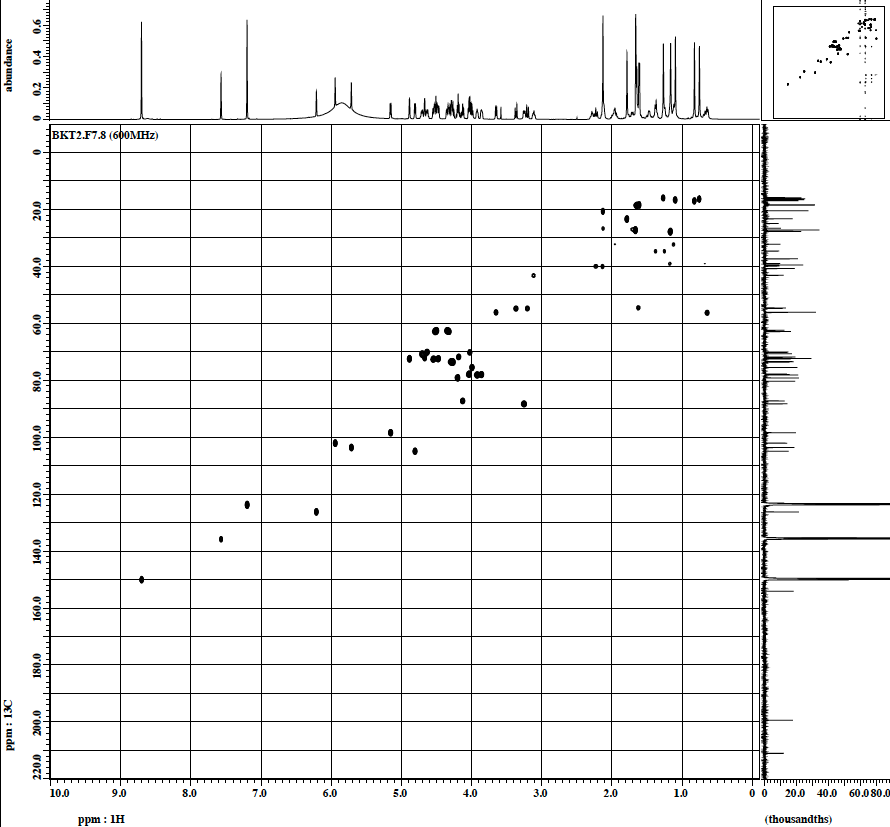
**

Figure S13. HSQC (H→C) spectrum (Pyridine-d*_5_*, 600 MHz) of compound 2

Figure S14. HMBC (H→C) spectrum (Pyridine-d*_5_*, 600 MHz) of compound 2

Figure S15. COSY (H→H) spectrum (Pyridine-d*_5_*, 600 MHz) of compound 2

COMPOUND **3**

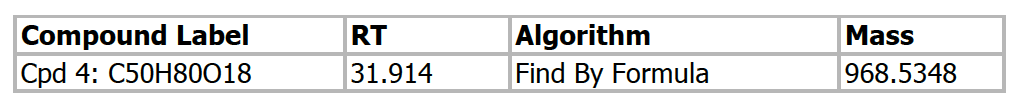


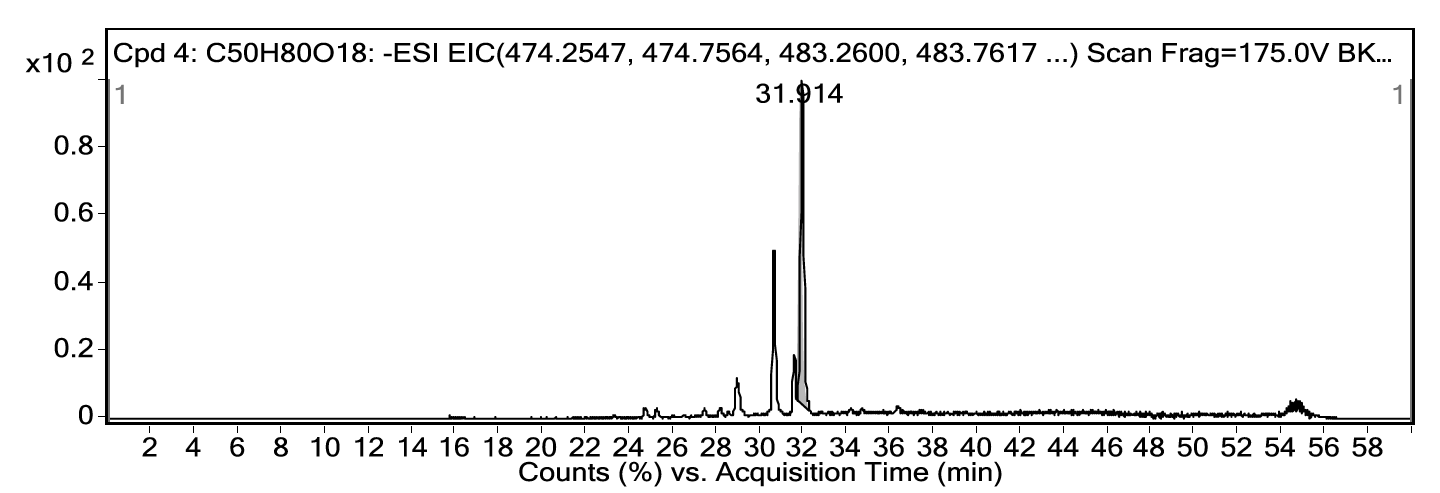


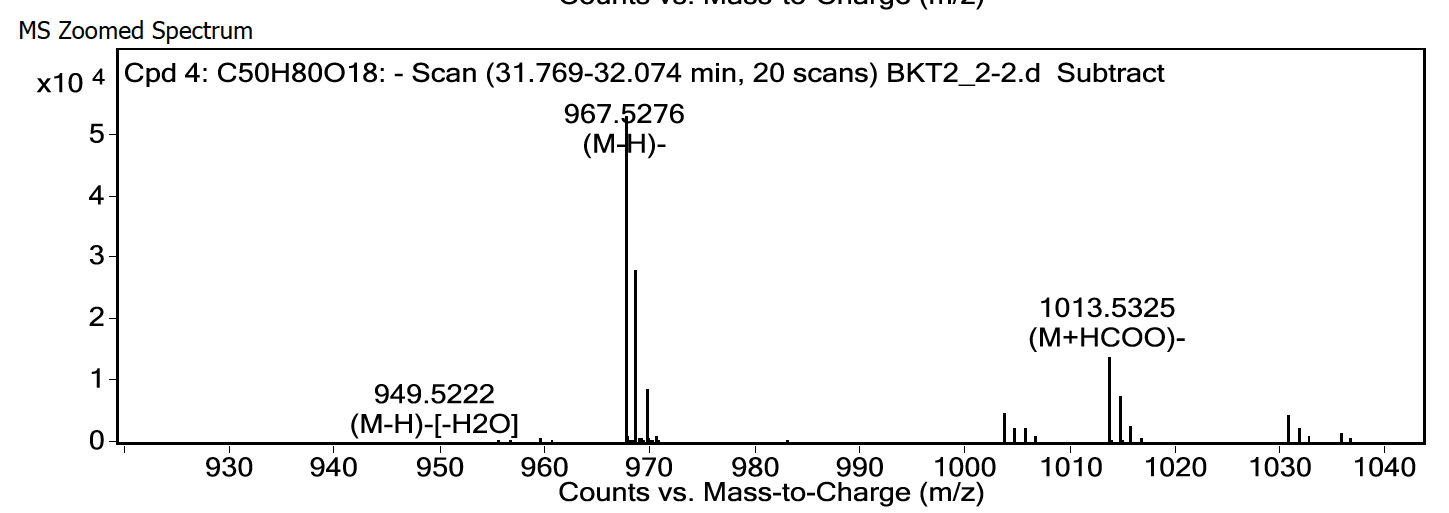


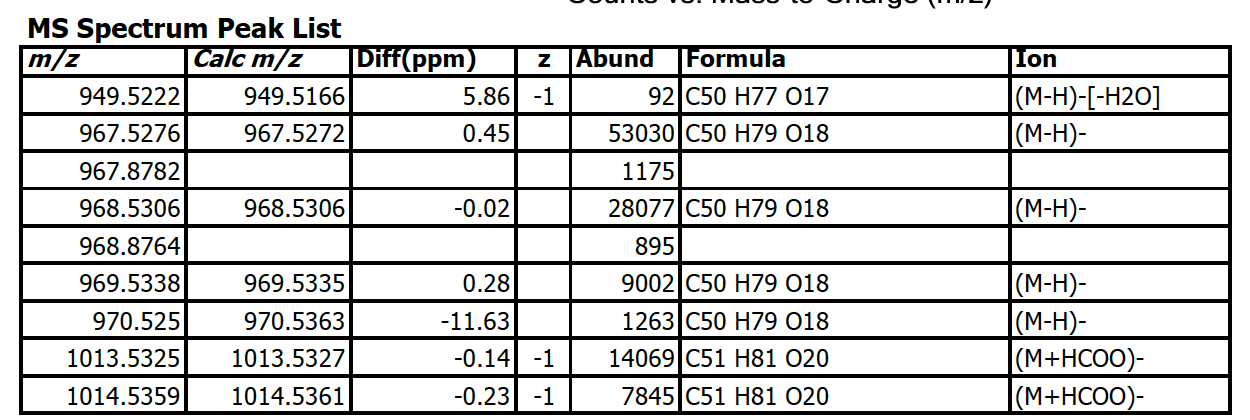


Figure S16. Structure and HRESIMS of compound 3

Figure S17. ^1^H NMR spectrum (Pyridine-d*_5_*, 600 MHz) of compound 3

Figure S18. ^13^C NMR spectrum (Pyridine-d*_5_*, 150 MHz) of compound 3

**
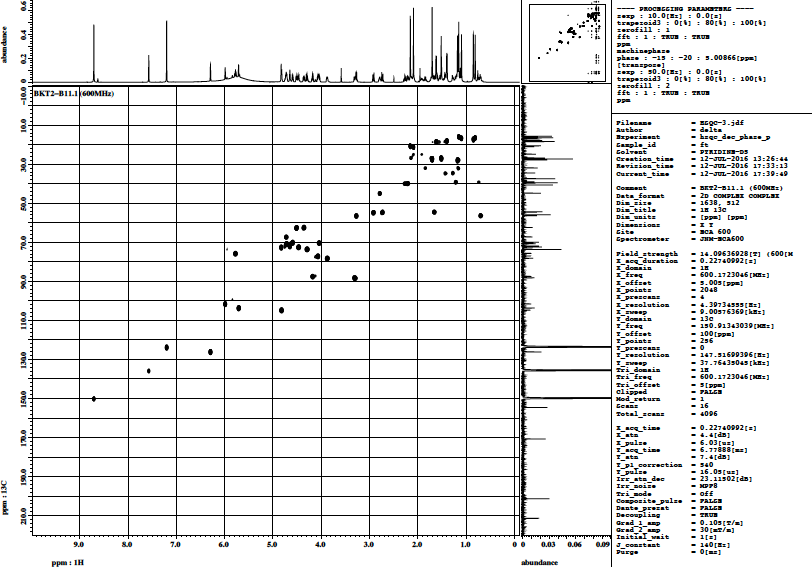
**

Figure S19. HSQC (H→C) spectrum (Pyridine-d*_5_*, 600 MHz) of compound 3

Figure S20. HMBC (H→C) spectrum (Pyridine-d*_5_*, 600 MHz) of compound 3

COMPOUND **5**

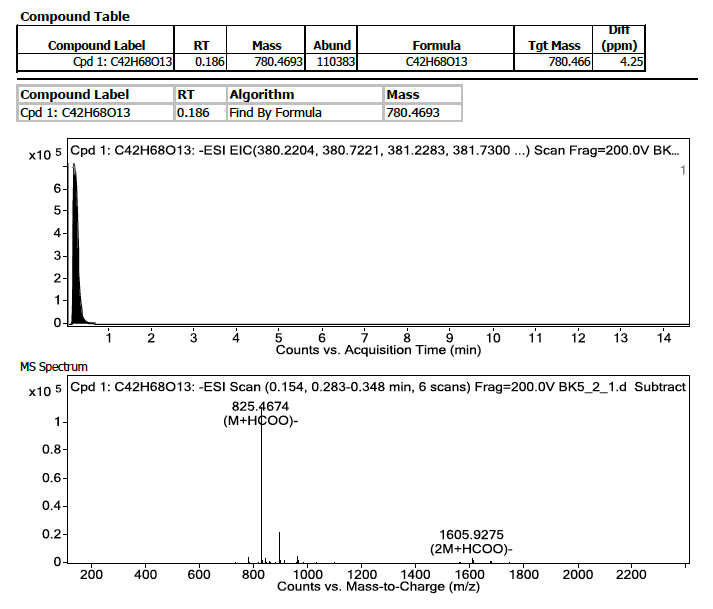


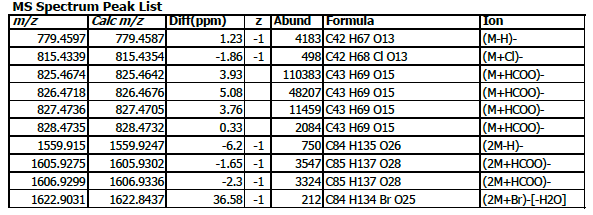


Figure S21. Structure and HRESIMS of compound 5

Figure S22. ^1^H NMR spectrum (Pyridine-d*_5_*, 500 MHz) of compound 5

Figure S23. ^13^C NMR spectrum (Pyridine-d*_5_*, 125 MHz) of compound 5

Figure S24. HSQC (H→C) spectrum (Pyridine-d*_5_*, 500 MHz) of compound 5

Figure S25. HMBC (H→C) spectrum (Pyridine-d*_5_*, 500 MHz) of compound 5

Figure S26. COSY (H→H) spectrum (Pyridine-d*_5_*, 500 MHz) of compound 5

COMPOUND **6**

| ****** |
| --- |
|  |

**
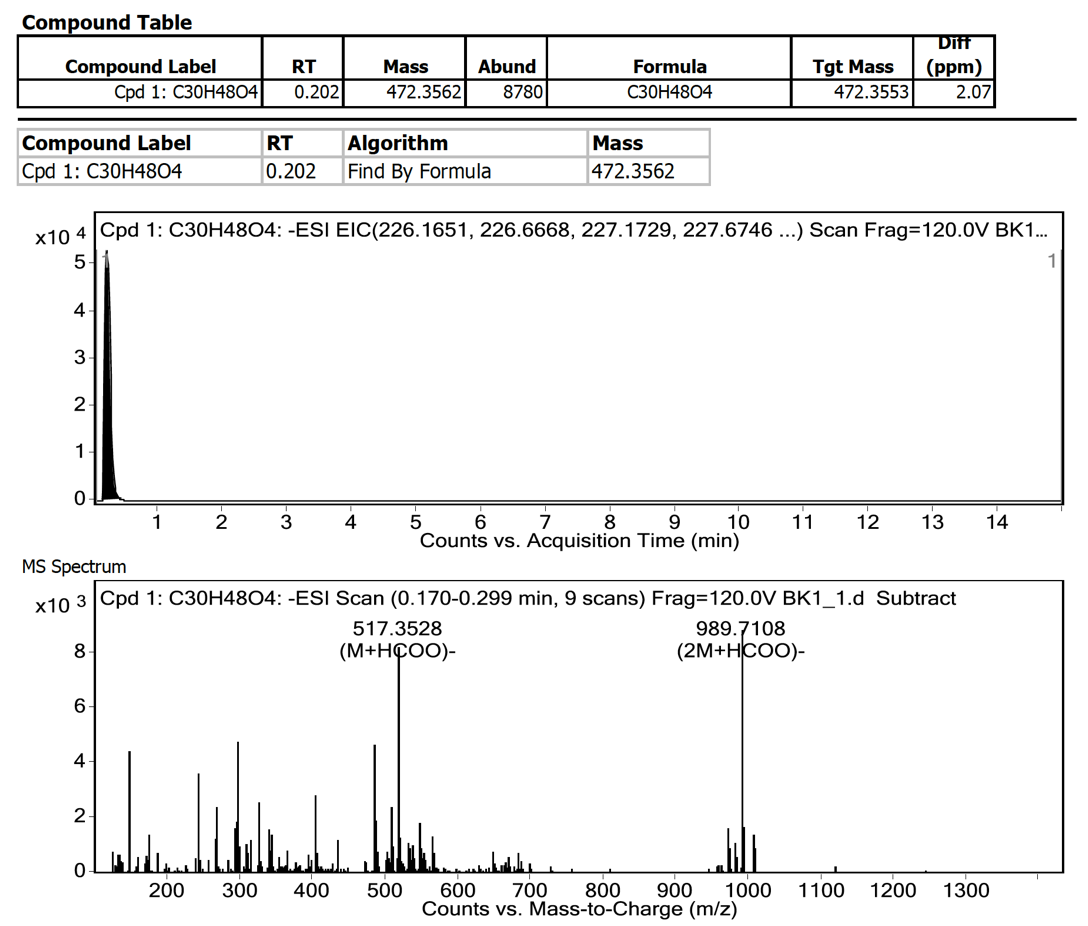
**

**
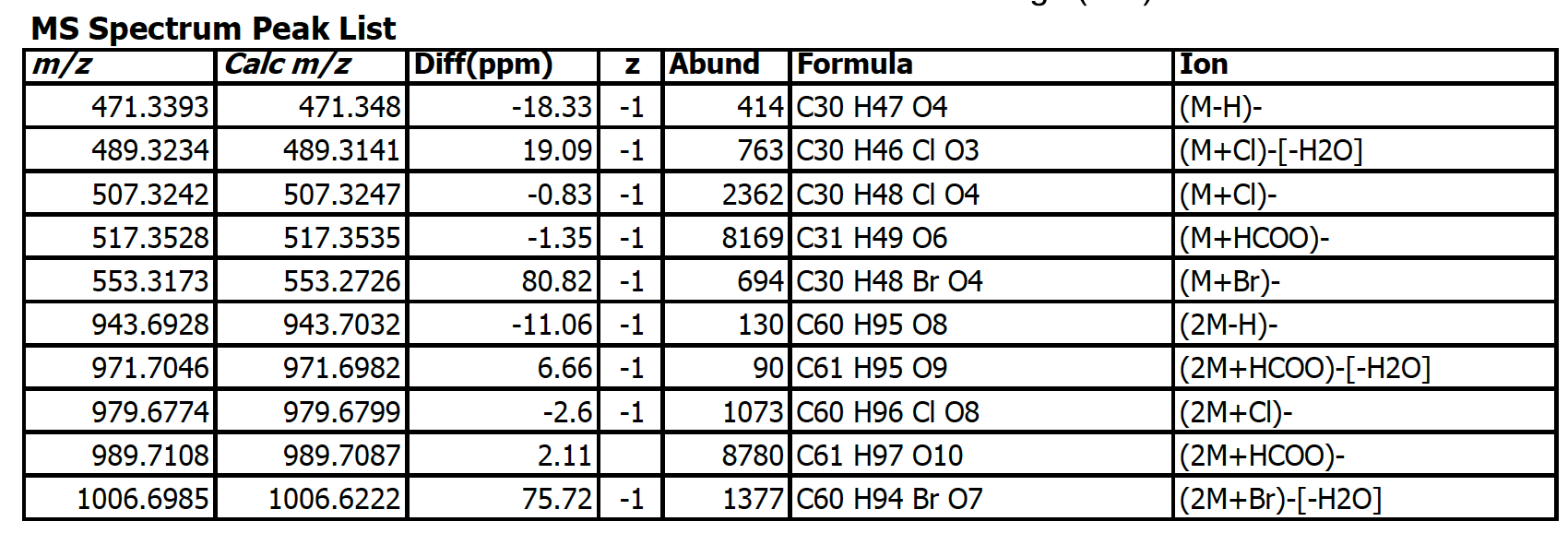
**

Figure S27. Structure and HRESIMS of compound 6

Figure S28. ^1^H NMR spectrum (Pyridine-d*_5_*, 600 MHz) of compound 6

Figure S29. ^13^C NMR spectrum (Pyridine-d*_5_*, 150 MHz) of compound 6


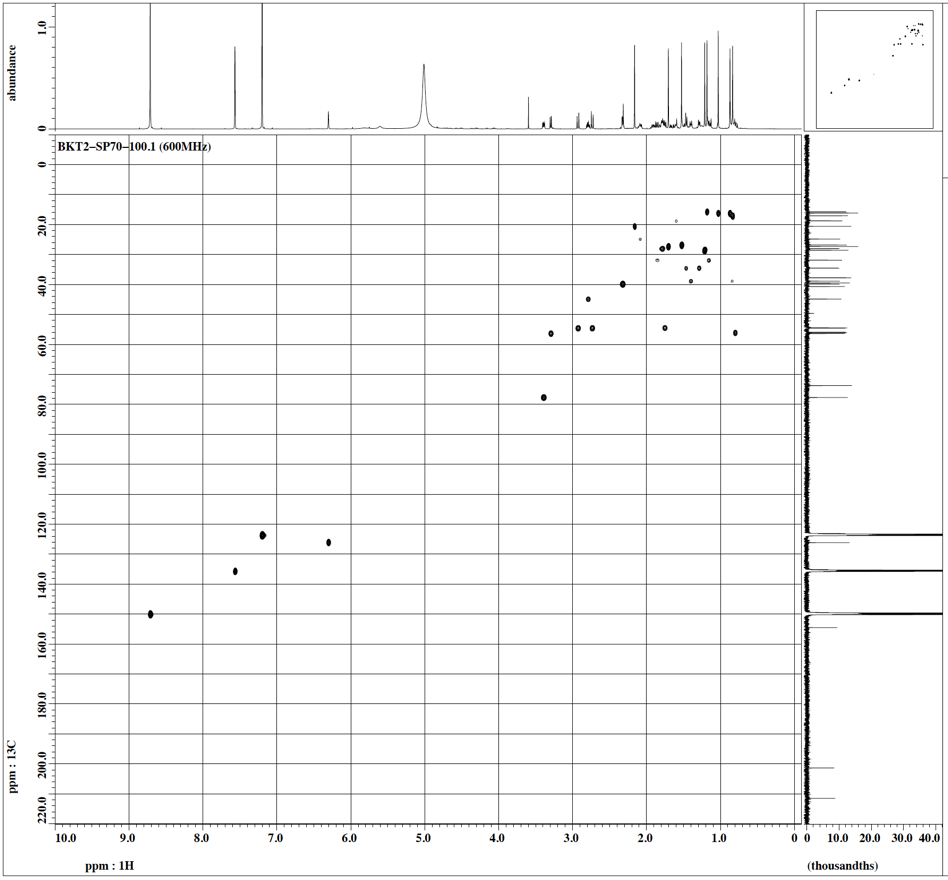


Figure S30. HSQC (H→C) spectrum (Pyridine-d*_5_*, 600 MHz) of compound 6

Figure S31. HMBC (H→C) spectrum (Pyridine-d*_5_*, 600 MHz) of compound 6

COMPOUND **7**

| ****** |
| --- |

**
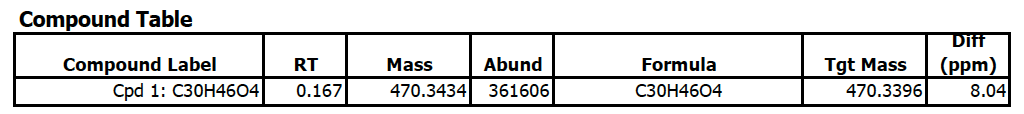
**


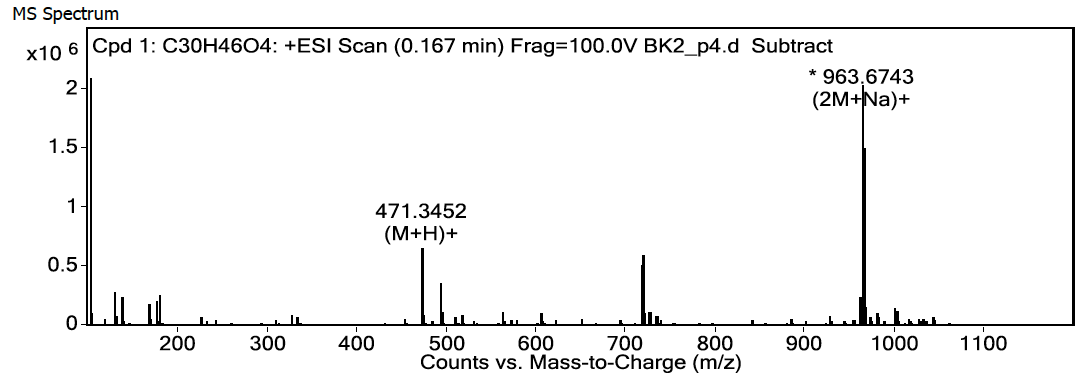


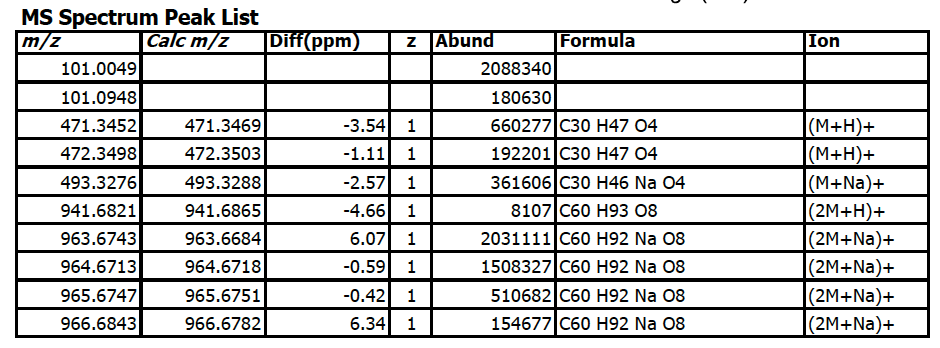


Figure S32. Structure and HRESIMS of compound 7


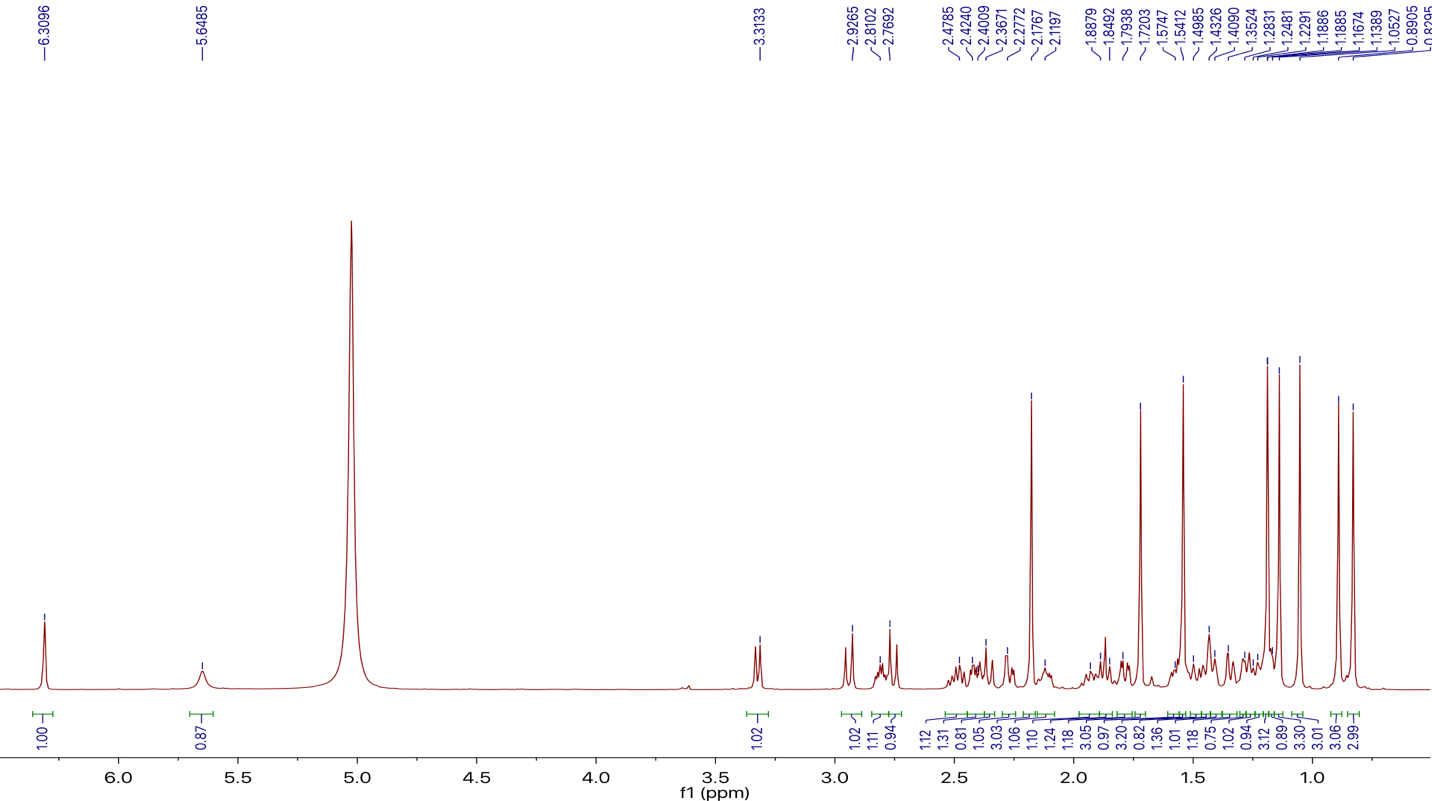


Figure S33. ^1^H NMR spectrum (Pyridine-d*_5_*, 500 MHz) of compound 7

Figure S34. ^13^C NMR spectrum (Pyridine-d*_5_*, 125 MHz) of compound 7


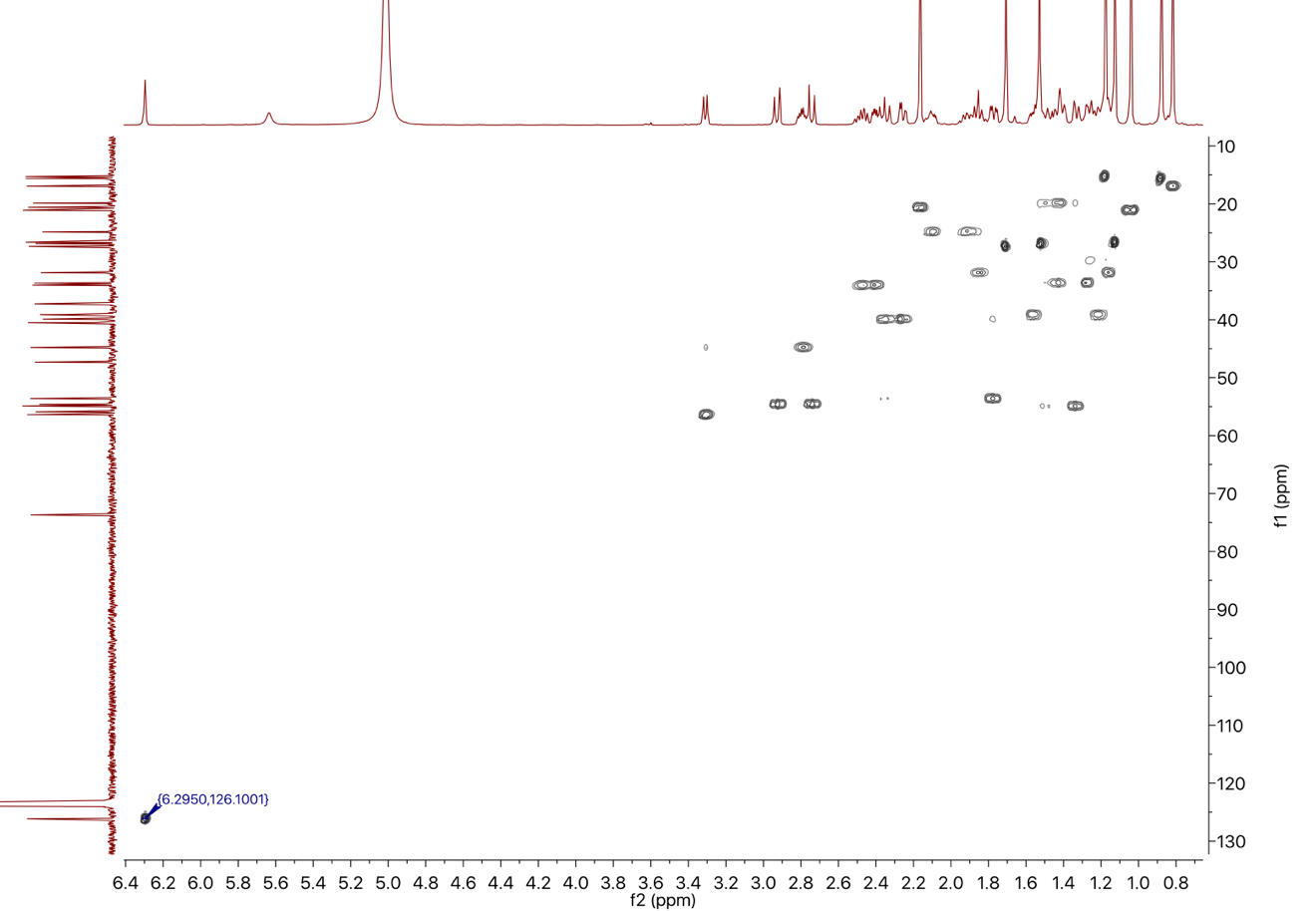


Figure S35. HSQC (H→C) spectrum (Pyridine-d*_5_*, 500 MHz) of compound 7


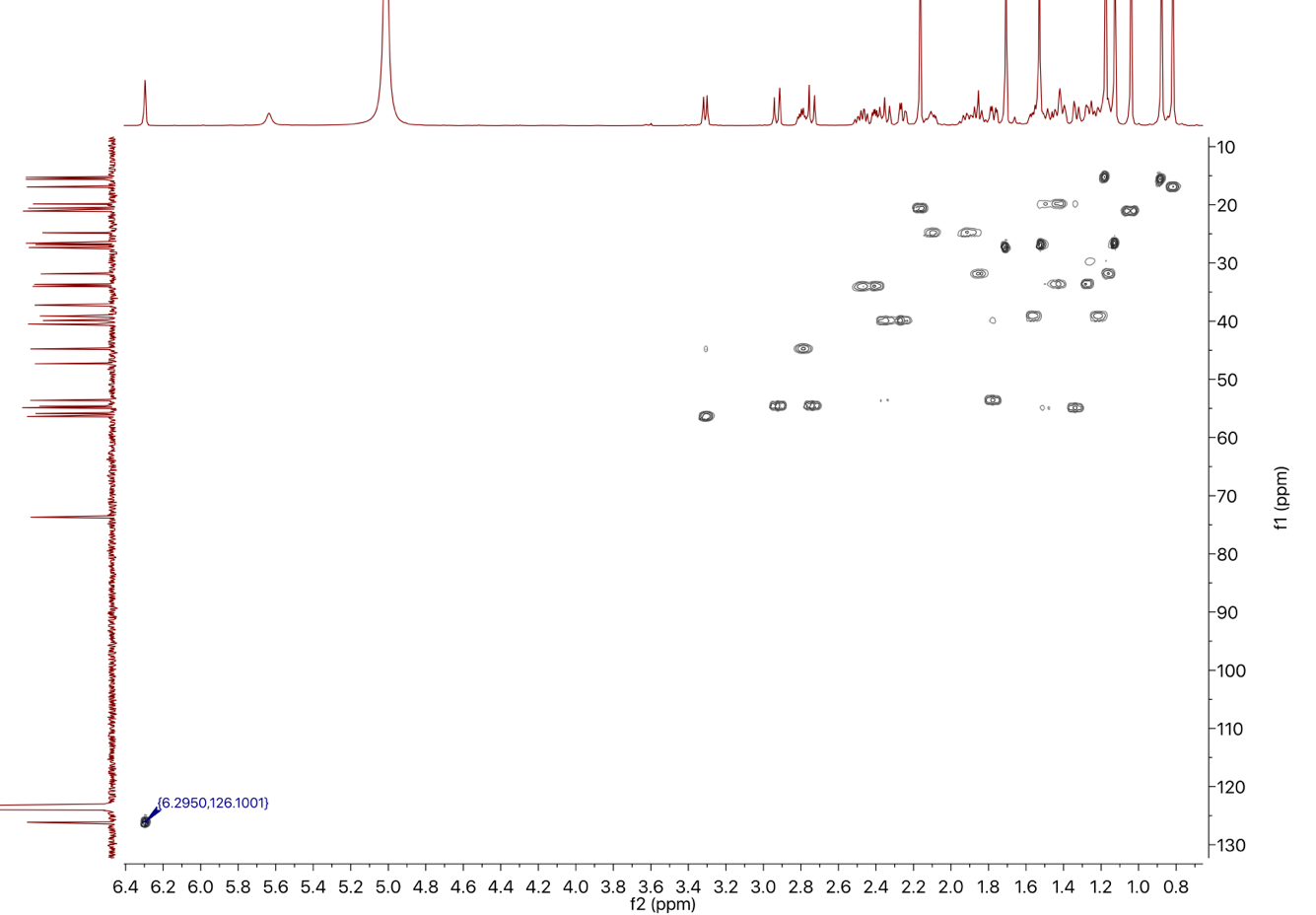


Figure S36. HMBC (H→C) spectrum (Pyridine-d*_5_,* 500 MHz) of compound 7

COMPOUND **8**

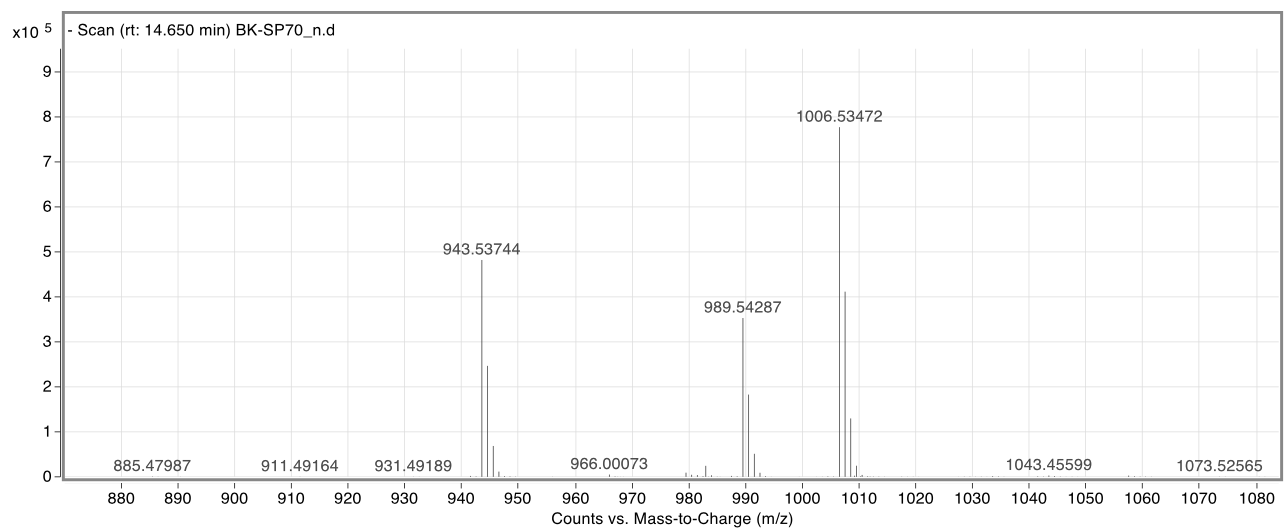


Figure S37. Structure and HRESIMS of compound 8

Figure S38. ^1^H NMR spectrum (Pyridine-d*_5_*, 500 MHz) of compound 8

Figure S39. ^13^C NMR spectrum (Pyridine-d*_5_*, 125 MHz) of compound 8

Figure S40. HSQC (H→C) spectrum (Pyridine-d*_5_*, 500 MHz) of compound 8

Figure S41. HMBC (H→C) spectrum (Pyridine-d*_5_,* 500 MHz) of compound 8

COMPOUND **9**

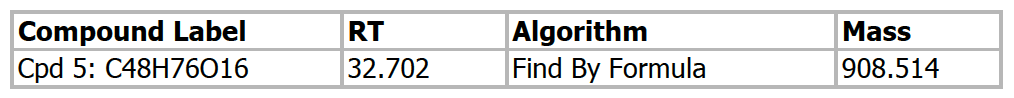


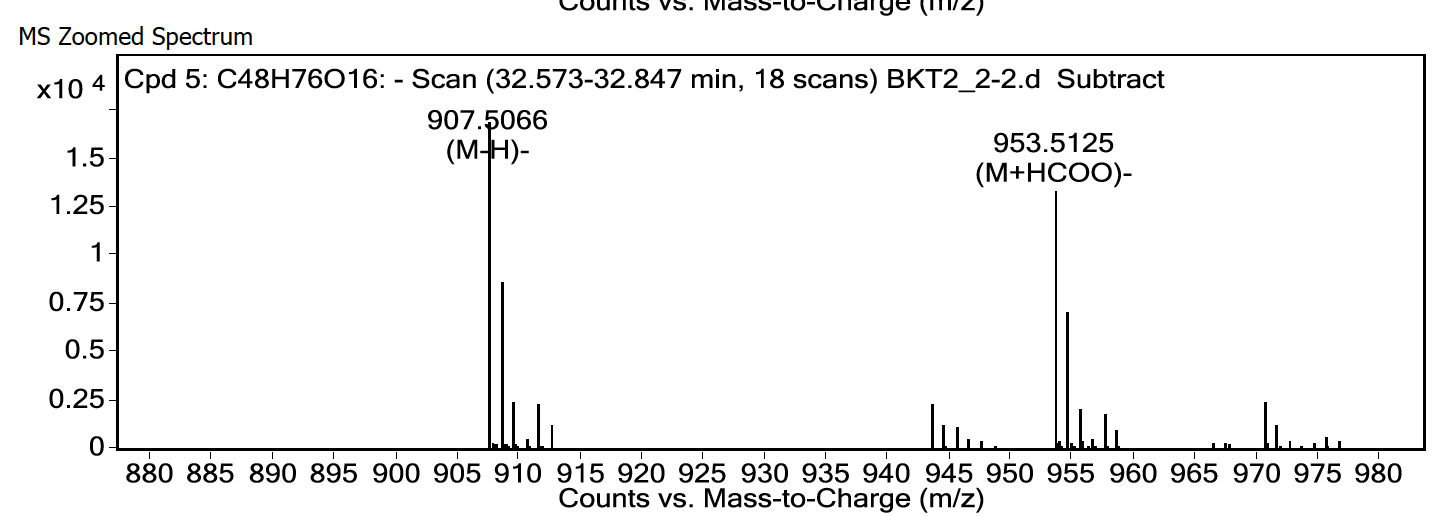

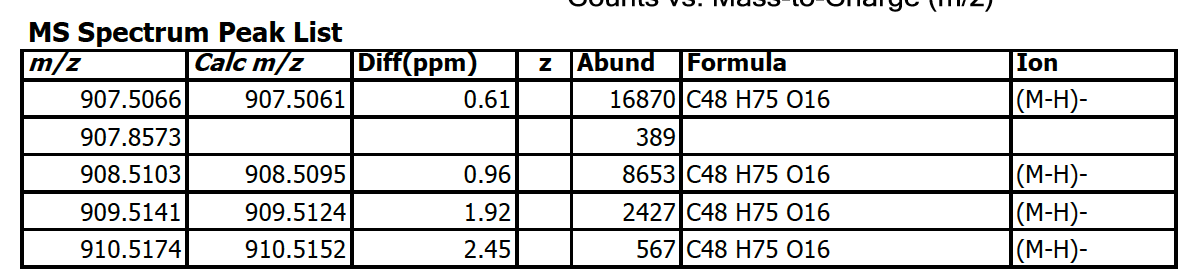


Figure S42. Structure and HRESIMS of compound 9

Figure S43. ^1^H NMR spectrum (Pyridine-d*_5_*, 600 MHz) of compound 9

Figure S44. ^13^C NMR spectrum (Pyridine-d*_5_*, 150 MHz) of compound 9


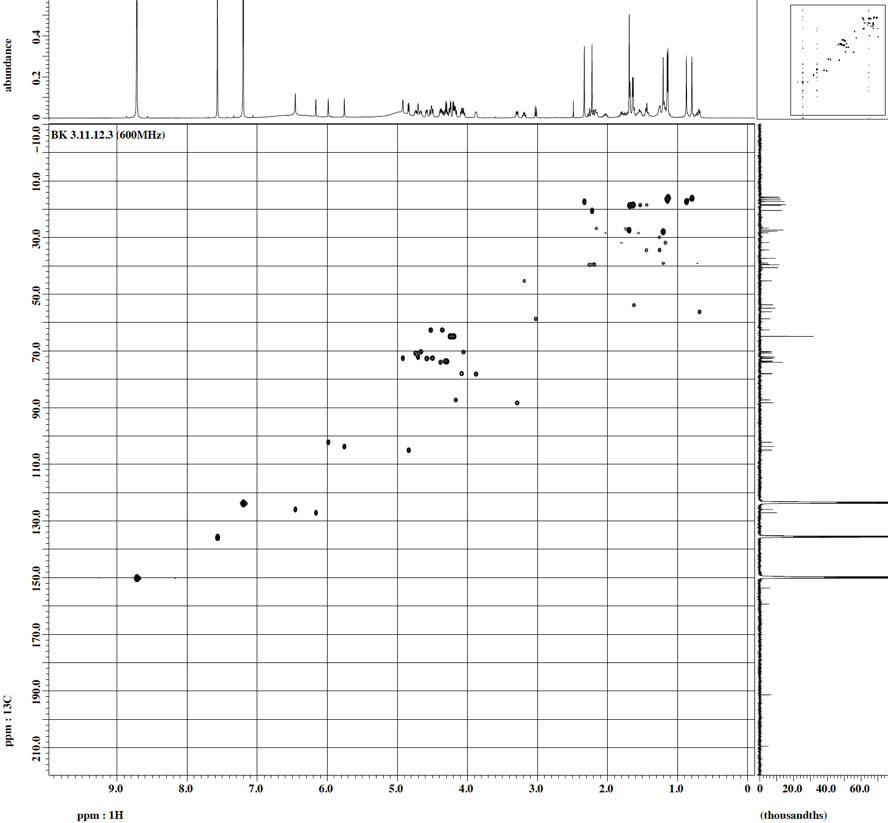


Figure S45. HSQC (H→C) spectrum (Pyridine-d*_5_*, 600 MHz) of compound 9

Figure S46. HMBC (H→C) spectrum (Pyridine-d*_5_,* 600 MHz) of compound 9


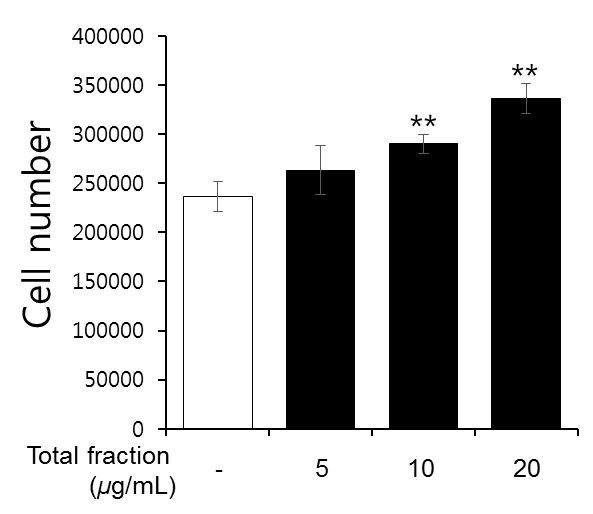


**Figure S47.** Effect of EtOH 95% fraction from *G. longipes* on C2C12 myoblast cells proliferation using the counting cell method.

C2C12 myoblast cells were seeded onto 36 mm cell culture dishes and incubated with tested fraction for 48 hours. Cells were then harvested and evaluated by a Countess^TM^ automated cell counter. Each value was expressed as the mean ± SD (*n*=3), * *p* < 0.05 and ** *p* < 0.01, compared to negative control group.


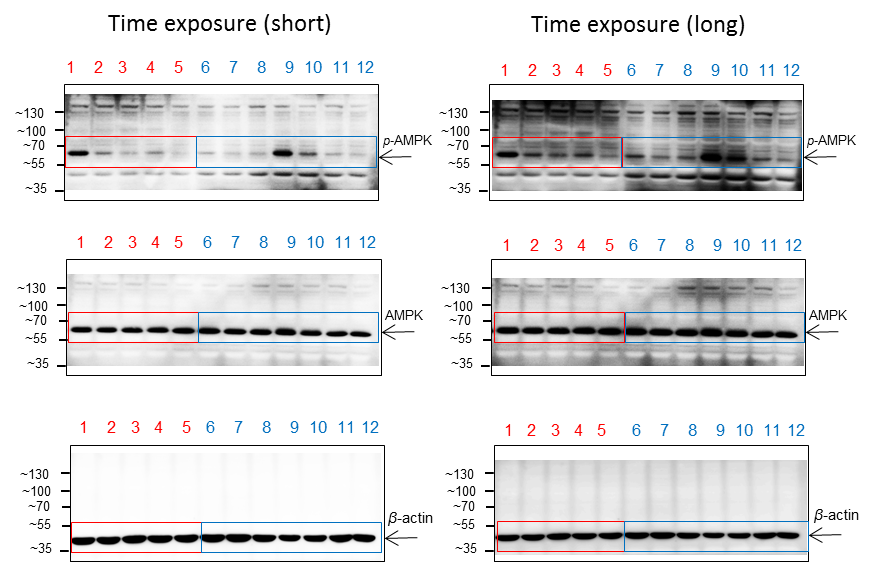


**Figure S48.** Activation effect of the SP70-EtOH 95% fraction from *G. longipes* on the phosphorylation (Thr172) of AMPK*α*; original uncropped blots.

(Red box) mouse C2C12 myotubes were incubated with the SP70-EtOH 95% fraction for 30 minutes. (Blue box) The cells were preincubated with or without compound C for 15 minutes. Then, the cells were exposed to the SP70-EtOH 95% fraction for 30 minutes. After that, cell lysates were lysed and Western blot was carried out to measure the expression of *p*-AMPK. Equal amounts of proteins were loaded on SDS-polyacrylamide gels. After transferring to PVDF membrane, the expressions of *p*-AMPK in the membrane were first detected. After primary and secondary antibodies were removed using Restore^TM^ Western blot stripping buffer (Thermo Sci.), membranes were continually incubated with AMPK and *β*-actin antibodies. Sample names were as follows: (Red box from 1−5) Fraction 40, 20, 10 *μ*g/mL, Aicar 0.2 mM, Ctrl; (Blue box from 6−12) Fraction 40 *μ*g/mL + Comp. C 20 *μ*M, Aicar 0.2 mM + Comp. C 20 *μ*M, Comp. C 20 *μ*M, Fraction 40 *μ*g/mL, Aicar 0.2 mM, Ctrl1, Ctrl2.


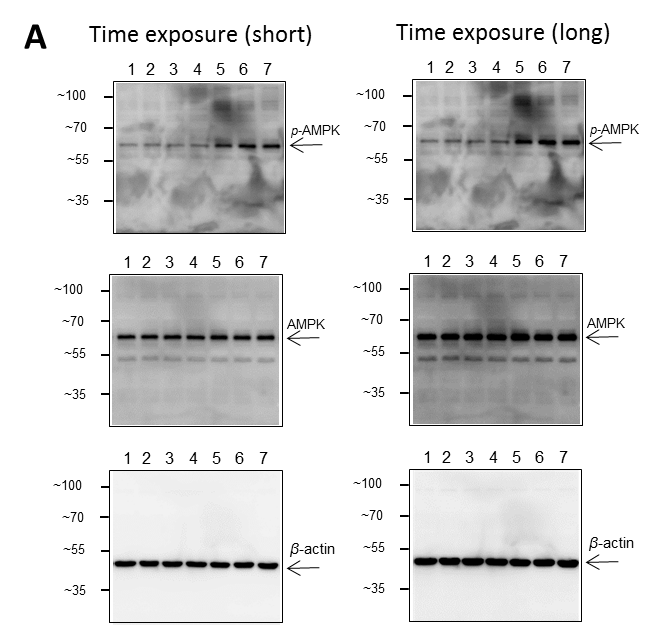


**Figure S49.** The effects of co-treatment compound C with the active fraction or compound **1** on *p*-AMPK (Thr172) in C2C12 myoblasts; original uncropped blots.

Mouse C2C12 myoblasts were re-incubated with compound C (20 *μ*M) for 15 minutes and the cells were continuously exposed to test samples for 1 hour. Cell lysates were collected and the phosphorylation of AMPK protein was measured by Western blotting. Equal amounts of proteins were separated on 12% SDS-polyacrylamide gels and then transferred to PVDF membranes. The expression of *p*-AMPK was firstly detected. For removing bound primary and secondary antibodies, a Restore^TM^ Western blot stripping buffer (Thermo Sci.) was used. The membrane was then incubated with AMPK antibody and it was also measured by LAS4000 luminescent image analyzer. Finally, the blot was continuously stripped and incubated with *β*-actin antibody. Blots from three independent experiments were shown (**A**−**C**). Fig. A was included in the final analysis (Fig. 5D). Sample names were from 1−7 as follows: Ctrl, Comp. C (20 *μ*M), Comp. C (20 *μ*M) + fraction (20 *μ*g/mL), Comp. C (20 *μ*M) + **1** (20 *μ*M), Fraction (20 *μ*g/mL), **1** (20 *μ*M), Aicar 0.2 mM.


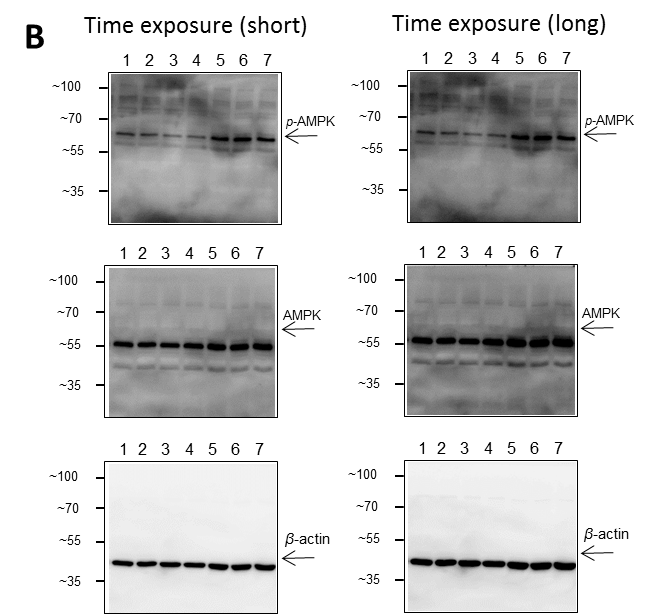


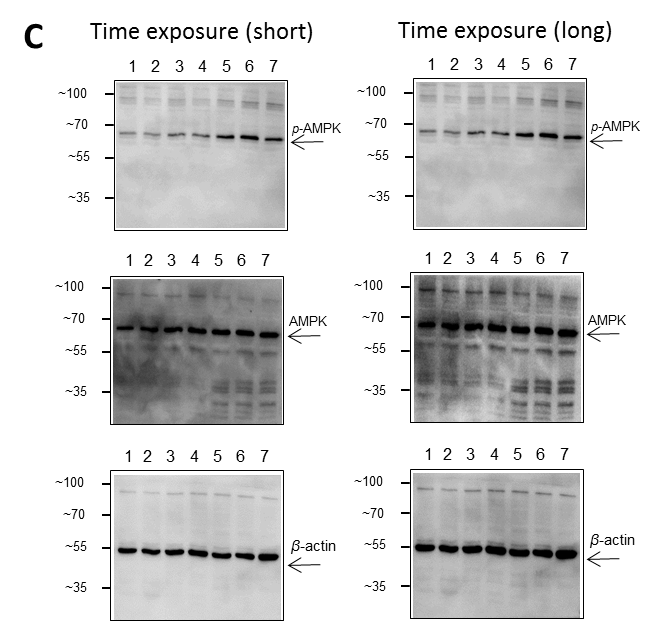


**Figure S49.** The effects of co-treatment compound C with the active fraction or compound **1** on *p*-AMPK (Thr172) in C2C12 myoblasts; original uncropped blots. (Continued)


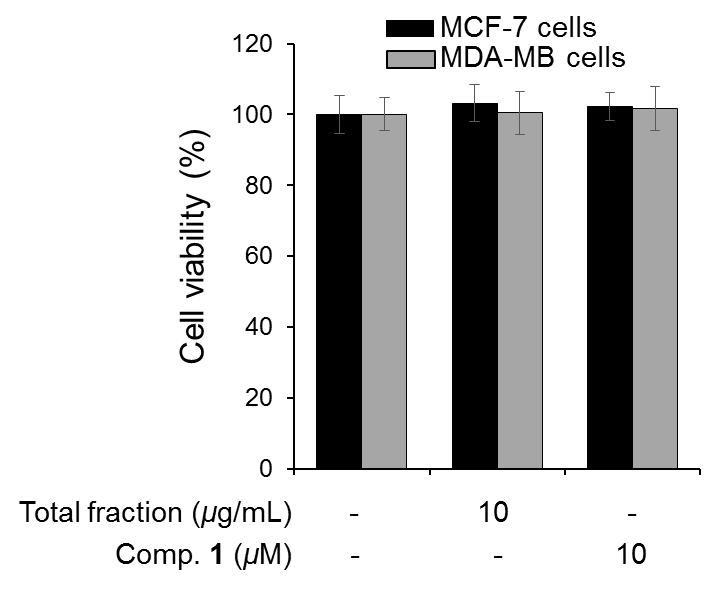


**Figure S50.** Effect of compound **1** on the proliferation of cancer cell lines (MCF-7 and MDA-MB 231 cells).

Cancer cells were incubated with test compound for 2 days. MTT assay was used to assess cell viability. Data are presented as the mean ± SD (*n*=3).


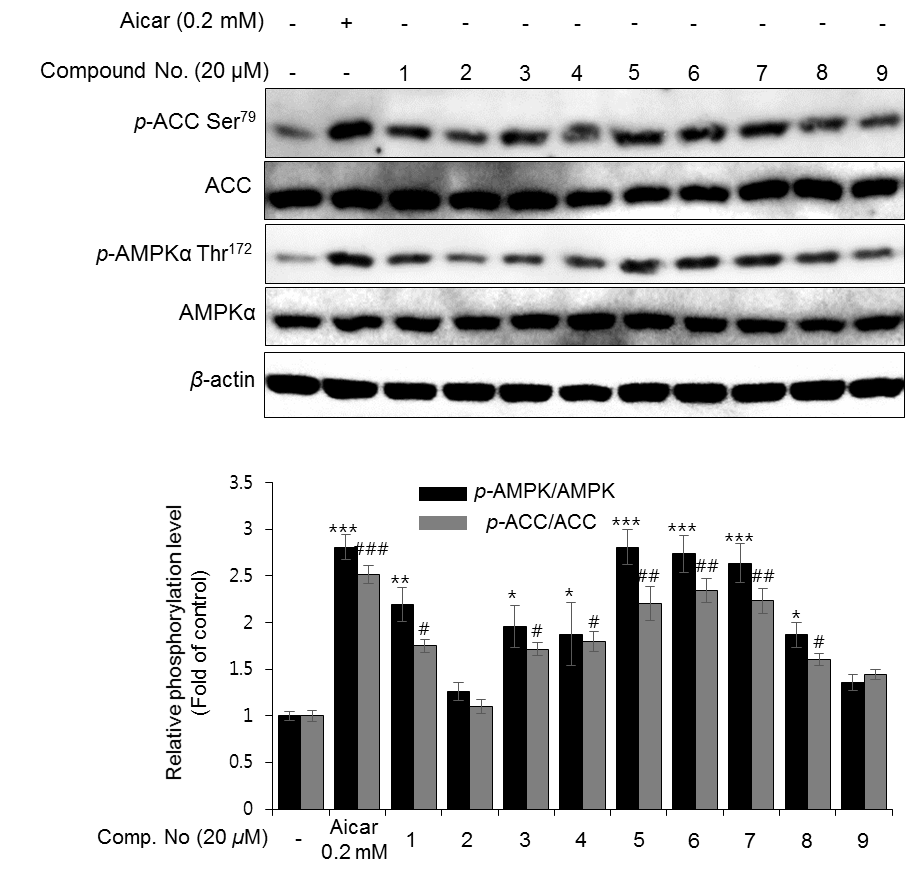


**Figure S51.** Stimulation effect of isolated compounds (**1**−**9**) on *p-*AMPK (Thr^172^) and *p-*ACC (Ser^79^) in mouse C2C12 myotubes**.**

Cells were incubated with compounds (20 *μ*M) or AMPK activator for 30 minutes. Phosphorylation of the target proteins was measured by Western blotting. Values were expressed as the mean ± SD (*n*=3), * *p* < 0.05, ** *p* < 0.01, and *** *p* < 0.001 compared to the negative control of *p-*AMPK, while ^#^ *p* < 0.05, ^##^ *p* < 0.01, and ^###^ *p* < 0.001, compared to the negative control of *p-*ACC.


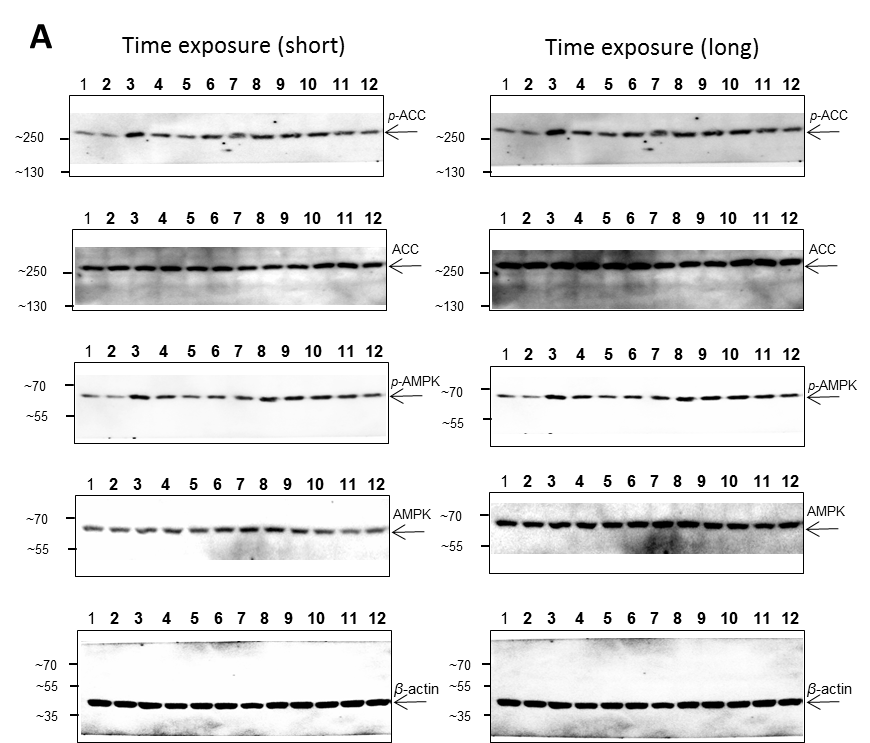


**Figure S52.** Stimulation effect of isolated compounds (**1**−**9**) on *p-*AMPK (Thr^172^) and *p-*ACC (Ser^79^) in C2C12 myotubes; original uncropped blots.

The cells were exposed to compounds (20 *μ*M) or Aicar for 30 minutes. Cell lysates were collected and the phosphorylation of the target proteins was measured by Western blotting. Aliquots of lysates were electrophoresed on 8% or 12% SDS-polyacrylamide gels. For detecting *p*-ACC Ser^79^ and ACC proteins, the 1× transfer buffer was added 0.5% SDS. The membranes were cut at different protein sizes. Then, cut-membranes were incubated with various antibodies (*p*-AMPK*α* Thr^172^, AMPK*α*, *p*-ACC Ser^79^, ACC and *β*-actin) and measured by LAS4000 luminescent image analyzer. Blots from three independent experiments were shown (A−C). Supplementary Fig. S53A was included in the final analysis (Supplementary Fig. S51). Sample names were from 1−12 as follows: Ctrl1, Ctrl2, Aicar 0.2 mM, **1−9** (20 *μ*M).


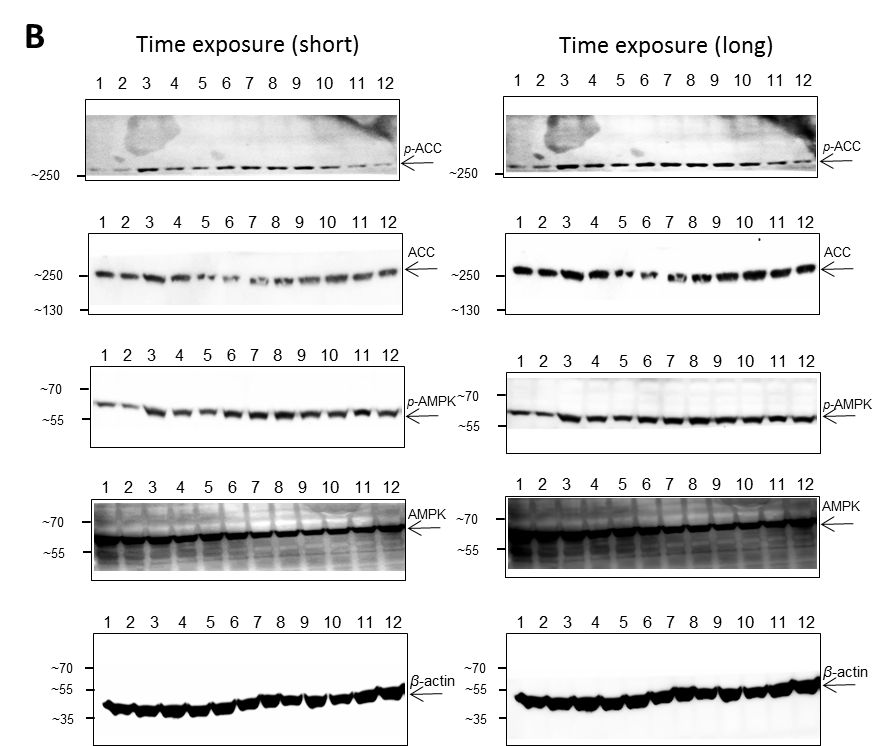


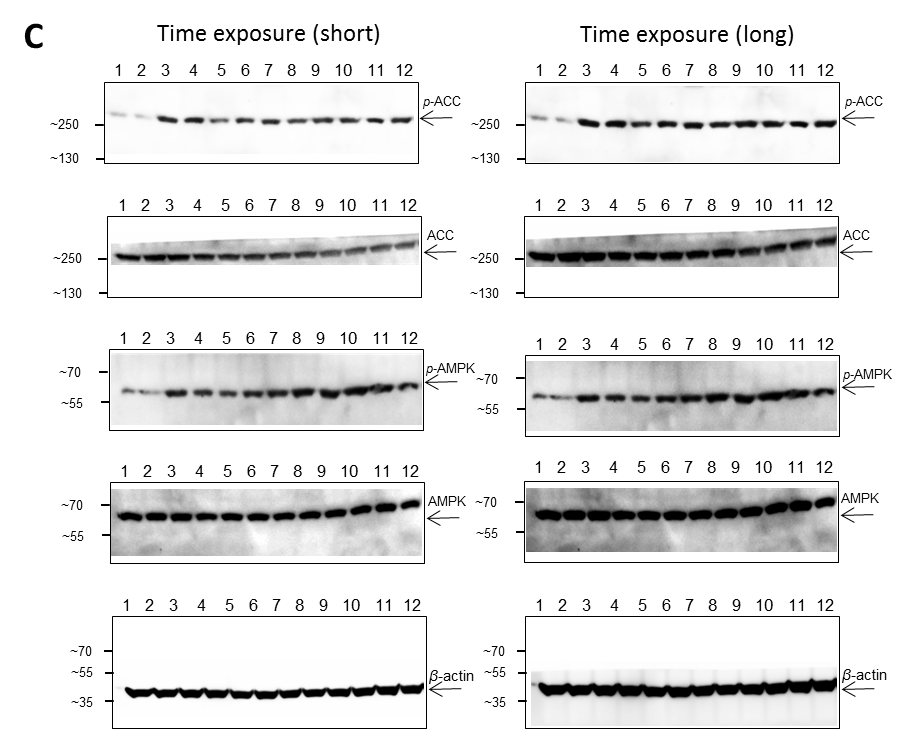


**Figure S52.** Stimulation effect of isolated compounds (**1**−**9**) on *p-*AMPK (Thr^172^) and *p-*ACC (Ser^79^) in C2C12 myotubes; original uncropped blots (continued)


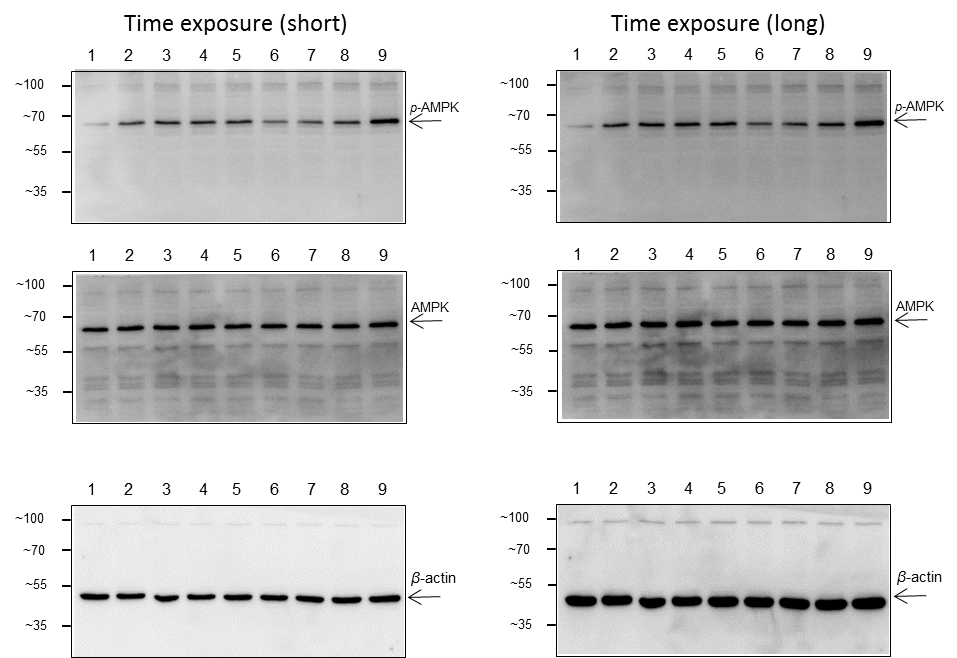


**Figure S53.** Stimulation effects of compounds **1**, **5**, **6**, **7**, and the active fraction on the expression of *p*-AMPK in mouse C2C12 myoblasts, original uncropped blots.

C2C12 myoblasts were incubated with test samples for 1 hour. The cell lysates were collected and equal amounts of proteins were loaded on 12% SDS-polyacrylamide gels. After transferred to PVDF membrane, the expression of *p*-AMPK was firstly detected using LAS4000 luminescent image analyzer. The membrane was then stripped and incubated with AMPK and *β*-actin antibodies, respectively. Sample names were from 1−9 as follows: Ctrl, **1**, **5**, **6**, **7** (20 *μ*M), Fraction (5, 10, 20 *μ*g/mL), Aicar (0.2 mM).


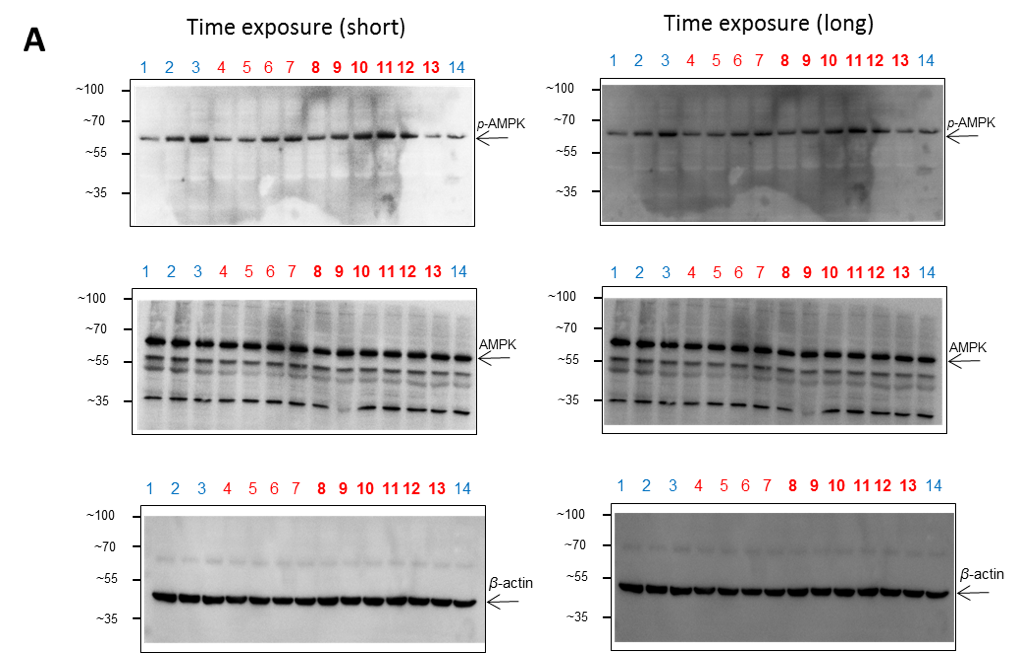


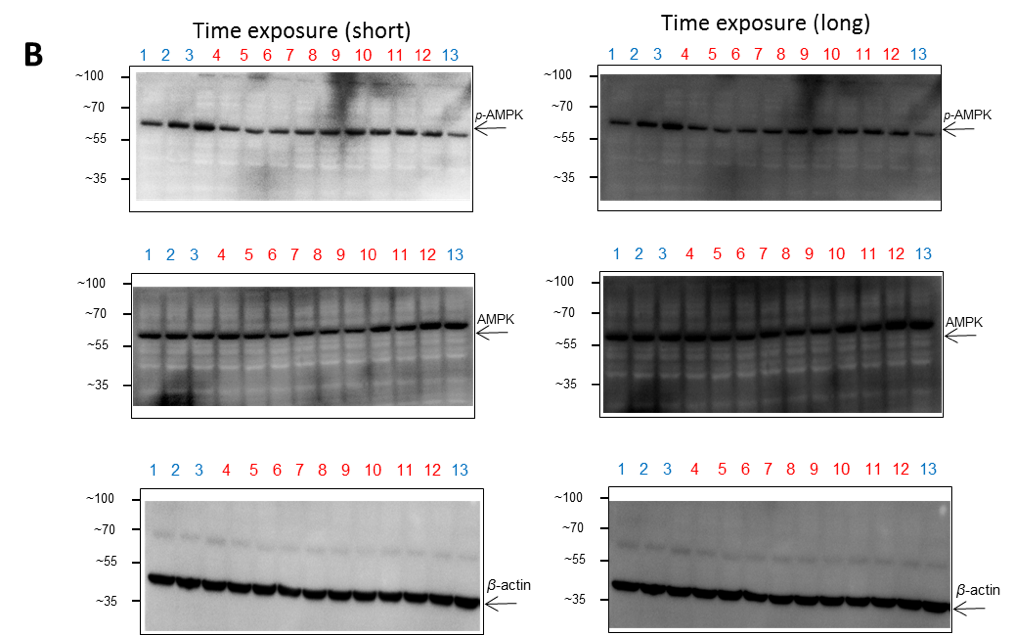


**Figure S54.** Time-dependent effect of compound **1** on the phosphorylation of AMPK protein; original uncropped blots.

C2C12 myoblasts were incubated with compound **1** (20 *μ*M) from 15 to 120 minutes. Phosphorylation of AMPK protein in the cells was assessed by Western blot analysis. Equal amounts of proteins were electrophoresed on SDS-polyacrylamide gels. The gels were transferred to PVDF membranes and the expression of *p*-AMPK was firstly detected. Using a Restore^TM^ Western blot stripping buffer (Thermo Sci.) for removing bound primary and secondary antibodies, the membrane was continually incubated with *β*-actin antibody. For detecting AMPK protein, equal amounts of proteins were loaded on other gels. Supplementary Fig. S54A was included in the final analysis (Fig. 5). (A) Sample names were from 4−13 as follows: (Red numbers - experiment 1) Ctrl-15, 30, 60, 90 and 120 min, Comp.**1**-15, 30, 60, 90 and 120 min; Samples names were from 1−3 and 14 as follows: (Blue numbers - experiment 3) Comp.**1**-15, 30 and 60 min, Ctrl. (B) Sample names were from 4−12 as follows: (Red numbers - experiment 2) Ctrl-30, 60, 90 and 120 min, Comp.**1**-15, 30, 60, 90 and 120 min; Samples names were from 1−3 and 13 as follows: (Blue numbers - experiment 4) Comp.**1**-15, 30, 60 min and Ctrl.


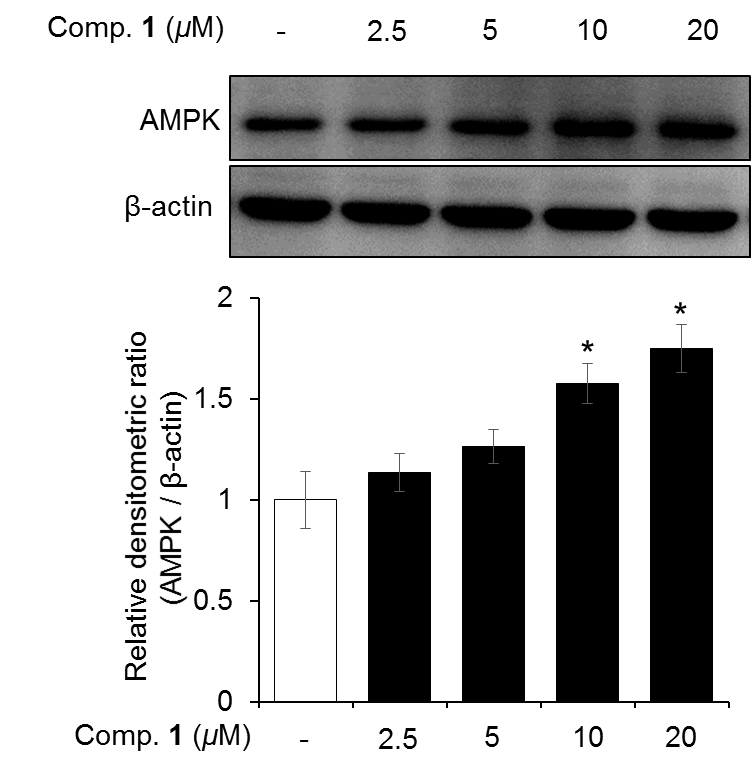


**Figure S55.** Compound **1** increased the expressions of AMPK*α* protein when C2C12 myoblast cells were incubated with compound **1** at different concentrations for 24 hours.

Data presented as the mean ± SD (*n* = 3); * *p* < 0.05, compared to negative control.


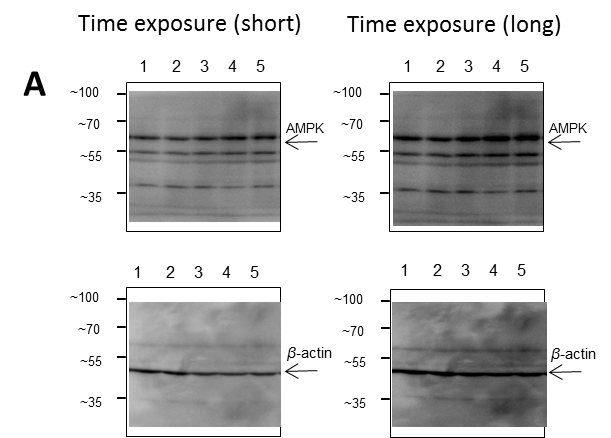


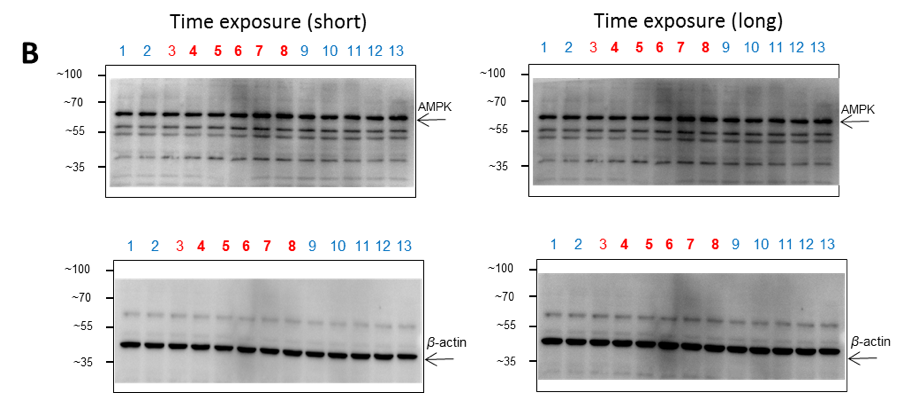


**Figure S56:** Effect of compound **1** on the expressions of AMPK*α* protein; original uncropped blots.

C2C12 myoblasts were exposed to compound **1** at different concentrations for 24 hours. The expressions of AMPK protein in the cells were evaluated using Western blot method. Equal amounts of proteins were loaded on SDS-polyacrylamide gels. After transferred to PVDF membranes and the expression of AMPK was firstly detected. Using a Restore^TM^ Western blot stripping buffer (Thermo Sci.) for removing bound primary and secondary antibodies, the membrane was continually incubated with *β*-actin antibody. Supplementary Fig. S56B was included in the final analysis (Supplementary Fig. S55). (A) Sample names were from 1−5 as follows (experiment 1): Ctrl, Comp.**1**-2.5, 5, 10 and 20 *μ*M. (B) Sample names were from 3−8 as follows: (Red numbers - experiment 2) Ctrl1, Ctrl2, Comp.**1**-2.5, 5, 10 and 20 *μ*M; Samples names were from 1−2 and 9−13 as follows: (Blue numbers - experiment 3) Ctrl1, Ctrl2, Comp.**1**-2.5, 5, 10, 20 *μ*M, Comp.**1**-20 *μ*M reload.


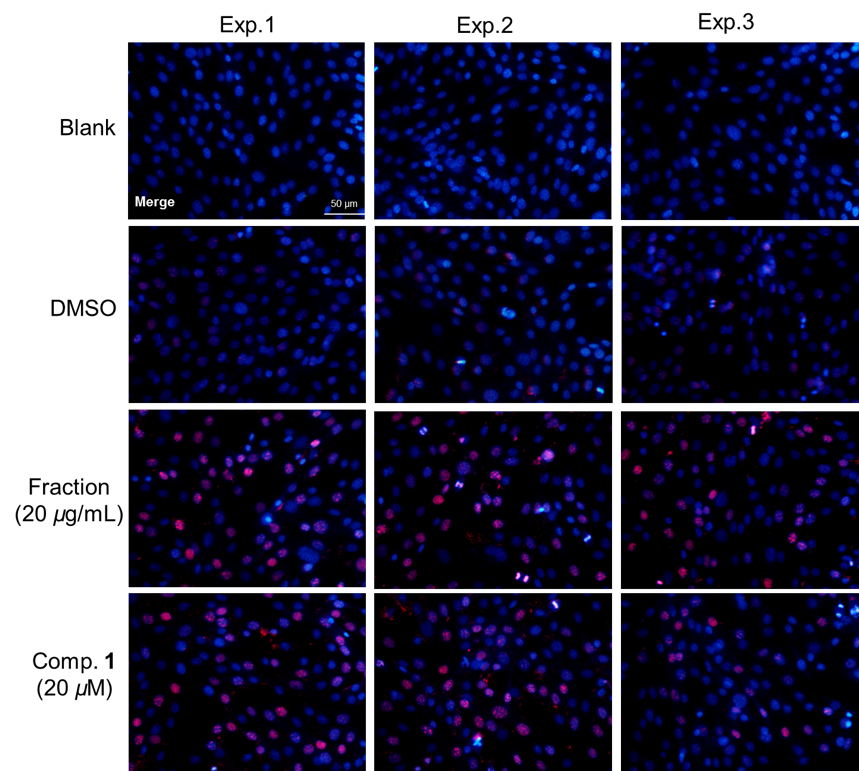


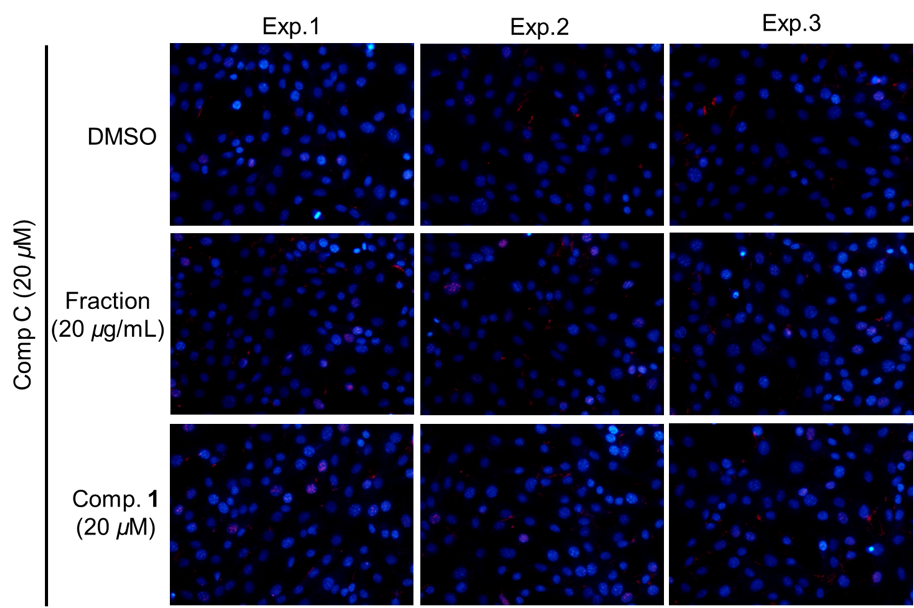


**Figure S57**: Effects of the active fraction and compound **1** on DNA synthesis during cell proliferation.

C2C12 myoblasts were re-treated with or without compound C (20 *μ*M) for 15 minutes and the cells were continuously exposed to the samples for 8 hours. The cultures were then incubated in the presence or absence of BrdU for 2 hours. The cells were fixed, permeabilized, and stained with anti-BrdU antibody and DAPI solution. Cells images were captured using fluorescence microscopy. Experiment 1 (exp.1) was included in the final analysis (Fig. 6A).


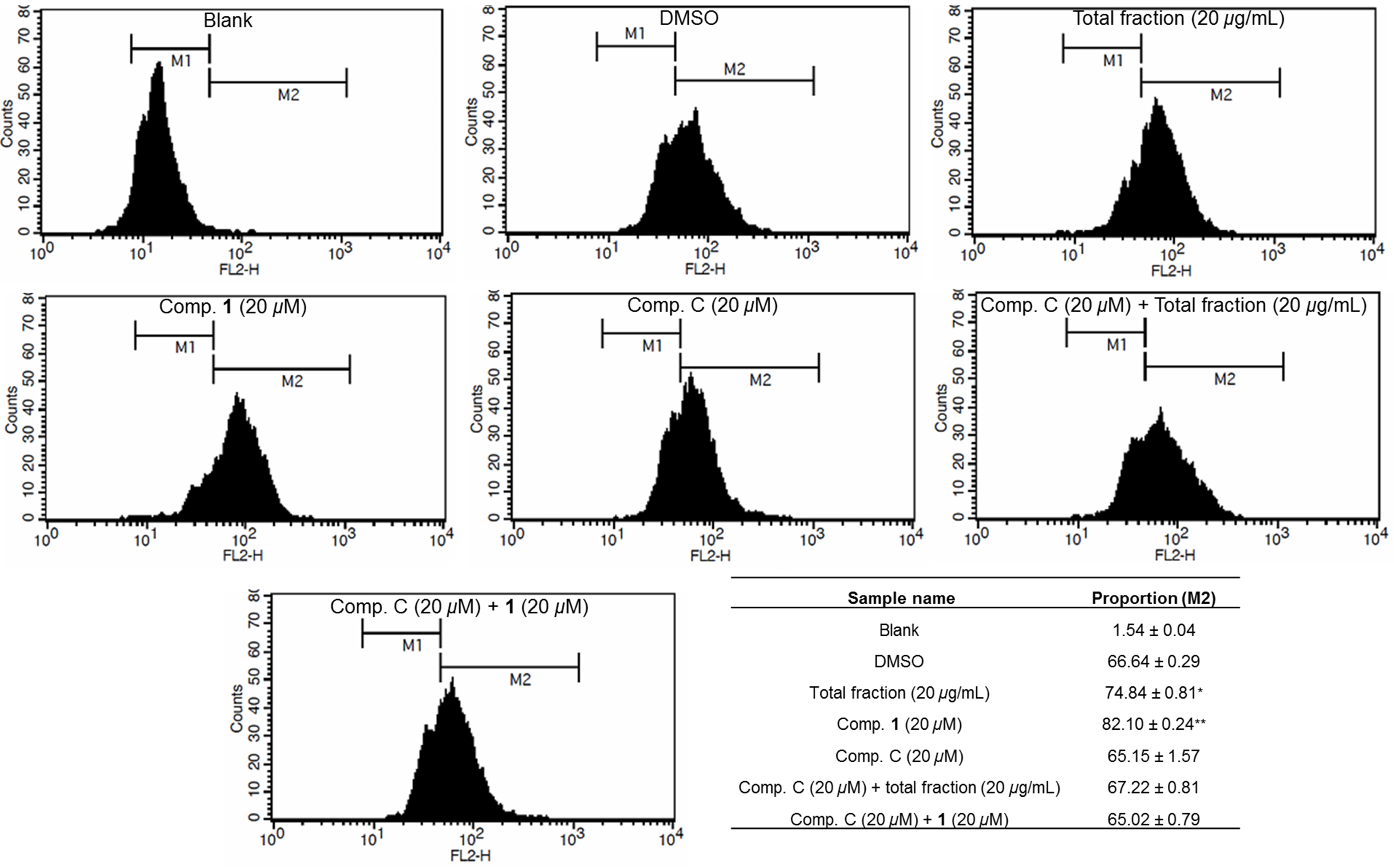


Figure S58. Effects of co-treatment compound C with the active fraction and compound 1 on DNA synthesis during cell proliferation.

Mouse C2C12 myoblasts were re-treated with or without compound C (20 *μ*M) for 15 minutes and then the cells were incubated with test samples. After 8 hours of incubation, the cells were exposed to BrdU for 2 hours and they were harvested using trypsin method. Flow cytometric analysis for BrdU staining was performed and the cell distribution was determined using a histogram. Results were calculated as the mean ± SD (*n* = 3), * *p* < 0.05 and ** *p* < 0.01, compared to the DMSO group.


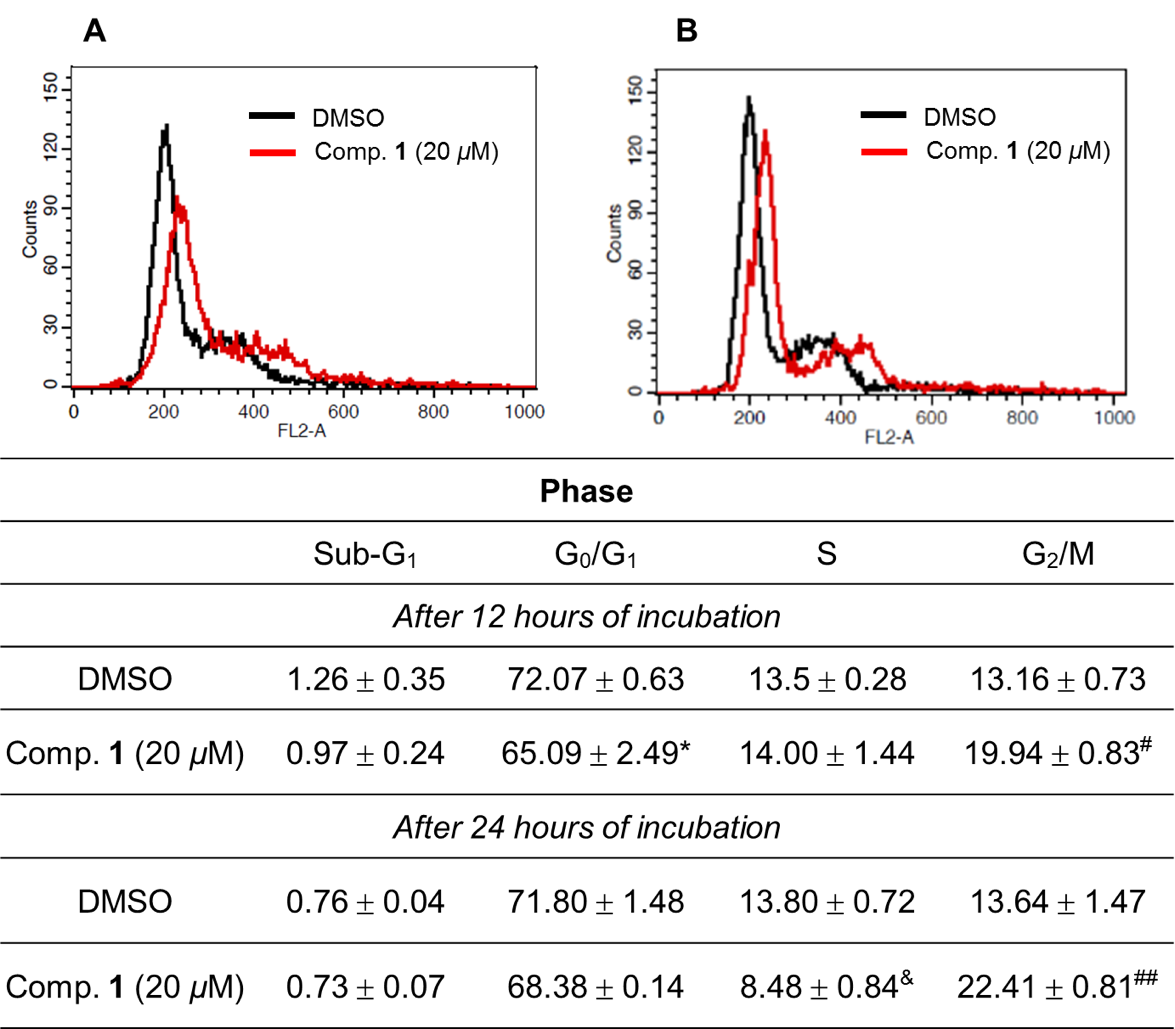


Figure S59. C2C12 myoblast cells were treated with compound 1 (20 *μ*M) and incubated for 12 hours (A) or 24 hours (B).

Cell cycle distribution at different phases was determined by flow cytometry staining with propidium iodide (PI). Cell distribution was analyzed by the histogram method with 2N DNA content (in G_0_/G_1_ phases) and > 2N DNA content (in S or G_2_/M phases). Values were expressed as the mean ± SD (*n* = 2), * *p* < 0.05 compared to negative DMSO control in G_0_/G_1_ phases,

^&^ *p* < 0.05 compared to DMSO in S phase, ^#^ *p* < 0.05 and ^##^ *p* < 0.01 compared to DMSO in the G_2_/M phase.
